# Simultaneous imaging of bidirectional guided waves probes arterial mechanical anisotropy, blood pressure, and stress synchronously

**DOI:** 10.1126/sciadv.adv5660

**Published:** 2025-08-06

**Authors:** Yuxuan Jiang, Guo-Yang Li, Keshuai Hu, Shiyu Ma, Yang Zheng, Mingwei Jiang, Zhaoyi Zhang, Xinyu Wang, Yanping Cao

**Affiliations:** ^1^ Institute of Biomechanics and Medical Engineering, AML, Department of Engineering Mechanics, Tsinghua University, Beijing 100084, China.; ^2^ Department of Mechanics and Engineering Science, College of Engineering, Peking University, Beijing 100871, China.; ^3^ Department of Cardiology, Peking University Third Hospital, Beijing 100191, China.; ^4^ NHC Key Laboratory of Cardiovascular Molecular Biology and Regulatory Peptides, Peking University, Beijing 100191, China.; ^5^ Beijing Key Laboratory of Cardiovascular Receptors Research, Beijing 100191, China.

## Abstract

Arterial biomechanical indicators have long been recognized as fundamental contributors to the physiology and pathology of cardiovascular systems. Probing multiple biomechanical parameters of arteries simultaneously throughout the cardiac cycle is highly important but remains challenging. Here, we report a method to quantify arterial anisotropic stiffness, arterial wall stresses, and local blood pressure in a single measurement. With programmed ultrasound excitation and imaging, arterial axial and circumferential guided waves were simultaneously induced and measured in the longitudinal view. Then, a mechanical model was proposed to quantitatively predict the correlation of arterial guided waves with arterial biomechanical parameters. Our experimental design and biomechanical model enable an elastography method to assess temporal variations in blood pressure, bidirectional stiffness, and mechanical stresses in arterial walls. In vivo experiments were performed on healthy young, normotensive older, and hypertensive older volunteers. The results demonstrate that our method can find applications in understanding aging of cardiovascular system and diagnosis of cardiovascular diseases.

## Teaser

Ultrasound imaging of arterial mechanical anisotropy, blood pressure, and stress offers insights into cardiovascular health.

## INTRODUCTION

Arterial biomechanical indicators including arterial stiffness, blood pressure, and mechanical stresses in the arterial wall have long been recognized as fundamental contributors to the physiology and pathology of cardiovascular system. Arterial stiffness is profoundly affected by aging and various cardiovascular diseases (CVDs), including diabetes, hypertension (HT), and hypercholesterolemia (*1*, *2*). Adversely, large-artery stiffening will cause isolated systolic HT, left ventricular dysfunction, and target organ damage (*3*). Therefore, arterial stiffness has been used as a risk factor for various CVDs including HT, diabetes, and atherosclerosis (*4*–*7*). Blood pressure is another key biomarker in cardiovascular system, and its monitoring and management are essential for patients with HT (*8*). It has been recognized that the blood pressure and arterial stiffness have a complex interplay, leading to an insidious feedback loop in the progression of CVDs (*3*). Mechanical stresses in the arterial wall stem from residual stresses required to guarantee the physiological functions of the artery and prestresses caused by blood pressure, which are sensed by vascular cells and regulate the homeostasis of arteries (*9*, *10*). Changes of mechanical stress will alter the homeostasis, leading to arterial remolding and disease progression (*11*). Excessive arterial stress increases the risk of aortic dissection (*12*) and aneurysms rupture (*13*). Given the importance of these biomechanical indicators for diagnosis and therapy of CVDs, their measurements in vivo have long been pursued over past years.

Arteries are fiber-reinforced materials and exhibit mechanical anisotropy (i.e. distinct variations in axial and circumferential stiffness) (*14*). Diverse pathologies have been linked to the changes of arterial anisotropy (*15*). In clinics, pulse wave velocity (*5*), arterial compliance, and stiffness index (*16*, *17*) enable the assessment of the equivalent stiffness of arteries in a specific direction. Characterizing the anisotropic stiffness of arteries in vivo remains challenging. The arterial guided wave elastography method developed recently holds promise for characterizing arterial anisotropic stiffness (*18*). In this method, the arterial wall is stimulated by focused acoustic radiation forces (ARFs), and the induced elastic wave propagates in a guided way confined by the arterial wall, which can be detected with ultrafast ultrasound imaging. By imaging guided waves along the axial direction (*19*–*27*) or in cross-sectional plane (*28*, *29*), arterial guided wave elastography has the capability to gauge arterial axial or circumferential stiffness, respectively. The methods reported in the literature image axial and circumferential guided waves separately by rotating the ultrasound probe (*30*, *31*); therefore, it remains challenging to accurately measure the anisotropy in arterial stiffness because the arterial stiffness is spatially dependent and varies throughout cardiac cycles due to blood pressure pulsations. To overcome this challenge, it is necessary to image the axial and circumferential guided waves simultaneously at the same position of the artery.

Blood pressure leads to circumferential mechanical stress in the arterial wall (*32*, *33*), and axial stress is mainly attributed to the prestresses caused by the interaction of the artery with surrounding soft tissues (*34*, *35*). Emerging evidence indicates that axial stress notably contributes to vascular homeostasis and aids in minimizing overall tissue stress in normal arteries (*11*). Measuring arterial circumferential stress is straightforward using the Young-Laplace equation (*36*), whereas the assessment of axial stresses requires the prior knowledge of arterial axial stiffness and axial deformation (*34*, *37*), which are difficult to achieve in vivo. Our previous work has shown that programmed elastic waves enable probing mechanical stress in soft materials without prior knowledge of constitutive models (*38*) and variation of local blood pressure is related to variation of guided waves (*20*). Therefore, local blood pressure and mechanical stresses in the artery at different moments of the cardiac cycle are possible to be inferred, provided that the axial and circumferential guided waves at the same position of the artery can be measured simultaneously.

In this study, we report a guided wave elastography method for arteries, in which the axial and circumferential guided waves are successfully generated with an exclusively programmed ARF. We then develop an image processing algorithm and an acoustoelastic model incorporating tissue viscoelasticity to infer arterial anisotropic stiffness, local blood pressure, and mechanical stresses in the arterial wall in a single measurement. We conducted in vivo experiments on young (*n* = 30), older (*n* = 14), and HT (*n* = 8) volunteers to demonstrate the potential use of our method in clinics. The ability to measure arterial anisotropic stiffness, local blood pressure, and mechanical stresses in the arterial wall simultaneously at a local position of the artery at different time points of the cardiac cycle not only helps understand the relationship between these crucial biomechanical indicators but also facilitates their application in clinics for the diagnosis and therapy of CVDs.

## RESULTS

### Generating and imaging axial and circumferential guided waves simultaneously in the longitudinal ultrasound imaging view

**Fig. 1.**
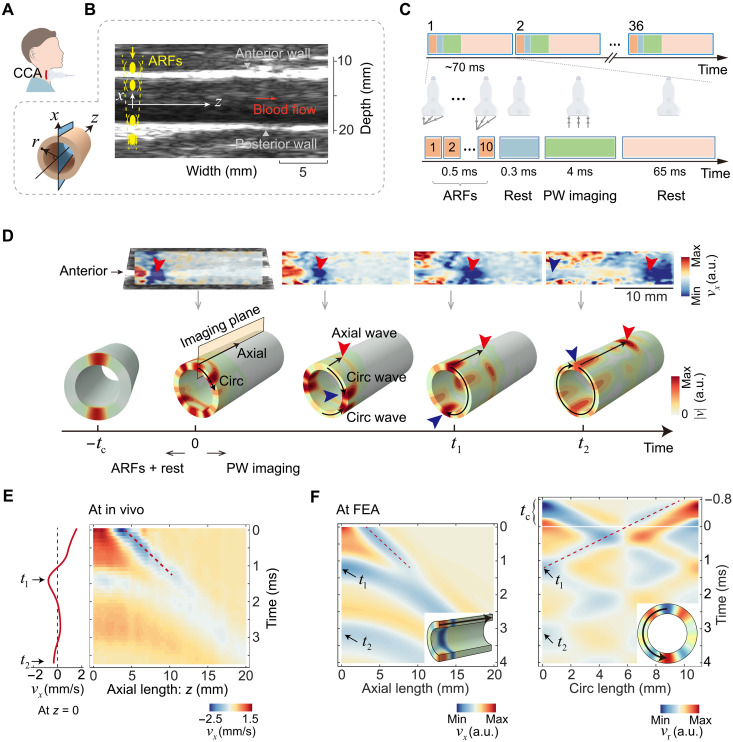
Excitation and measurement of axial and circumferential guided waves. (**A**) Experiment set-up to measure human right CCAs. (**B**) Ultrasound imaging and excitation method. (**C**) Imaging sequence. PW, plane wave. (**D**) Particle velocity maps in the longitudinal view over time from the in vivo experiment (top) and accompanied schematics showing the bidirectional wave propagation (bottom). Circ, circumferential; a.u., arbitrary units. (**E**) Particle spatiotemporal velocity map extracted along the axial path on the anterior wall at end-diastole from the in vivo experiment. (**F**) Simulation results of the bidirectional guided waves, including velocity maps extracted along the axial path (left) and along the circumferential path (right). The dashed lines in the left and right panels denote wave propagation of axial and circumferential guided waves, respectively.

Our approach was built on remote excitation of bidirectional guided waves with programmed ARF and on imaging the wave motions in the axial (longitudinal) view of the human right common carotid artery (CCA), as shown in Fig. 1A. We used a linear ultrasonic transducer to apply 10 transient focused ultrasound beams to the proximal side of the artery, sequentially from the anterior wall to the posterior wall in a time span of 0.5 ms (Fig. 1B). After a short rest (0.3 ms), the same transducer resumed plane wave imaging at a frame rate of 10 kHz in 4 ms to capture wave propagation. This push-rest-imaging mode was repeated 36 times, with a resting duration of 65 ms between each consecutive measurement. Totally, the 36 ultrasound measurements took ~2.5 s at equal intervals, spanning around three cardiac cycles (Fig. 1C).

Figure 1D (top) shows the snap shots of the particle velocity map for a healthy young volunteer (27 years old, male). Figure 1E depicts the spatiotemporal map of the particle velocity extracted along the centerline of the anterior wall. The front of the axial guided wave is tracked by the red dashed line (the slope denotes the axial group wave velocity *c*_a_). Circumferential guided wave manifests itself in two minima in the time profile of the particle velocity at *z* = 0. We denote the corresponding time points as *t*_1_ and *t*_2_, respectively (Fig. 1E). To understand the emergence of the circumferential guided wave in the axial view, we performed finite element analysis (FEA) on a prestressed viscoelastic anisotropic tube immersed in fluid (see FEA model in the “Finite element analysis” section). As shown in the bottom of Fig. 1D, the circumferential guided wave originating from the posterior wall follows a semicircular path, arriving at the anterior wall at time *t*_1_. Subsequently, at time *t*_2_, the circumferential guided wave emanating from the anterior wall completes its journey, returning to its initial point. The particle velocity map in the longitudinal direction from the FEA results closely matches the experimental result (left of Fig. 1F). The particle velocity map in the circumferential direction demonstrates that the guided wave excited from the posterior wall reaches the anterior wall at time *t*_1_ (dashed line in the right of Fig. 1F). We also performed an in vivo comparative experiment where only the anterior wall of the artery was excited (see note S1 and fig. S1). In this case, no negative peak was observed at time *t*_1_ on the anterior wall, while one was present on the posterior wall, further verifying the assumption.

Since the time *t*_1_ corresponds to the arrival of the circumferential guided wave originating from the posterior wall, we can determine the circumferential group wave velocity *c*_c_ through *c*_c_ = π*r*_c_ / (*t*_1_ + *t*_c_), where *t*_c_ denotes the compensation time (0.8 ms; accounting for the duration of ARFs and rest stages) and *r*_c_ is the middle radius of the artery.

A phantom experiment was conducted to investigate the influence of material viscoelasticity on the measurement of circumferential guided wave group velocities. Unlike arteries, the phantom material exhibited more elastic behavior, leading to significant differences in the spatiotemporal particle velocity map—particularly unfeasible to resolve time *t*_1_ at *z* = 0 (note S2 and fig. S2). This is primarily because viscoelasticity makes arteries act as a low-pass filter that attenuates high-frequency elastic waves, resulting in weak dispersion and thereby enabling accurate identification of time *t*_1_ (note S2 and fig. S3). Fortunately, because of the viscoelastic nature of arteries, we are able to measure the circumferential group velocity in the longitudinal view.

### Dynamic changes of bidirectional guided waves throughout cardiac cycles in young, older, and hypertensive participants

**Fig. 2.**
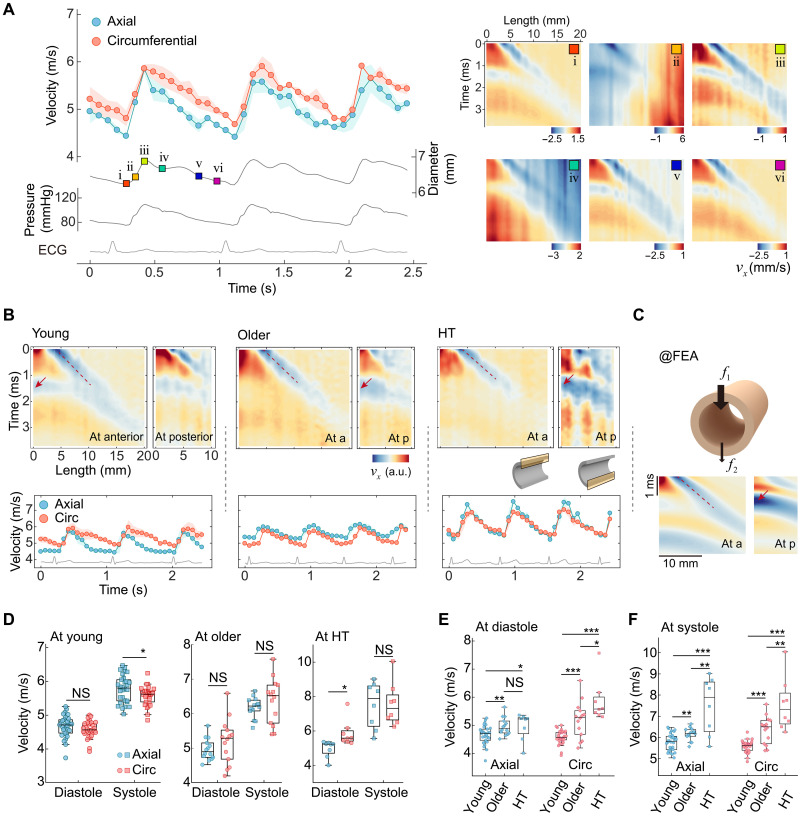
Dynamic variation of the bidirectional group wave velocities in the young, older, and hypertensive groups. (**A**) Left: Variation of the bidirectional group wave velocities in cardiac cycles from a young volunteer. The synchronous arterial diameter, blood pressure (measured by applanation tonometry), and ECG are also plotted. Right: The spatiotemporal particle velocity maps in various cardiac phases. (**B**) Top: Representative particle velocity maps extracted from the anterior and posterior walls at end-diastole for volunteers in the three groups. Bottom: The corresponding bidirectional group wave velocities in cardiac cycles. a, anterior; p, posterior. (**C**) FEA to verify the differences in spatiotemporal velocity maps between anterior and posterior walls in the older and hypertensive volunteers. *f*_1_, the ARF applied on the anterior wall; *f*_2_, the ARF applied on the posterior wall. *f*_1_ / *f*_2_ = 5. (**D**) Statistics of the bidirectional group wave velocities at diastole and systole in the young, older, and hypertensive groups, respectively. (**E**) Comparison of bidirectional group wave velocities at diastole and (**F**) systole among the three groups. NS, not significant. *, 0.01 ≤ *P* ≤ 0.05; **, 0.001 ≤ *P* < 0.01; ***, *P* < 0.001.

Figure 2A presents the dynamic variation of axial and circumferential group wave velocities throughout cardiac cycles in a young healthy volunteer (27 years old, male), alongside synchronized variations in arterial diameter, blood pressure, and electrocardiogram (ECG). The axial and circumferential group wave velocities vary simultaneously with diameters and the blood pressure. The right panel of Fig. 2A shows six snapshots of the particle velocity at specific cardiac phases. Even during time points such as midsystole (state ii) and near the dicrotic notch (state iv), when the arterial wall exhibits prominent displacements due to the pulse wave, the axial and circumferential guided wave signals can still be resolved. We also found that the time phase *t*_1_ varied inversely with the diameter: It reached the minimum when the diameter was at its maximum and vice versa (fig. S4). This inverse relationship helps resolve the dynamic variations in circumferential group wave velocities.

To demonstrate the in vivo feasibility of the proposed ultrasound method, we recruited healthy young volunteers (*n* = 30, 20 ± 2 years old), normotensive older volunteers (*n* = 14, 51 ± 8 years old), and hypertensive older volunteers (*n* = 8, 55 ± 5 years old). Figure 2B (top) presents particle velocity maps at end-diastole for young, older, and hypertensive participants. We observed that for most older participants (11 of 14) and all hypertensive participants (8 of 8), the time phase *t*_1_ was less pronounced on the anterior wall, whereas it could be detected on the posterior wall (see Fig. 2B and fig. S5). Therefore, by using the guided wave information from both the anterior and posterior walls, we were able to measure the dynamic changes of bidirectional group wave velocities among multiple participant groups (bottom of Fig. 2B). The difference of particle velocity maps between the anterior and posterior walls in older and hypertensive arteries can be attributed to their strong ultrasound scattering and attenuation, resulting in a much weaker ARF applied on the posterior wall than that on the anterior wall (the ratio of ARF on the posterior wall to the anterior wall could be less than 0.25 in hypertensive arteries; see details in note S3). Consequently, a stronger circumferential signal is detected on the posterior wall, corresponding to guided waves originating from the anterior wall. Simulations with a greater force applied on the anterior wall than the posterior wall also confirm the experimental observations in the older and hypertensive participants (Fig. 2C).

Figure 2D displays the statistical results of bidirectional group wave velocities at diastole (corresponding to the minimum velocity within a single cardiac cycle) and systole (corresponding to the maximum velocity within a single cardiac cycle) in the young, older, and hypertensive groups, respectively. The values of group wave velocities are listed in Table 1. The axial wave velocities agree well with those reported in previous studies (*20*, *21*). In most cases, the axial and circumferential wave velocities exhibit no significant difference, except in the young group at diastole and the hypertensive group at systole (*P* < 0.05). The similarity of bidirectional group wave velocities contrasts with the mechanical anisotropy of arteries, which will be investigated in the “Bidirectional guided wave imaging enables synchronous measurement of bidirectional stiffness and mechanical stress in arterial walls” section.

**Table 1.** Results of bidirectional group wave velocities in the three participant groups.

	Young (*n* = 30)	Older (*n* = 14)	HT (*n* = 8)
$c_{a,d}$ (m/s)	4.67 ± 0.32	4.96 ± 0.31	4.97 ± 0.48
$c_{a,s}$ (m/s)	5.78 ± 0.40	6.15 ± 0.30	7.52 ± 1.33
$c_{c,d}$ (m/s)	4.58 ± 0.25	5.20 ± 0.65	5.86 ± 0.75
$c_{c,s}$ (m/s)	5.58 ± 0.28	6.35 ± 0.66	7.55 ± 1.25

Figure 2E compares the bidirectional group wave velocities at diastole among the three participant groups. Both axial and circumferential wave velocities increase with aging. Unexpectedly, we find that HT further elevates circumferential wave velocities but not significantly changes the axial wave velocities. This may ascribe to the remodeling of arteries in response to the elevated circumferential stresses introduced by HTs, leading to the increase in circumferential arterial stiffness that accelerates the circumferential wave velocities (*39*). Figure 2F compares the wave velocities at systole. Unlike the case at diastole, the axial wave velocities in hypertensive older volunteers are higher than those of the normotensive older volunteers (*P* < 0.01).

### Circumferential guided wave group velocity as a robust index for probing continuous blood pressure

**Fig. 3.**
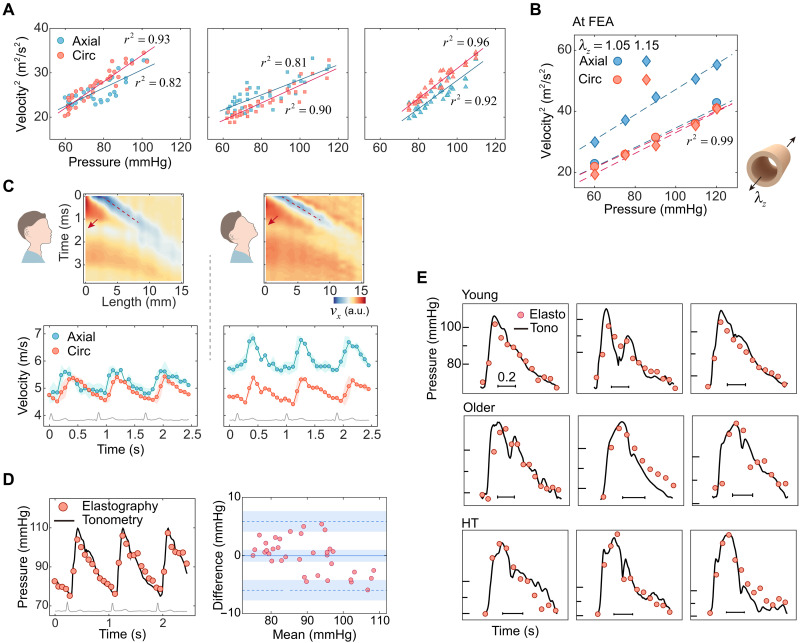
Blood pressure measurement using the ultrasound elastography method. (**A**) Relationship of the squares of the bidirectional group wave velocities and blood pressure from three young volunteers. (**B**) FEA results to verify the linear relationship between the squares of bidirectional velocities and blood pressure and the variation of bidirectional wave velocities in the two postures. (**C**) A comparative experiment conducted in two postures: normal posture (a forward gaze) and craning posture (stretching his/her neck). Particle velocity maps at end-diastole for the normal posture (top left) and for the craning posture (top right). Bidirectional wave velocities for the normal posture (bottom left) and for the craning posture (bottom right). (**D**) Blood pressure measurement of a young volunteer using the ultrasound method and the applanation tonometry method (left). Bland-Altman plot of the two methods (right). (**E**) Blood pressure measurement results of nine volunteers across the young, older, and hypertensive groups. Elasto, elastography; Tono, tonometry.

As illustrated in Fig. 3A, the squares of both the axial and circumferential group wave velocities from three volunteers are linearly correlated to the blood pressures. The FEA results in Fig. 3B also demonstrate this linear correlation. This linear relation enables the quantitative measurement of blood pressure using either axial or circumferential group wave velocities. Despite previous reports suggesting that axial group wave velocities are useful for probing blood pressure (*20*), we find that circumferential group wave velocities offer greater robustness, as demonstrated in the following. We conducted a comparative experiment on a young volunteer (27 years old, male) involving two different postures. The volunteer was first instructed to sit upright with a forward gaze (referred to as the normal posture) and then to extend his neck fully by looking upward to the utmost (referred to as the craning posture), during which the ultrasound elastography was performed. The measurements were repeated three times for each posture. The left of Fig. 3C shows the particle velocity map at diastole in normal posture and the corresponding bidirectional group wave velocities. The right of Fig. 3C shows the particle velocity map in craning posture and the corresponding bidirectional group wave velocities. As shown, the circumferential wave velocity remains almost unchanged (normal, 4.9 ± 0.3 m/s; craning, 4.9 ± 0.3 m/s; means ± SD denote the group velocities averaged over cardiac cycles), while the axial wave velocity increases by ~17% with neck craning (normal, 5.2 ± 0.3 m/s; craning, 6.1 ± 0.4 m/s). This can be attributed to the fact that craning leads to a more pronounced axial stretch of the CCA, while blood pressure remains stable during two successive and short-term experiments conducted under different postures. FEA results in Fig. 3B further confirm this trend: When axial stretch increases, axial wave velocities rise significantly, while circumferential wave velocities remain stable. Another comparative experiment was performed on a young volunteer (24 years old, male) in supine and sitting postures to induce a transient change in carotid blood pressure (~10 to 20 mmHg) (*40*). The circumferential group velocity was ~8% higher in the supine posture (5.2 ± 0.3 m/s) than in sitting (4.8 ± 0.3 m/s), while the axial velocity remained stable (supine, 4.5 ± 0.4 m/s; sitting, 4.6 ± 0.2 m/s) (see details in note S4 and fig. S6). As a result, circumferential wave velocity is sensitive to blood pressure and less affected by the factors such as neck extension, making it a robust and more suitable index used in our method for measuring blood pressure.

Figure 3D presents blood pressure measurement results using the circumferential group wave velocities, alongside applanation tonometry (*41*), a gold standard technique for noninvasive continuous blood pressure measurement. We adopted the Bland-Altman plot to assess the consistency between the two methods. The biases are −0.06 mmHg in average, with 95% limits of agreement of −6.0 to 5.9 mmHg. The statistical results show that the consistency of the methods is good (B-A plots for other participant groups are shown in fig. S7). Figure 3E shows more results of blood pressure measurement among the three groups. In general, both waveform and amplitude of the blood pressure can be effectively captured by the ultrasound elastography method. Compared to tonometry, this method eliminates the need for vascular compression, reducing potential measurement artifacts and improving patient comfort. In addition, unlike the methods that infer blood pressure by measuring arterial geometry (*42*, *43*), this approach is less affected by postural factors such as neck extension, offering the potential for more accurate blood pressure measurements in clinical applications.

### Bidirectional guided wave imaging enables synchronous measurement of bidirectional stiffness and mechanical stress in arterial walls

It is well recognized that arterial biomechanics is highly nonlinear, stress dependent, and viscoelastic. We therefore developed a mechanical model for bidirectional guided waves in arteries. Noting that the duration of wave propagation (approximately microsecond) is much shorter than the cardiac cycle (approximately second) and the deformation involved in wave propagation (approximately micrometer) is much smaller than that caused by pulse wave motion (approximately millimeter), it is reasonable to model the elastic wave motion as an infinitesimal deformation imposed on a static finite deformation, which can be described by the following motion equation (*44*)

$$\nabla \cdot \Sigma =\rho u_{,\mathrm{tt}}$$ (1)

where **Σ** denotes incremental stress induced by elastic waves, **u** denotes the displacement induced by elastic wave motions, ρ is the density of arteries, and *t* is the time. The subscript comma indicates partial derivative of the variable. For harmonic waves, $u=u_{0}e^{i\left(k\cdot x-\mathrm{\omega t}\right)}$ , where $u_{0}$ , k , and ω denote wave amplitude, wave vector, and angular frequency, respectively. For guided waves in arteries, we seek the relation between ω and wave number k=∣k∣ , which determines the dispersion relations and phase velocities. The arterial wall was modeled as a single-layer, nonlinear viscoelastic hollow cylinder. Since the wavelength of guided waves (~6 mm) is much larger than the wall thickness (~1 mm), a single-layer model was adopted with effective stiffness equivalent to that of the three-layered arterial wall (see details in note S5). Blood fills the interior, while the perivascular tissues are so soft that they can be approximated as fluid, as supported by our previous work (*45*). With these boundary conditions, we obtain the phase velocity of the axial guided waves $c_{a}^{p}$ as

$$c_{a}^{p}=\frac{\omega }{\text{Re}\left(k\right)}{\left\{1-{\left[\frac{N}{r_{c}\text{Re}\left(k\right)}\right]}^{2}\right\}}^{-\frac{1}{2}}$$ (2)

The relation between the angular frequency ω and the wave number *k* implicitly depends on viscoelastic parameters of the artery $\alpha_{a}$ , γ , *g*, and τ (where $\alpha_{a}$ and γ describe the arterial nonlinear elasticity and *g* and τ describe the arterial viscosity) and wall thickness *h* (explicitly expressed by Eq. 10; see the “Guided axial and circumferential wave model for prestressed viscoelastic arteries” section). The additional term in the square brackets is introduced to correct the curvature effect (*N* = 2; see the “Guided axial and circumferential wave model for prestressed viscoelastic arteries” section and notes S6 and S7). The phase velocity of the circumferential guided waves $c_{c}^{p}$ is determined by

$$c_{c}^{p}=\frac{\omega }{\text{Re}\left(k\right)}$$ (3)

where the relationship of ω and *k* implicitly depends on viscoelastic parameters of the artery $\alpha_{c}$ , γ , *g*, and τ (where $\alpha_{c}$ describe the arterial nonlinear elasticity in the circumferential direction) and wall thickness *h* (explicitly expressed by Eq. 16; see the “Guided axial and circumferential wave model for prestressed viscoelastic arteries” section).

In the dispersion relations of guided axial and circumferential waves, we note the arterial stiffness and stress implicitly come into play through the stiffness parameters $\alpha_{a}$ and $\alpha_{c}$ , respectively. To demonstrate, we reformulate $\alpha_{a}$ and $\alpha_{c}$ to decouple them as $\alpha_{a}=\sigma_{a}+\mu_{\text{zr}}\lambda_{r}$ and $\alpha_{c}=\sigma_{c}+\mu_{\mathrm{\theta r}}\lambda_{r}$ , where $\sigma_{a}$ and $\sigma_{c}$ denote axial and circumferential stresses, respectively; $\mu_{\text{zr}}$ and $\mu_{\mathrm{\theta r}}$ denote tangent shear moduli at the stretching state; and $\lambda_{r}$ is the radial stretch ratio (see details in note S8). The equations indicate the shear moduli and stresses jointly affect the parameters $\alpha_{a}$ and $\alpha_{c}$ and thus the dispersion relations.

In experiments, the spatiotemporal data of the particle velocity are sampled with high temporal (frame rate, 10 kHz) and spatial resolution (lateral resolution, ~0.3 mm). Therefore, we can extract the full dispersion relation for the axial elastic waves by Fourier transforming the spatiotemporal data to the wave number–frequency space. We find that windowing the spatiotemporal field can effectively mitigate the influence of circumferential guided wave signals on the estimation of axial guided wave dispersion (see the “Measurement of phase velocity of axial guided waves” section and note S9). The dispersion relation for circumferential waves, however, cannot be recovered because of the limited spatial resolution (only a single acquisition) in the circumferential direction.

**Fig. 4.**
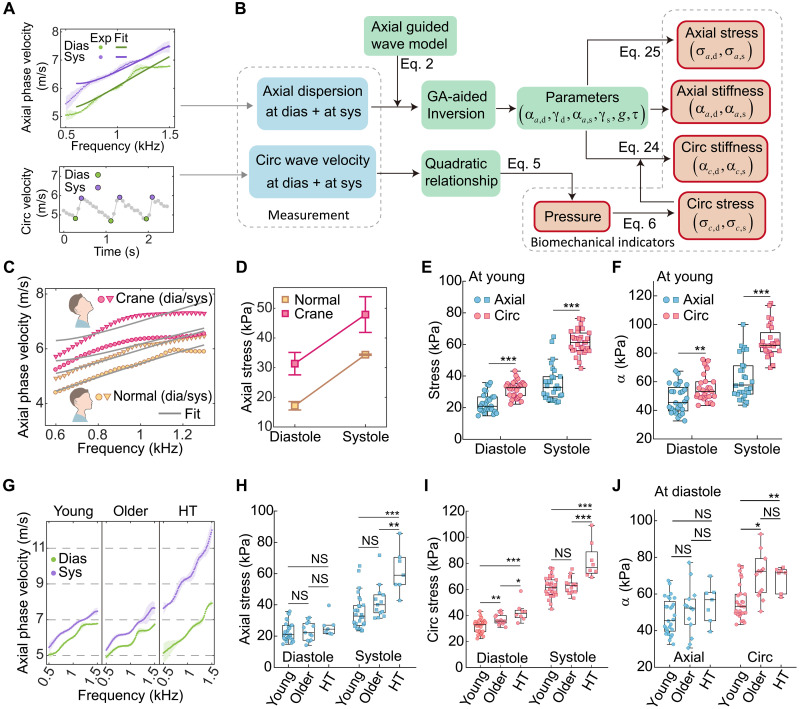
Characterization of arterial bidirectional stresses and stiffnesses. (**A**) Top: In vivo measurement of axial guided wave dispersion at diastole (representing the minimum wave dispersion within a single cardiac cycle) and systole (representing the maximum wave dispersion within a single cardiac cycle), along with their fitting curves. Bottom: In vivo measurement of circumferential wave velocities in cardiac cycles. Dias, diastole; Sys, systole. (**B**) Flowchart to characterize arterial biomechanical indicators, including blood pressure, bidirectional stresses, and bidirectional stiffnesses. GA, genetic algorithm. The subscripts d and s in α, γ, and σ denote diastole and systole, respectively. (**C**) Dispersion curves for the two postures (normal and craning) and their fitting curves. (**D**) Axial stresses in the two postures at diastole and systole. The axial stresses at diastole and systole in normal posture are 17.2 ± 1.4 and 34.4 ± 0.2 kPa, respectively. The axial stresses at diastole and systole in craning posture are 31.3 ± 3.8 and 47.9 ± 6.0 kPa, respectively. (**E**) Comparison of bidirectional stresses at diastole and systole, respectively, in the young group. (**F**) Comparison of bidirectional stiffnesses at diastole and systole, respectively, in the young group. (**G**) Wave dispersion of axial guided waves from three volunteers across the young, older, and hypertensive groups. (**H**) Comparison of axial stress among the three groups. (**I**) Comparison of circumferential stress among the three groups. (**J**) Comparison of axial stiffness and circumferential stiffness at diastole among the three groups.

We then consider extracting arterial bidirectional stiffnesses $\alpha_{a}$ and $\alpha_{c}$ and bidirectional stresses $\sigma_{a}$ and $\sigma_{c}$ from the guided wave motions. Figure 4 (A and B) depicts our method. Briefly, we aim to obtain $\alpha_{a}$ and γ by fitting the dispersion relations of axial guided waves using a genetic algorithm–assisted inverse method (see the “Genetic algorithm–assisted inversion to infer mechanical parameters of arteries” section). Then, we can directly measure axial stress using $\sigma_{a}=\alpha_{a}-\gamma$ (see the “Relationship between atrial stress and incremental parameters” section). In the circumferential direction, the circumferential stress $\sigma_{c}$ is accessible by measuring blood pressure via circumferential group wave velocities and calculating it using the Young-Laplace equation (*36*) (see the “Measurement of circumferential stress of arteries” section). The circumferential stiffness, $\alpha_{c}$ , then can be recovered by using $\alpha_{c}=\sigma_{c}+\gamma$ (see the “Relationship between atrial stress and incremental parameters” section).

To verify this inverse method, we performed an ex vivo experiment on a porcine aorta. The estimation errors for both the axial stress and axial stiffness, compared to the uniaxial tensile test, were less than 10% (see note S10). Then, we applied our method to the in vivo data under both normal and craning postures. As shown in Fig. 4 (C and D), the axial dispersion significantly increases from the normal state to the craning state, with the variation in axial stress between the two postures measured at ~13 kPa. Existing methods for measuring arterial axial stress rely on known arterial constitutive model parameters and axial deformation (*34*, *35*), which are indeed difficult to measure in vivo. Our method overcomes this limitation and enables the direct measurement of arterial axial stress through elastic waves, potentially enhancing the accuracy of axial stress measurement.

Figure 4E shows the in vivo results of bidirectional stresses in the young participant group. Significant difference was found between the axial and circumferential stresses both at diastole (*P* < 0.001) and systole (*P* < 0.001), where circumferential stress is higher than the axial ones. Figure 4F compares the bidirectional stiffnesses $\alpha_{a}$ and $\alpha_{c}$ in the young group. Significant difference was observed between $\alpha_{a}$ and $\alpha_{c}$ both at diastole (*P* < 0.01) and systole (*P* < 0.001), with circumferential stiffness being approximately 1.2 to 1.5 times higher than axial stiffness.

Figure 4G shows dispersion curves of axial guided waves at diastole and systole among the three groups. Inferred values of arterial mechanical parameters are listed in Table 2. Figure 4 (H and I) compares the axial and circumferential stresses among the three groups, respectively. The circumferential stress increases with aging and HT, which is expected given the elevated blood pressure in the older and hypertensive groups. In contrast, the axial stress remains largely stable with aging and HT at diastole; this may be due to tissue-level responses (such as thickening and axial shortening) to restore wall axial stress toward homeostatic targets (*11*). Axial stresses measured in the normotensive participants are consistent with previously reported values (*34*). Figure 4J compares the axial and circumferential stiffness across the three groups, respectively. At diastole, axial stiffness showed no significant differences between the three groups, while circumferential stiffness in the older group was higher than that in the young group (*P* < 0.05). This indicates that circumferential stiffness of arteries undergoes more pronounced changes with aging compared to axial stiffness. The current method for characterizing bidirectional stiffnesses and stresses in arteries holds promise for providing a more comprehensive understanding of arterial aging and HT.

**Table 2.** Inferred values of the arterial mechanical parameters in the three participant groups. Note that the viscous parameter τ ranges from ~3 × 10^−5^ to 8 × 10^−5^ s in the three groups.

	$\alpha_{a,d}$ (kPa)	$\alpha_{a,s}$ (kPa)	$\alpha_{c,d}$ (kPa)	$\alpha_{c,s}$ (kPa)	$\gamma_{d}$ (kPa)	$\gamma_{s}$ (kPa)	*g*	$\sigma_{a,d}$ (kPa)	$\sigma_{a,s}$ (kPa)	$\sigma_{c,d}$ (kPa)	$\sigma_{c,s}$ (kPa)
Young (*n* = 30)	47.8 ± 9.8	62.3 ± 14.8	55.6 ± 9.5	88.1 ± 11.3	24.1 ± 6.6	26.8 ± 6.3	0.77 ± 0.08	22.8 ± 6.1	35.2 ± 11.0	31.7 ± 5.1	61.5 ± 7.6
Older (*n* = 14)	51.1 ± 13.8	73.3 ± 17.8	63.8 ± 10.1	94.7 ± 12.6	27.2 ± 8.3	31.4 ± 9.2	0.76 ± 0.11	23.1 ± 5.9	42.1 ± 11.1	36.8 ± 3.8	62.7 ± 6.1
HT (*n* = 8)	54.9 ± 10.3	94.0 ± 15.6	68.8 ± 6.7	113.8 ± 11.6	26.5 ± 6.8	32.1 ± 9.5	0.80 ± 0.06	26.2 ± 6.3	61.7 ± 14.2	42.6 ± 7.3	81.7 ± 13.8

## DISCUSSION

Arterial anisotropic stiffness, local blood pressure, and mechanical stresses within the arterial wall are intertwined indicators, crucial for understanding cardiovascular physiology and pathology (*1*, *3*). The interplay among these parameters not only governs arterial wall mechanics but also directly affects the progression of CVDs. Historically, in vivo attempts have been made to investigate arterial axial stiffness (*19*–*21*, *27*), circumferential stiffness (*29*), bidirectional stiffnesses (*46*), local blood pressure (*42*, *43*, *47*, *48*), and axial stress (*34*, *35*). Previous techniques typically assess these biomechanical indicators separately and are unable to capture their dynamic interrelations, which may ascribe to the temporal and spatial variabilities of arterial mechanics. In this study, we overcome these limitations by developing an ultrasound elastography method that uses programmed ARF to excite both the anterior and posterior walls of the artery. This approach allows for the simultaneous evaluation of axial and circumferential guided waves by imaging the tissue particle vibration along the arterial long axis. By combining advanced acoustoelastic modeling and image processing techniques, our method enables the synchronous assessment of anisotropic stiffness, local blood pressure, and mechanical stresses in arteries in a single measurement. The clinical utility of our method has been demonstrated via in vivo experiments on healthy young, normotensive older, and hypertensive older volunteers. The results and findings highlight the capability of our method for providing a more comprehensive understanding of age- and disease-related changes in arterial biomechanics, enabling more accurate risk assessment, early disease detection, and personalized treatment strategies for patients with cardiovascular conditions.

The changes in arterial anisotropic stiffness are closely related to the distribution of collagen fibers (*49*). While many ex vivo experiments have characterized arterial anisotropy (*50*–*52*), discrepancies between in vivo and ex vivo conditions demand the measurement of artery anisotropy in vivo. The dynamic variations in arterial stiffness throughout the cardiac cycle and across different spatial locations pose substantial challenges for in vivo characterization. By integrating the dispersion relation of the axial guided waves with blood pressure measurements, bidirectional arterial stiffness can be assessed at specific cardiac phases and anatomical locations. This approach overcomes the limitation posed by the absence of a circumferential dispersion relation. Experimental results indicate that the circumferential stiffness of the right CCA is approximately 1.3 times higher than its axial stiffness (Fig. 4F). Unlike bidirectional stiffnesses, the group velocities in both directions, as directly extracted from spatiotemporal maps, are relatively close (Fig. 2D). This is primarily due to the distinct guided wave modes in the two directions (see details in note S11). The variations in bidirectional group wave velocities across groups (Fig. 2E) also exhibit slight differences from the trends observed in bidirectional stiffnesses (Fig. 4J), primarily because group velocity is influenced by multiple factors, such as stiffness, frequency, and geometry (see details in note S11). This underscores the advantage of using arterial stiffness as a biomechanical index for evaluating vascular health because arterial stiffness reflects the actual mechanical cues sensed by vascular cells, whereas group velocities can be influenced by confounding factors such as arterial geometry. The proposed method paves the way for future investigations into the diagnostic and prognostic utility of arterial bidirectional stiffnesses.

Arterial stress, both axial and circumferential, plays a critical role in determining the mechanical behavior of the arterial wall and is closely linked to the progression of CVDs (*33*, *37*). Traditional methods for measuring axial stress often rely on the known material parameters and deformation (*34*, *35*), which are indeed difficult to achieve in vivo. In contrast, our method provides a direct means for measuring axial stress by analyzing the dispersion of axial guided waves without prior knowledge of deformation or constitutive models. Using the current method, we estimated that axial stress in the right CCA increases by ~13 kPa during neck extension. We also found that axial stress in the older group did not exhibit significantly higher levels compared to the young group. With the proposed method, measured stresses represent the combined effects of smooth muscle contraction and fiber deformation. Further studies are needed to distinguish the active and passive components. The ability to assess both axial and circumferential stresses in real time during cardiac cycles opens up possibilities for understanding the mechanobiology of arterial disease and could potentially affect the management of conditions such as aortic dissection, aneurysm, and HT.

In addition to its implications for arterial biomechanics, our method enables noninvasive continuous blood pressure measurement, leveraging the linear correlation observed in this study between the square of circumferential wave velocities and blood pressure. Unlike conventional techniques such as applanation tonometry, our method eliminates the need for vascular compression, thereby reducing measurement-induced artifacts and enhancing patient comfort. In addition, compared to the methods that estimate blood pressure based on arterial geometry measurement (*42*, *43*), the present method is less affected by neck extension. According to our experiments, when the neck is fully extended, the diameter of the right CCA reduces by ~0.2 mm (a ~3% decrease in diameter) compared to the forward gaze, whereas the circumferential wave velocities remain stable under varying neck postures (less than a 1% decrease in velocity). This makes circumferential wave velocities a promising index for continuous, noninvasive, and reliable blood pressure monitoring, particularly when integrated with wearable ultrasonic devices (*42*, *43*, *53*, *54*).

Beyond cardiovascular research and clinical applications, our method could be useful in broader interdisciplinary fields, such as tissue engineering and soft robotics. The capability to simultaneously measure stiffness, stress, and pressure within soft tubular structures makes this method particularly relevant for evaluating artificial blood vessels (ABVs) used in biomedical engineering (*55*). By providing real-time feedback on the mechanical properties and stress distribution within ABVs, our approach could aid in optimizing their design and ensuring functional stability under physiological conditions. In addition, the method offers a practical solution for monitoring soft robotic components, such as flexible tubing systems exposed to dynamic pressure environments (*56*, *57*). Given the susceptibility of soft materials to environmental factors such as temperature and pH, the capability to synchronously measure several key mechanical parameters in situ in a real-time manner could substantially advance their diverse applications.

## MATERIALS AND METHODS

### Study design

We recruited 44 normotensive volunteers and 8 hypertensive patients. Participants with systolic blood pressure (SBP) over 140 mmHg were grouped into HT. Other participants were divided into two groups, including young group (*n* = 30, 20 ± 2 years old) and older group (*n* = 14, 51 ± 8 years old). The older group has similar age range to the HT group (*n* = 8, 55 ± 5 years old). Demographic information of the participants is listed in table S1. All the participants signed an informed consent form before the experiments. Participants were asked to sit quietly for 10 min before the test in a sitting position.

At the beginning and end of experiments, brachial blood pressure was measured with an electronic sphygmomanometer (Upper Arm Blood Pressure Monitor HEM-7200, Omron, Japan). The average value of the two brachial blood pressure measurements was used as the calibration value for blood pressure measurement at the CCA (see details in the “Calibration of the carotid blood pressure” section). Arterial ultrasound elastography was conducted with an ultrasound system capable of generating programmed ARF and performing ultrafast imaging (Vantage 64LE System, Verasonics, USA). In the measurement, a L9-4 (central frequency, 7 MHz; element number, 124) linear array transducer (Jiarui Electronics, China) was used. Participants sat upright with a forward gaze during imaging. The probe was placed along the long-axis section of the right CCA with ultrasound gel between the probe and the skin to avoid the compression of the CCA. An ECG signal was recorded (ADInstruments, New Zealand) to trigger the ultrasound measurements, initiating 0.6 s after the QRS peak. After the elastography, a commercial ultrasound imaging system (Clover 60, Wisonic, China) equipped with a L15-4 (central frequency, 9.5 MHz; element number, 124) linear array transducer was used to measure the wall thickness precisely. The ultrasound probe was placed at the same position of the CCA, and the arterial walls in long-axis view were imaged. Afterward, a pressure tonometer system (SPT-301, Millar Instruments, USA) relying on the principle of applanation tonometry (*41*, *58*) was used to measure the blood pressure waveform of the CCA. The ECG signal was recorded simultaneously. The protocol was approved by the institutional review board (IRB) at Tsinghua University (approved IRB study number: 20210039).

### Imaging protocol

The ARF was imposed on the proximal artery (see Fig. 1B), and the guided wave propagating downstream was measured in this study. We defined a coordinate system *x*-*z* in the imaging plane, where *x* and *z* denote vertical and lateral direction, respectively. The origin (*z* = 0) is located at the focus of ARF. The ARF was generated by the focused ultrasound beams (voltage, ~10 V). A single ARF (i.e., one push) lasted ~43 μs (300 cycles). Ten focused ARFs were imposed successively along the depth from ~1 mm above the anterior wall to ~1 mm below the posterior wall (F-number, ~1.5), effectively enhancing the signal-to-noise ratio (SNR) of the guided waves (note S12). This sequential push (multiple ARFs) took in total ~0.5 ms and induced elastic Cherenkov effect (*59*) in arterial walls. The ultrasound system rested a duration of ~0.3 ms after excitation due to the voltage conversion of the system. Then, the plane wave imaging started at a pulse repetition frequency of 10 kHz (sampling period, 0.1 ms). The transducer transmitted and received unfocused ultrasound beams each time to reconstruct one frame. We designed an acquisition of 40 frames for plane wave imaging (4 ms). In-phase/quadrature (*I*/*Q*) data at both anterior and posterior walls were acquired. Although the guided wave was measured preferentially on the anterior wall in this study, signals on the posterior wall were shown to be vital for older and hypertensive volunteers. The tissue particle velocity $v_{x}\left(x,z,t\right)$ was estimated offline using the two-dimensional cross-correlator (*60*) with a kernel size of 5 × 2 (0.275 mm in *z* and 0.2 ms in *t*). A spatial filter (mean filter) with a kernel size of 4 × 4 (0.22 mm in *z* and 0.44 mm in *x*) was used to smooth particle velocity field. This single measurement (guided waves excitation and detection) took ~5 ms. We repeated the measurement described above for 36 times to continuously measure arterial biomechanics through cardiac cycles; the system rested 65 ms between two adjacent measurements. The 36 measurements totally took ~2.5 s, covering approximately three cardiac cycles.

The safety of the imaging method has been confirmed in our previous work (*20*). The mechanical index of the ARF is 0.73 [below the Food and Drug Administration (FDA) limit of 1.9 (*61*)]. The *I*_SPPA_ is 167 W/cm^2^ (below the FDA limit of 190 W/cm^2^). The *I*_SPTA_ is 119 mW/cm^2^ (below the FDA limit of 720 mW/cm^2^).

### Measurement of arterial geometry

To get arterial diameter waveform, the first frame of each single measurement was extracted, and, as a result, we obtained 36 frames in total. The arterial wall echoes at the same particle location from 36 frames were extracted and combined as an M-mode-like image. Then, we measured inner arterial diameter from the image (*62*). By correcting the value of diameter at the first line of M-mode image, the diameter waveform at the centerline of the wall was finally obtained (see details in fig. S8). The wall thickness was measured at the anterior wall from the B-mode image, as a whole of three layers (including intima, media, and adventitial layer). The wall thickness was identified according to literature (*63*). Limited by the spatial resolution of ultrasound (~0.15 mm), we only measure the wall thickness at end-diastole for further analysis.

### Measurement of axial group wave velocity

The axial wave velocity was measured by the time-to-peak (TTP) method (*64*). First, two windowing boundaries, i.e. the lower cut and upper cut, were added on the spatiotemporal map, aiming to remove circumferential and other noise signals. Then, a region with high SNR of axial wave was selected from the map. On this local map, the minimum peak of tissue particle velocity over time was recognized at each spatial location. Linear regression of the relationship between the peak velocity arrival time and the distance along the anterior wall yielded the slope as the axial wave velocity. The coefficient of determination *r*^2^ was used to evaluate the goodness of fit.

A comparison of the TTP method and another widely used method, the Radon transformation (*65*), was conducted (see fig. S9). Both methods show good results of estimation at diastolic state, while at the early systolic state, the TTP method provides a better fitting than Radon transformation. Therefore, the TTP method provides a robust estimation of axial wave velocity during cardiac cycles.

### Measurement of circumferential group wave velocity

To obtain the arrival time of circumferential guided waves, the particle velocity versus time at the origin of the length of the spatiotemporal map was drawn out, which theoretically coincided with the motivation location. The velocity curve was smoothed by moving average with a kernel size of 5, and the arrival time *t*_1_ was obtained by searching the first negative peak (generally in the range of ~1 to 2 ms). The circumferential wave velocity was then calculated by

$$c_{c}=\frac{\pi r_{c}}{t_{1}+t_{c}}$$ (4)

where $r_{c}$ is the radius of the artery. $t_{c}$ (=0.8 ms) is the compensation time. In the experiment, the anterior and posterior walls were excited sequentially, causing certain variations in the compensation time when calculating $c_{c}$ using signals from each wall. However, using a compensation time of 0.8 ms for both walls yields an estimation error of less than 6% (see note S13).

### Measurement of phase velocity of axial guided waves

To extract the dispersion curve that describes the variation of phase velocity with frequency, the spatiotemporal map was firstly preprocessed by applying windowing boundaries and removing mean value. Two windowing boundaries (*22*), including lower and upper cuts, are set to ensure that signals from axial guided wave are captured and circumferential guided wave are removed. Then, two-dimensional fast Fourier transformation was applied to the preprocessed map to get the *k*-space. By searching peaks at each frequency, we obtained a *f*-*k* curve (*f* denotes frequency and *k* denotes wave number), and the phase velocity was derived by $c_{p}=f/k$ (see details in note S9).

### Calibration of the carotid blood pressure

The carotid pressure waveform obtained by applanation tonometry was calibrated by the Kelly and Fitchet method (*5*, *41*, *58*). This calibration is based on the principle that the diastolic (DBP) and mean blood pressure (MBP) is constant through the large artery tree. Brachial DBP and systolic blood pressure (SBP) were acquired by a sphygmomanometer. The MBP at the brachial artery is calculated by MBP = SBP / 3 + 2 × DBP / 3 (*48*). The MBP at the CCA is the time-averaged value.

On the basis of the linear relationship between the square of circumferential group wave velocity and the blood pressure, the wave velocity can also be used to measure the carotid blood pressure. Blood pressure calibration using a sphygmomanometer is also required, following the same principle as the tonometry method. The blood pressure can be calculated using the circumferential group velocities as follows

$$P_{i}=\left({c_{c,i}}^{2}-{c_{c,\text{min}}}^{2}\right)\frac{\left(\text{SBP}-\text{DBP}\right)/3}{\left(\sum_{j=1}^{m}{c_{c,j}}^{2}\right)/m-{c_{c,\text{min}}}^{2}}+\text{DBP}$$ (5)

where $P_{i}$ and $c_{c,i}$ (*i* = 1, 2,…, *m*) denotes the blood pressure at the CCA and circumferential group wave velocity, respectively. *m* (=36 in this study) denotes the total number of data points. $c_{c,\text{min}}$ denotes the minimum velocity among the data points. Figure S10 demonstrates the calibration process.

### Measurement of circumferential stress of arteries

By measuring blood pressure *P*, arterial radius *r*_c_, and wall thickness *h*, the circumferential stress of the arteries can be determined using the Young-Laplace equation (*36*)

$$\sigma_{c}=\frac{{\text{Pr}}_{c}}{h}$$ (6)

### Phantom preparation and experiments

To verify the role of material viscoelasticity in the generation and measurement of circumferential guided waves in the longitudinal view, a homogeneous elastic polyvinyl alcohol cryogel (PVA) tube was fabricated and tested. The PVA solution consisted of (by weight) 85% distilled water, 14% PVA, and 1% Sigmacell cellulose. The PVA and cellulose were mixed into 85°C water and stirred until fully dissolved. Then, the PVA solution was poured into a tube mold and underwent solidification and polymerization by three freezing-thawing cycles. A freeze-thaw cycle lasted 48 hours, with −20°C freezing and 20°C thawing. The inner diameter and wall thickness of the tube phantom were 6.5 and 1.5 mm, respectively.

After the tube phantom was prepared, it was immersed in water and driven by the designed ARFs. The imaging settings were basically consistent with those of in vivo experiments, except for the following modifications: A single push-rest-imaging sequence was applied to the phantom. The durations of the ARFs and plane wave imaging were 0.5 and 7 ms, respectively. The voltage of the focused ultrasound beams was increased to 20 V to enhance SNR. Ultrasound measurements were repeated three times. To characterize the elastic properties of the phantom, quasi-static tensile tests (ElectroForce 3200, TA Instruments, USA) were also performed on the phantom samples (see note S2).

### Ex vivo experiments

To verify the ultrasound method of measuring axial stress and stiffness, an ex vivo experiment on porcine arteries was performed considering their mechanical similarity to human arteries (*66*). A segment of porcine thoracic aorta was obtained from a freshly slaughtered animal within 12 hours. Before the ultrasound experiment, the aorta was cut into a length of ~19 cm. The wall thickness of the aorta was ~3.5 mm, and the inner radius of the aorta was ~6.5 mm. Then, the aorta was cannulated at both ends and underwent different longitudinal prestretches, including $\lambda_{z}=$ 1 (stress-free), 1.23, and 1.31. Ultrasound measurements were repeated three times at each stretching state. A single push-rest-imaging sequence was applied to the sample. The durations of the ARFs and plane wave imaging were 0.5 and 4 ms, respectively. The voltage of the focused ultrasound beams was set to 20 V.

Tensile tests were conducted on the aorta sample with ElectroForce 3200 (TA Instruments, USA) subsequent to the ultrasound experiment to offer a comparative measurement of arterial stress and stiffness. The aorta sample was longitudinally dissected along the posterior side. Longitudinal and circumferential strips were cut from the sample, with dimensions of ~15 mm by 45 mm. The thickness of the strips was measured using a caliper at three different locations, and the average value was used to calculate the cross-sectional area. The uniaxial tensile tests were conducted on the longitudinal and circumferential strips, respectively. The grip distance was adjusted to position the strip in a load-free state without any sagging, and the corresponding strain and force were set to zero. The sample was stretched at a loading rate of 0.1 mm/s until the maximum displacement (~10 mm) was reached. After that, the displacement was held for 10 s. Subsequently, the sample was unloaded at a rate of 0.2 mm/s back to its original length. Measurements were repeated three times for each strip (see details in note S10).

### Finite element analysis

FEA was performed using Abaqus/CAE 6.14 (Dassault Systemes, USA). To simulate nonlinear viscoelasticity of arteries, we adopted the Gasser-Ogden-Holzapfel model (*67*) and quasi-linear Prony series model. The arterial wall was modeled as a homogeneous tube immersed in fluid. First, the tube underwent a quasi-static deformation caused by the inner blood pressure and axial prestretch. Then, a constant body force with a Gaussian distribution simulating the ARF was applied sequentially from the anterior to the posterior wall of the tube, which induced guided waves propagating along both the axial and circumferential directions (see fig. S11). Approximately 200,000 solid elements (C3D8R) were used to model the arterial wall, and about 300,000 acoustic elements (AC3D8) were applied to discrete the water. Convergence of the simulation was carefully examined by comparing the computational results with those given by a refined mesh and a smaller time step.

### Statistical analysis

Statistical analysis was preformed using MATLAB R2016b (MathWorks Inc., USA). All experimental data were expressed as means ± SD. Pearson’s correlation coefficient *r* was adopted to evaluate the correlation of the square of wave velocities with blood pressure. Bland-Altman analysis was used to compare two methods for measuring blood pressure: the elastography method (which uses circumferential wave velocities) and applanation tonometry. The unpaired *t* test was performed to compare bidirectional wave velocities, mechanical stiffnesses, and stresses. A *P* value of 0.05 was adopted to indicate statistical significance. Results with 0.01 ≤ *P* ≤ 0.05 were marked with one asterisk (*); those with 0.001 ≤ *P* < 0.01 were marked with two asterisks (**); and those with *P* < 0.001 were marked with three asterisks (***).

### Constitutive modeling of nonlinear viscoelastic arteries

The artery was modeled as a thin-walled tube subject to inner blood pressure *P* and axial prestretch. A cylindrical system was built for the artery, with θ , *r*, and *z* representing the circumferential, radial, and axial directions, respectively. $\lambda_{\theta }$ , $\lambda_{r}$ , and $\lambda_{z}$ denote the stretch ratio in circumferential, radial, and axial direction, respectively. The incompressible condition yields $\lambda_{\theta }\lambda_{r}\lambda_{z}=1$.

Fung’s quasi-linear viscoelastic model was adopted to describe arterial nonlinear viscoelasticity (*68*). Although more advanced arterial nonlinear viscoelastic models have been proposed (*69*, *70*), we found that the quasi-linear model is sufficient to capture the key features of the bidirectional guided waves observed in our experiments. The Cauchy stress σ is defined as

$$\sigma =-qI+\int_{0}^{t}\Xi \left(t-s\right)\cdot \frac{\text{∂}\sigma_{D}^{e}\left(s\right)}{\text{∂}s}ds$$ (7)

where *q* denotes the isochoric part of the stress and the second term denotes the deviatoric part. Ξ(t) is the relaxation function of the viscoelastic material. We used the first-order Prony series model to represent arterial viscoelasticity. Although fractional derivative model is more widely used for biological tissues (*71*), this model effectively captures the dispersion within the frequency bandwidth of 0.5 to 1.5 kHz in the ultrasound experiments (see details in note S14). Thus, we have

Ξ(t)=1−g[1−exp(−t/τ)] (8)

where *g* and τ denote the relaxation amplitude and characteristic relaxation time, respectively. $\sigma_{D}^{e}$ in Eq. 7 denotes the deviatoric part of elastic stress $\sigma^{e}$ . The elastic stress is related to the strain energy function *W* and deformation tensor **F** $=\text{diag}\left(\lambda_{\theta },\lambda_{r},\lambda_{z}\right)$ , according to $\sigma^{e}=\left(\text{∂}W/\text{∂}F\right)F^{T}$*.* We adopted the Gasser-Ogden-Holzapfel model to describe arterial passive hyperelasticity (*67*) and the active model used by Baek *et al.* (*72*) to describe active behavior of smooth muscles. The strain energy function is

$$\begin{array}{c} \begin{array}{c} W=\frac{\mu }{2}\left(I_{1}-3\right)+\frac{k_{1}}{2k_{2}}\sum_{i=4,6}\\ \left\{\;\text{exp}\left[k_{2}{\left[\kappa \left(I_{1}-3\right)+\left(1-3\kappa \right)\left(I_{i}-1\right)\right]}^{2}\right]-1\right\}+k_{\text{act}}\left[\lambda_{\theta }+\frac{1}{3}\frac{{\left(\lambda_{m}-\lambda_{\theta }\right)}^{3}}{{\left(\lambda_{m}-\lambda_{0}\right)}^{2}}\right] \end{array} \end{array}$$ (9)

where μ and $k_{1}$ are the initial shear modulus of elastin and collagen fibers, respectively. $k_{2}$ denotes the nonlinear stiffening. κ represents the fiber dispersion (between 0 and 1/3, whereas 1/3 corresponds to the isotropic material). The model consists of two symmetrically distributed fibers, with vectors $M_{1}={\left(\text{cos}\varphi ,0,\text{sin}\varphi \right)}^{T}$ and $M_{2}={\left(-\text{cos}\varphi ,0,\text{sin}\varphi \right)}^{T}$ , where φ denotes the angle between the fiber orientation and the circumferential direction. Invariants *I*_1_, *I*_4_, and *I*_6_ are defined as $I_{1}={\lambda_{\theta }}^{2}+{\lambda_{z}}^{2}+{\lambda_{\theta }}^{-2}{\lambda_{z}}^{-2}$ and $I_{4,6}={\lambda_{\theta }}^{2}{\text{cos}}^{2}\varphi +{\lambda_{z}}^{2}{\text{sin}}^{2}\varphi$ . The third term on the right-hand side of Eq. 9 is the active component, where $k_{\text{act}}$ denotes the degree of smooth muscle activation, $\lambda_{m}$ is the stretch corresponding to maximum contraction, and $\lambda_{0}$ is the stretch at which active force generation ceases. Quite a few advanced passive (*73*, *74*) and active constitutive models (*70*) for arteries have been proposed. Nevertheless, the current method for measuring arterial stiffness ( α ) and stress ( σ ) does not rely on the choice of a specific constitutive model, and the constitutive model given by Eq. 9 is used for demonstration. Interpreting the stiffness and stress, measured by this method, through advanced constitutive models has the potential to gain more information about arterial microstructure and active mechanical properties. However, this is beyond the scope of the present study.

### Guided axial and circumferential wave model for prestressed viscoelastic arteries

We imagine unrolling a cylindrical tube along its axial direction and unfolding it into a flat plate (see fig. S12). The dispersion relation for the guided waves propagating on this flat plate (Lamb wave) is given as follows. A Cartesian coordinate system was established on the plate, where *x*_r_, *x*_θ_, and *x_z_* correspond to the radial, circumferential, and axial directions of the artery, respectively. We consider that this viscoelastic prestressed flat plate is immersed in an inviscid fluid, with guided waves propagating along the axial direction. The excitation of ARF leads to the dominance of the antisymmetric mode of elastic waves (*19*). The dispersion relation is given by (see derivation in note S15)

$$\begin{array}{c} \begin{array}{c} \begin{array}{l} \left(1+{s_{2a}}^{2}\right)\cdot \left(-\rho \frac{\omega^{2}}{k^{2}}s_{1a}+C_{1a}s_{1a}-C_{2a}{s_{1a}}^{3}\right)\cdot \text{tanh}\left(s_{1a}\mathrm{kh}/2\right)\\ -\left(1+{s_{1a}}^{2}\right)\cdot \left(-\rho \frac{\omega^{2}}{k^{2}}s_{2a}+C_{1a}s_{2a}-C_{2a}{s_{2a}}^{3}\right)\cdot \text{tanh}\left(s_{2a}\mathrm{kh}/2\right)+\left({s_{1a}}^{2}-{s_{2a}}^{2}\right)\frac{\rho^{f}}{\xi }\frac{\omega^{2}}{k^{2}}=0 \end{array} \end{array} \end{array}$$ (10)

where *h* is the wall thickness. *s*_1a_ and *s*_2a_ are two roots solved by the quartic equation

$$\begin{array}{c} \left(\gamma +\frac{2.5\alpha_{a}+\gamma }{3}\Omega \right)s^{4}+\left[\rho \frac{\omega^{2}}{k^{2}}-8G\alpha_{a}+\frac{\gamma -2\alpha_{a}}{3}\Omega \right]\\ s^{2}+\alpha_{a}+\frac{2.5\alpha_{a}+\gamma }{3}\Omega -\rho \frac{\omega^{2}}{k^{2}}=0 \end{array}$$ (11)

and

$$C_{1a}=8G\alpha_{a}+\gamma +1.5\Omega \alpha_{a}$$ (12)

$$C_{2a}=\gamma +\frac{2.5\alpha_{a}+\gamma }{3}\Omega$$ (13)

$$\xi^{2}=1-\frac{\omega^{2}}{k^{2}}\frac{1}{{c_{f}}^{2}}$$ (14)

$$G=\left(1-\frac{g}{1+i\omega \tau }\right)/\left(1-g\right),\Omega =\left(\frac{g\omega^{2}\tau^{2}}{1+\omega^{2}\tau^{2}}+i\frac{g\omega \tau }{1+\omega^{2}\tau^{2}}\right)/\left(1-g\right)$$ (15)

where incremental parameters $\alpha_{a}$ and γ are defined as $\alpha_{a}=A_{0zrzr}$ and $\gamma =A_{0rzrz}=A_{0\text{rθrθ}}$ , respectively. $A_{0\text{jikl}}=\left({\text{∂}}^{2}W/\text{∂}F_{\mathrm{ip}}\text{∂}F_{\mathrm{lq}}\right)F_{\mathrm{jp}}F_{\mathrm{kq}}$ [*j*, *i*, *k*, *l*, *p*, *q* ∈ (*r*, θ, *z*)] is the fourth-order Eulerian elasticity tensor (see note S16 for explicit forms). *W* denotes the strain energy function of arteries. *g* and τ are viscous parameters. *i* in Eq. 15 denotes the imaginary unit. The density of the arterial wall is ρ = 1000 kg/m^3^. The bulk modulus of the blood is κ_p_ = 2.2 GPa. The density of the blood is ρ^f^ = 1000 kg/m^3^. The speed of sound in the blood is $c_{f}=\sqrt{\kappa_{p}/\rho^{f}}$ . ω ( =2πf ) denotes the angular frequency. *k* denotes the wave number.

The dispersion of axial guided waves in a tube differs from that of Lamb waves (*24*), which is caused by the curvature effect of the tube. We adopted a simplified model proposed by Li and Rose (*75*), which treats axial guided waves in a tube as Lamb waves in an unwrapped plate and imposes the periodic boundary condition on the circumferential direction of the plate. As a result, the phase velocity of axial guided waves, $c_{a}^{p}$ , can be calculated by Eq. 2, where *N* (=0, 1, 2,…) denotes the number of periodic waves in the circumferential direction. The excitation of ARF beam leads to multiple modes in low frequencies (e.g. <0.5 kHz) (*22*), and the ARF beam shape and location affects the dominant modes (*76*, *77*). In this study, by sequentially focusing the ARF from the anterior to the posterior wall, we found that the dominant mode of the axial guided wave in high frequencies (e.g. >0.6 kHz) corresponds to *N* = 2 (see details in note S6).

Both previous studies (*78*) and our FEA results (note S7) have verified that the dispersion curve of the circumferential guided wave can be well approximated by the antisymmetric mode of Lamb waves. The dispersion equation is (see derivation in note S15)

$$\begin{array}{c} \begin{array}{l} \left(1+{s_{2c}}^{2}\right)\cdot \left(-\rho \frac{\omega^{2}}{k^{2}}s_{1c}+C_{1c}s_{1c}-C_{2c}{s_{1c}}^{3}\right)\cdot \text{tanh}\left(s_{1c}\mathrm{kh}/2\right)\\ -\left(1+{s_{1c}}^{2}\right)\cdot \left(-\rho \frac{\omega^{2}}{k^{2}}s_{2c}+C_{1c}s_{2c}-C_{2c}{s_{2c}}^{3}\right)\cdot \text{tanh}\left(s_{2c}\mathrm{kh}/2\right)+\left({s_{1c}}^{2}-{s_{2c}}^{2}\right)\frac{\rho^{f}}{\xi }\frac{\omega^{2}}{k^{2}}=0 \end{array} \end{array}$$ (16)

where *s*_1c_ and *s*_2c_ are two roots solved by the quartic equation

$$\begin{array}{c} \left(\gamma +\frac{1.7\alpha_{c}+\gamma }{3}\Omega \right)s^{4}+\left[\rho \frac{\omega^{2}}{k^{2}}-8G\alpha_{c}+\left(\frac{1}{3}\gamma -0.1\alpha_{c}\right)\Omega \right]\\ s^{2}+\alpha_{c}+\frac{1.7\alpha_{c}+\gamma }{3}\Omega -\rho \frac{\omega^{2}}{k^{2}}=0 \end{array}$$ (17)

and

$$C_{1c}=8G\alpha_{c}+\gamma +0.7\Omega \alpha_{c}$$ (18)

$$C_{2c}=\gamma +\frac{1.7\alpha_{c}+\gamma }{3}\Omega$$ (19)

where incremental parameter $\alpha_{c}$ is defined as $\alpha_{c}=A_{0\theta \mathrm{r\theta r}}$. Equation 16 determines the relationship between ω and *k*, and the circumferential phase velocity can be calculated by $c_{c}^{p}=\omega /\text{Re}\left(k\right)$.

### Genetic algorithm–assisted inversion to infer mechanical parameters of arteries

To infer arterial axial stiffness and stress from axial guided waves in arteries, we proposed a genetic algorithm–aided inverse method. The dispersion curves of axial guided waves at diastole (minimal pressure) and systole (peak pressure) were marked as $\left(f_{i,d},c_{i,d}^{p}\right)$ and $\left(f_{i,s},c_{i,s}^{p}\right)$ , respectively (*i* = 1, 2,…*n*, where *n* denotes the amount of data points; the frequency range for diastolic data is 0.6 to 1.5 kHz, and that for systolic curve is 0.7 to 1.5 kHz; thus, *n* is approximately 45 and 40 for diastolic and systolic data, respectively). The arterial radius at diastolic and systolic states are $r_{c,d}$ and $r_{c,s}$ , respectively. According to Eq. 10, two elastic incremental parameters $\alpha_{a}$ and γ and two viscous parameters *g* and τ are to be optimized from the experimental dispersion data. Assuming that the viscous parameters *g* and τ remain relatively constant, while the incremental parameters $\alpha_{a}$ and γ change significantly between diastolic (with subscript “d”) and systolic states (with subscript “s”), there are a total of six unknown parameters to be optimized in the two states, including $\alpha_{a,d}$ , $\gamma_{d}$ , $\alpha_{a,s}$ , $\gamma_{s}$ , *g*, and τ. The optimization was achieved by the genetic algorithm (population size, 50; crossover fraction, 0.8; migration fraction, 0.2). Compared to traditional iterative algorithms (e.g., Levenberg-Marquardt), it has advantages in global optimization for multiparameter searches and does not rely on the gradient for its optimization direction. The best fit was obtained by minimizing the goodness-of-fit function (e.g. F<0.1 in practice)

$$F=\left(F_{d}+F_{s}\right)/2$$ (20)

where

$$\begin{array}{c} F_{d}=\sqrt{\frac{\sum_{i=1}^{n}{\left[{c_{i,d}^{p}}^{\left(\text{exp}\right)}-{c_{i}^{p}}^{\left(\text{theo}\right)}\left(\alpha_{a,d},\gamma_{d},g,\tau ;f_{i,d},r_{c},h\right)\right]}^{2}}{n}} \end{array}$$

$$\begin{array}{c} F_{s}=\sqrt{\frac{\sum_{i=1}^{m}{\left[{c_{i,s}^{p}}^{\left(\text{exp}\right)}-{c_{i}^{p}}^{\left(\text{theo}\right)}\left(\alpha_{a,s},\gamma_{s},g,\tau ;f_{i,s},r_{c},h\right)\right]}^{2}}{m}} \end{array}$$ (21)

where ${c_{i}^{p}}^{\left(\text{exp}\right)}$ and ${c_{i}^{p}}^{\left(\text{theo}\right)}$ denote experimental and theoretically predicted axial phase velocity (Eq. 2 and Eq. 10). The relationship between diastolic and systolic state provides constraints on material parameters, including $\alpha_{a,s}>\alpha_{a,d}$ and $\gamma_{s}>\gamma_{d}$ . Parameter spaces were set as $20\leq \alpha_{a}\leq 200\;\text{kPa}$ , $0<\gamma /\alpha_{a}<1$ , 0.3<g<0.95 , and $10^{-5}<\tau <10^{-4}\;s$ (see details in note S17). As a result, six parameters were obtained by using the genetic algorithm–aided inversion, i.e., $\left(\alpha_{a,d},\gamma_{d},\alpha_{a,s},\gamma_{s},g,\tau \right)$.

Using dispersion data from both diastole and systole improves the stability of the inversion parameters compared to using data from a single state. Numerical examples were used to assess the stability of the inversion method, demonstrating that the estimated parameters exhibit acceptable stability; for example, the inversion error of $\alpha_{a}$ is within 5% (see note S18). The inversion efficiency was also evaluated by central processing unit runtime, requiring ~6 min on a standard laptop (see details in note S18). This also indicates the potential of the method for practical applications.

### Relationship between arterial stress and incremental parameters

The general relationship between the stress and incremental parameters satisfies (*38*, *79*)

$$\sigma_{c}-\sigma_{r}=\alpha_{c}-\gamma$$ (22)

and

$$\sigma_{a}-\sigma_{r}=\alpha_{a}-\gamma$$ (23)

where $\sigma_{r}$ , $\sigma_{a}$ , and $\sigma_{c}$ represent the radial, axial, and circumferential stress, respectively. For arteries subjected to blood pressure and axial stretch, the radial stress is significantly smaller than both the axial and circumferential stress (*49*); hence, we get the following approximate relationship

$$\sigma_{c}=\alpha_{c}-\gamma$$ (24)

and

$$\sigma_{a}=\alpha_{a}-\gamma$$ (25)

## Supplementary Materials

### The PDF file includes:

Figs. S1 to S27 Notes S1 to S19 Tables S1 to S3 Legend for movie S1 References
