# Supplementary material for: Simultaneous imaging of bidirectional guided waves probes arterial mechanical anisotropy, blood pressure, and stress synchronously
Source: Sci Adv. 2025 Aug 6;11(32):eadv5660. doi: 10.1126/sciadv.adv5660 (PMC12327456; doi:10.1126/sciadv.adv5660)
Supplement: Supplementary file 1 — Figs. S1 to S27 Notes S1 to S19 Tables S1 to S3 Legend for movie S1 References [file sciadv.adv5660_sm.pdf]

Science Advances
AAAS

Supplementary Materials for

Simultaneous imaging of bidirectional guided waves probes arterial mechanical anisotropy, blood pressure, and stress synchronously

Yuxuan Jiang *et al.*

Corresponding author: Guo-Yang Li, lgy@pku.edu.cn; Xinyu Wang, wangxinyu@bjmu.edu.cn; Yanping Cao, caoyanping@tsinghua.edu.cn

Sci. Adv. **11**, eadv5660 (2025)
DOI: 10.1126/sciadv.adv5660

The PDF file includes:

Figs. S1 to S27
Notes S1 to S19
Tables S1 to S3
Legend for movie S1
References

Other Supplementary Material for this manuscript includes the following:

Movie S1

Supplementary Figures

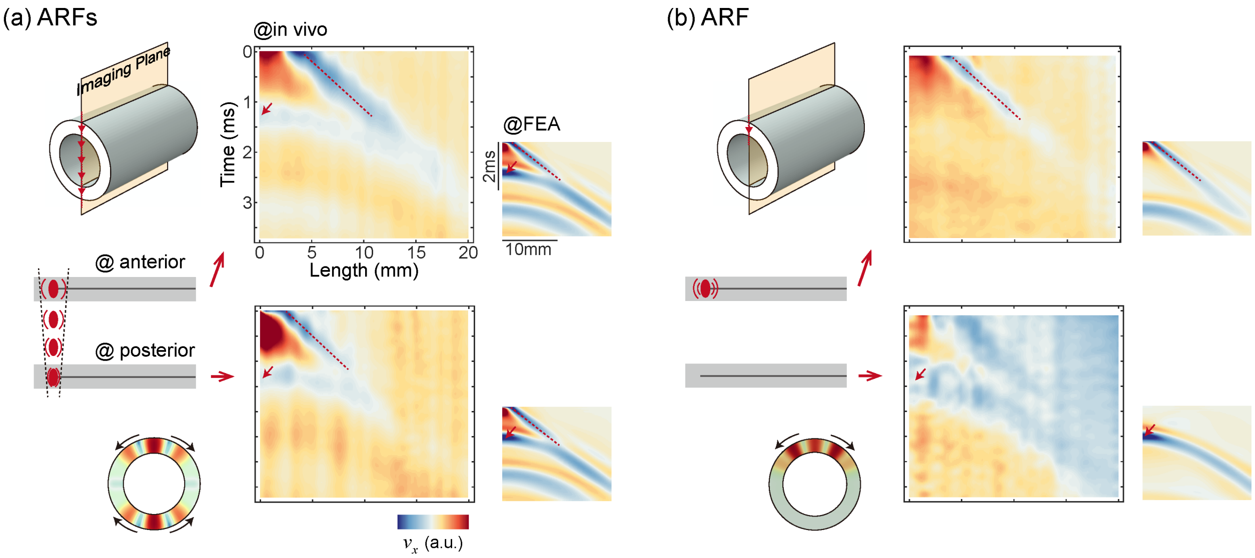

Fig. S1. An *in vivo* comparative experiment on a young volunteer under different excitation conditions. (a) Excitation with multiple ARFs on both anterior and posterior walls, and **(b)** a single ARF on the anterior wall. The *in vivo* particle velocity maps at end-diastole were extracted along both the anterior and posterior walls. Finite element analysis (FEA) were also performed, and the results are presented for comparison with the *in vivo* maps.

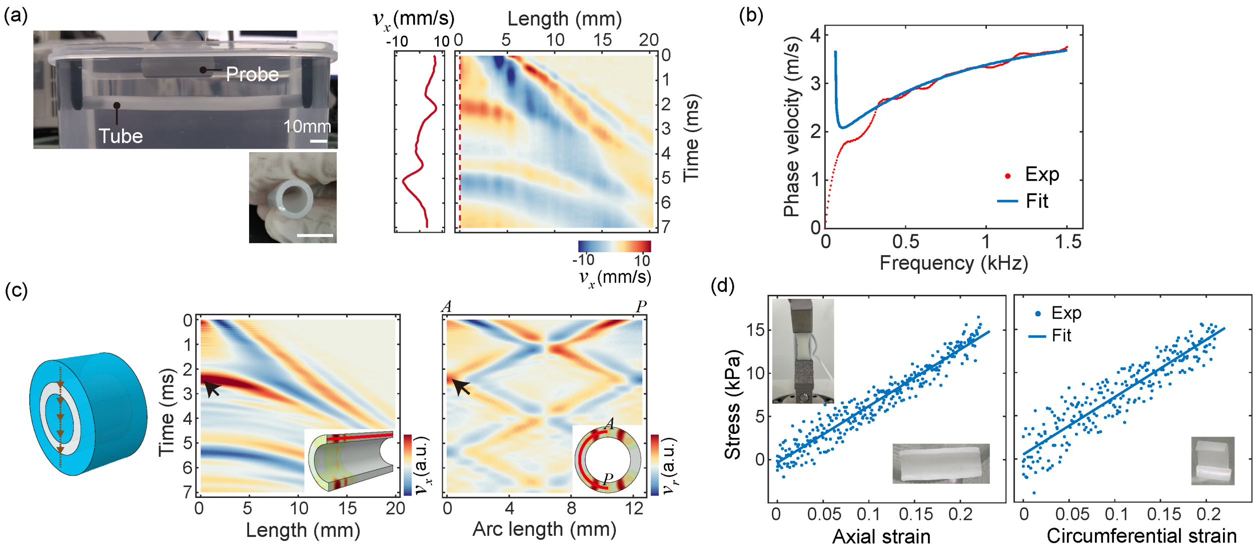

Fig. S2. Ultrasound elastography experiments on an artery phantom. (a) Experimental set-up (left) and the particle velocity map extracted along the anterior wall (right). **(b)** Curve fitting of experimental wave dispersion using the L(2,1) mode of axial guided waves. **(c)** Results of finite element simulation, including particle velocity maps extracted along the axial and circumferential paths. The geometry and modulus of the model are the same as those of the phantom: wall thickness 1.5 mm, middle radius 4 mm, shear modulus 25 kPa. **(d)** Uniaxial tensile tests on the artery phantom along axial (left) and circumferential directions (right), respectively.

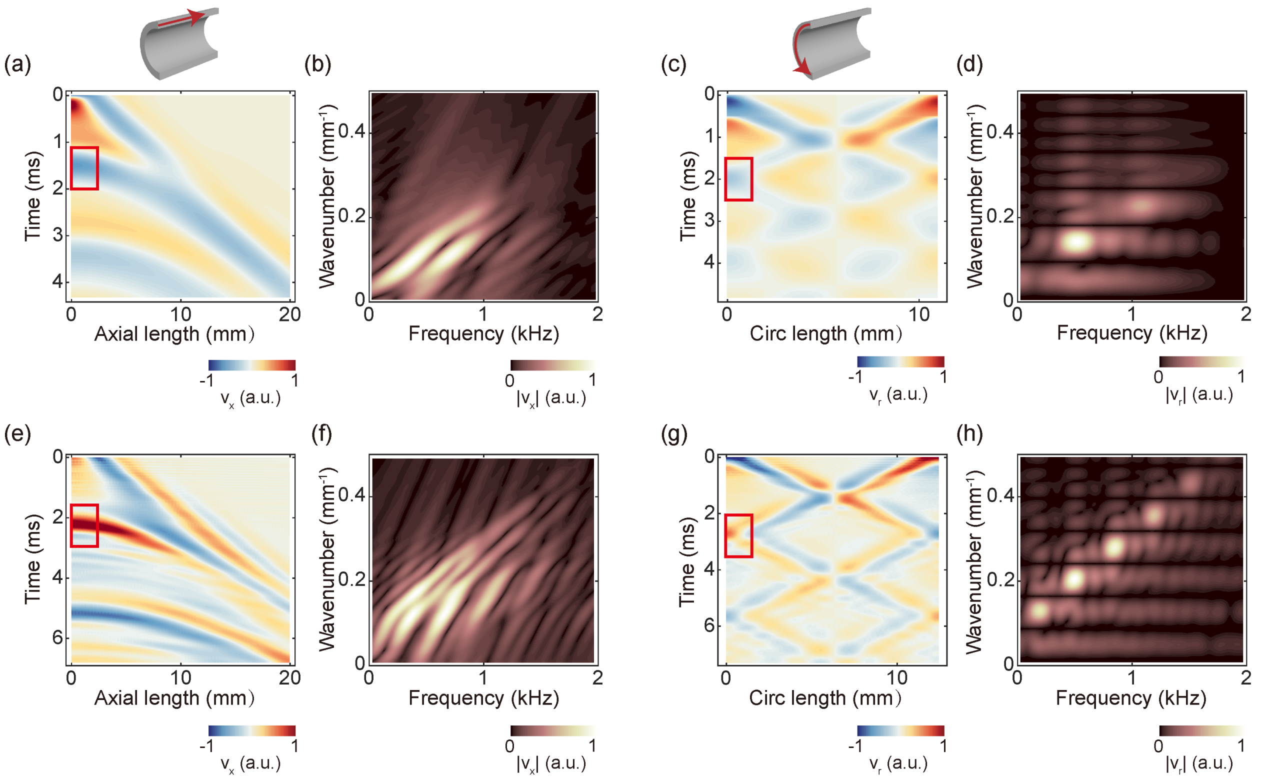

Fig. S3. Finite element results of the elastic and viscoelastic models. (a) – (d) Viscoelastic model mimicking the in vivo conditions. (e) – (h) Elastic model mimicking the phantom conditions. **(a)** Spatiotemporal velocity map extracted along the axial path of the viscoelastic model, and **(b)** corresponding frequency-wavenumber spectrum obtained by applying 2D FFT to the spatiotemporal map. **(c)** Spatiotemporal velocity map extracted along the circumferential path of the viscoelastic model, and **(d)** corresponding frequency-wavenumber spectrum. **(e)** Spatiotemporal velocity map extracted along the axial path of the elastic model, and **(f)** corresponding frequency-wavenumber spectrum. **(g)** Spatiotemporal velocity map extracted along the circumferential path of the elastic model, and **(h)** corresponding frequency-wavenumber spectrum. The red boxes in (a), (c), (e), and (g) represent the features of the circumferential guided waves.

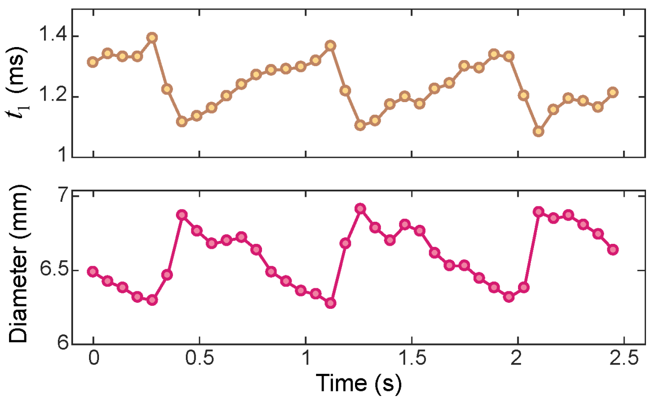

Fig. S4. Dynamic changes of arterial diameter and circumferential time t_1 in cardiac cycles.

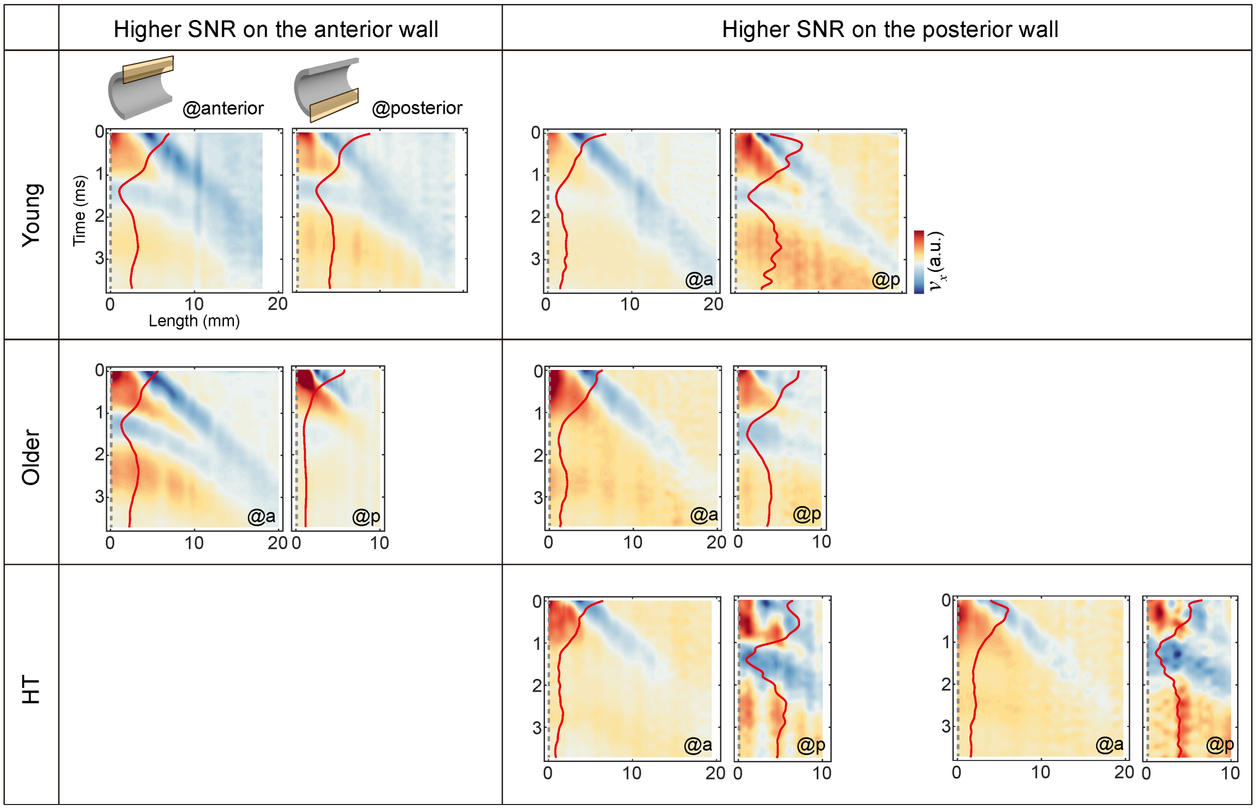

Fig. S5. Particle velocity maps extracted from both the anterior and posterior walls at end-diastole for the young, older and hypertensive participant groups. In each participant group, representative results from two individuals are presented. These *in vivo* results are divided into two categories based on the signal-to-noise ratio (SNR) of peaks at time t_1 on the anterior and posterior walls: one with a higher SNR of time t_1 on the anterior wall, and the other with a higher SNR of time t_1 on the posterior wall. In most young participants, the SNR on the anterior wall is better (or at least comparable) to that on the posterior wall, with only a few participants showing lower SNR on the anterior wall (4 out of 30). In contrast, most older participants (11 out of 14) and all hypertensive participants (8 out of 8) exhibit higher SNR on the posterior wall than on the anterior wall.

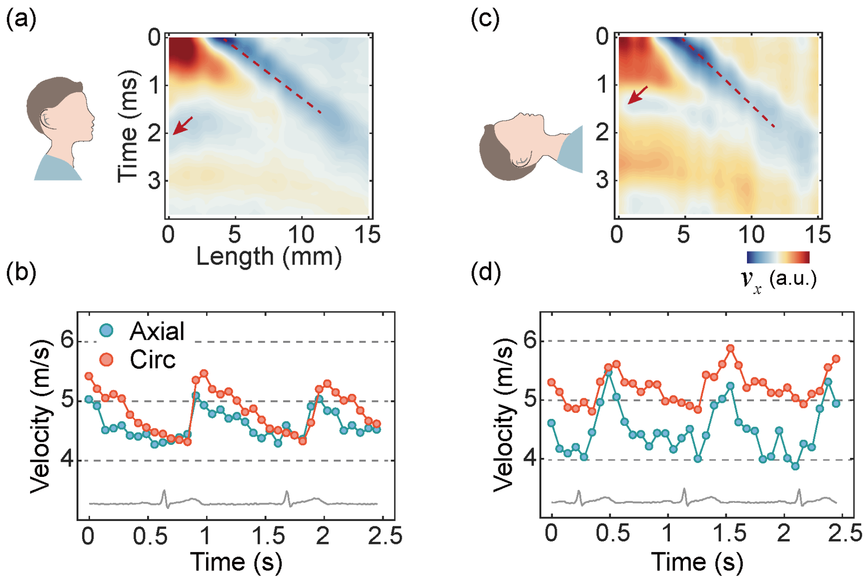

Fig. S6. An *in vivo* comparative experiment on a young volunteer in two postures, including sitting and supine postures. (a) Particle velocity maps at end-diastole for the sitting posture. **(b)** Bidirectional group velocities in cardiac cycles for the sitting posture. **(c)** Particle velocity maps at end-diastole for the supine posture. **(d)** Bidirectional group velocities in cardiac cycles for the supine posture. The synchronous ECG signal is also plotted.

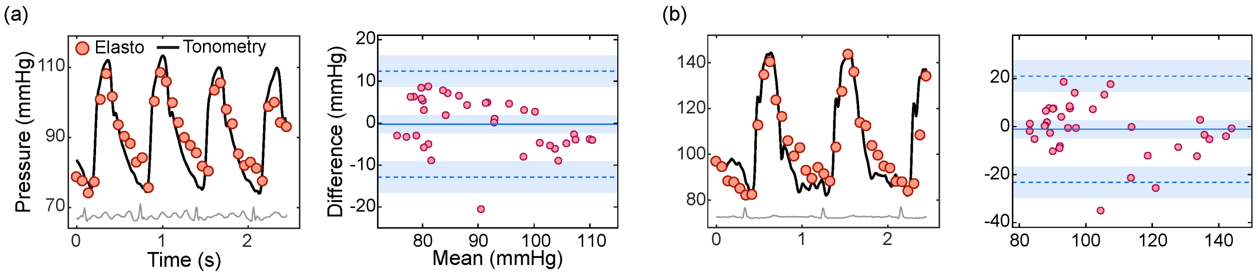

Fig. S7. Bland-Altman plot of the two blood pressure measurement methods, i.e., ultrasound elastography and applanation tonometry. (a) B-A plot of an older normotensive participant (56 years old, male), and **(b)** an older hypertensive participant (61 years old, female). The biases are: (a) -0.2 mmHg in average, with 95% limits of agreement (LoA) of -12.9 to 12.5 mmHg; (b) -1.1 mmHg with LoA of -23.2 to 21.0 mmHg.

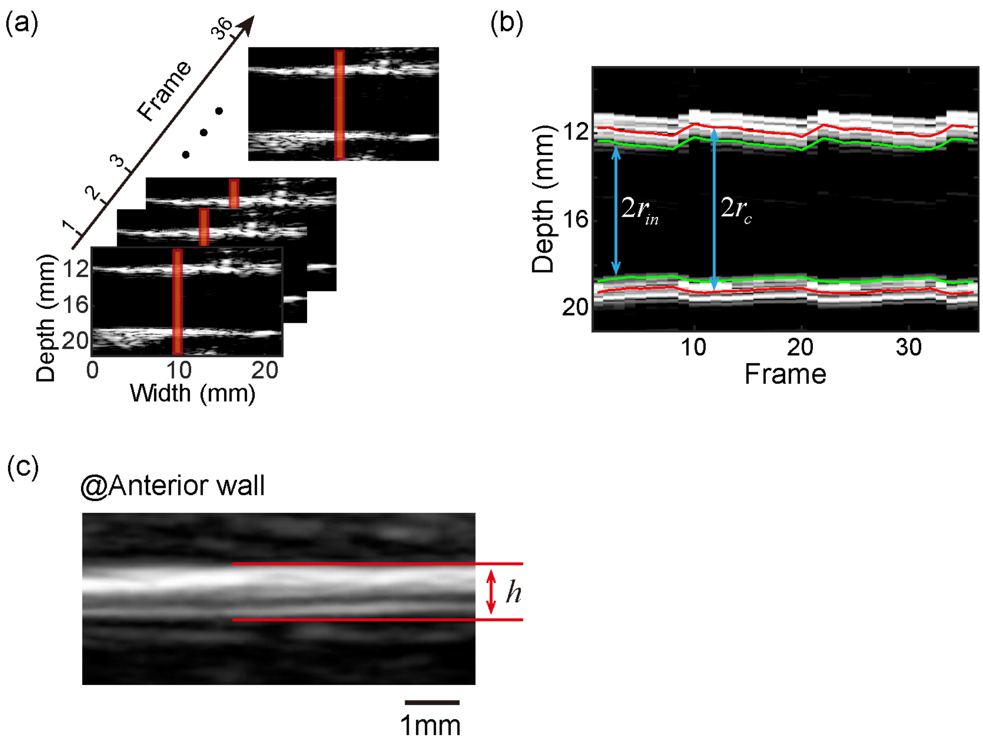

Fig. S8. Measurement of arterial diameter and wall thickness. (a) Extraction of M-mode image. In 36 repeated measurements, the first frame of the B-mode image from each measurement was extracted. For each frame, the A-line at the center position (width = 10 mm) was selected. These 36 A-lines were then arranged in sequence to construct the M-mode image. **(b)** Measurement of the arterial diameter from the M-mode image. The inner radius r_{in} is firstly detected; then the middle radius can be obtained using $r_c = r_{in} + h/2$. **(c)** The wall thickness h is measured at the anterior wall at end-diastole.

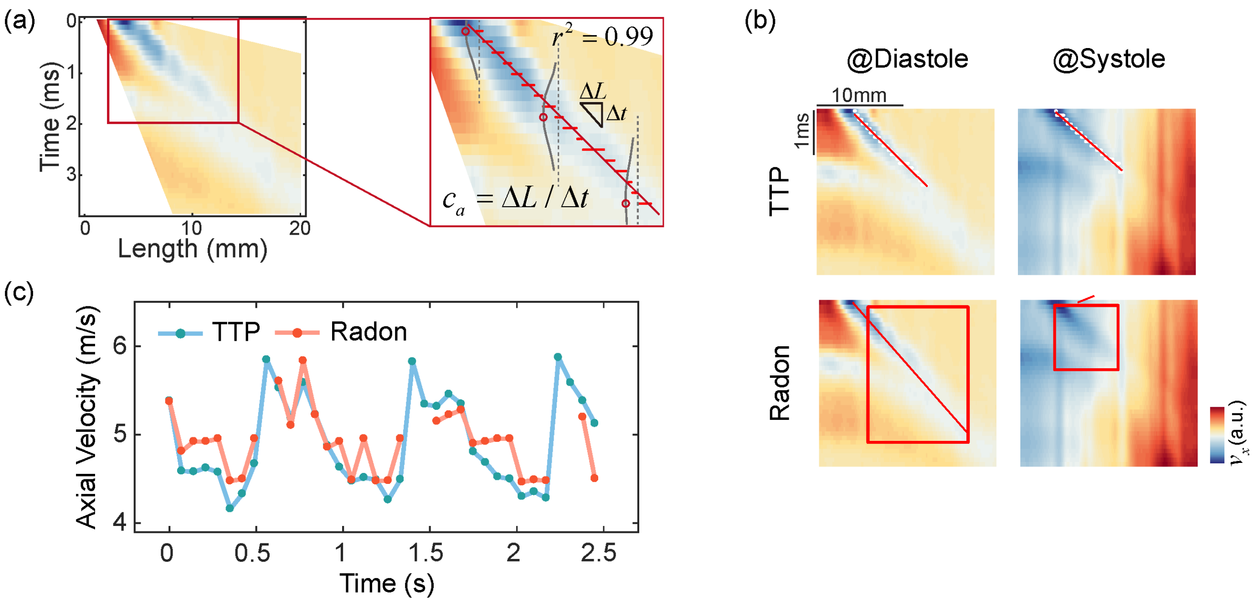

Fig. S9. Measurement of axial group wave velocities. (a) The time-to-peak (TTP) method to measure axial wave velocities. **(b)** *In vivo* measurements using the TTP method and the Radon transformation method at diastole and systole. The Radon transformation method fails to fit the axial wave velocity at systole. **(c)** Comparison of the axial wave velocities measured by the TTP method and Radon transformation method. The wave velocity curve using Radon transformation method exhibits some discontinuities. In contrast, the TTP method is more robust and leads to a continuous wave velocity curve in cardiac cycles.

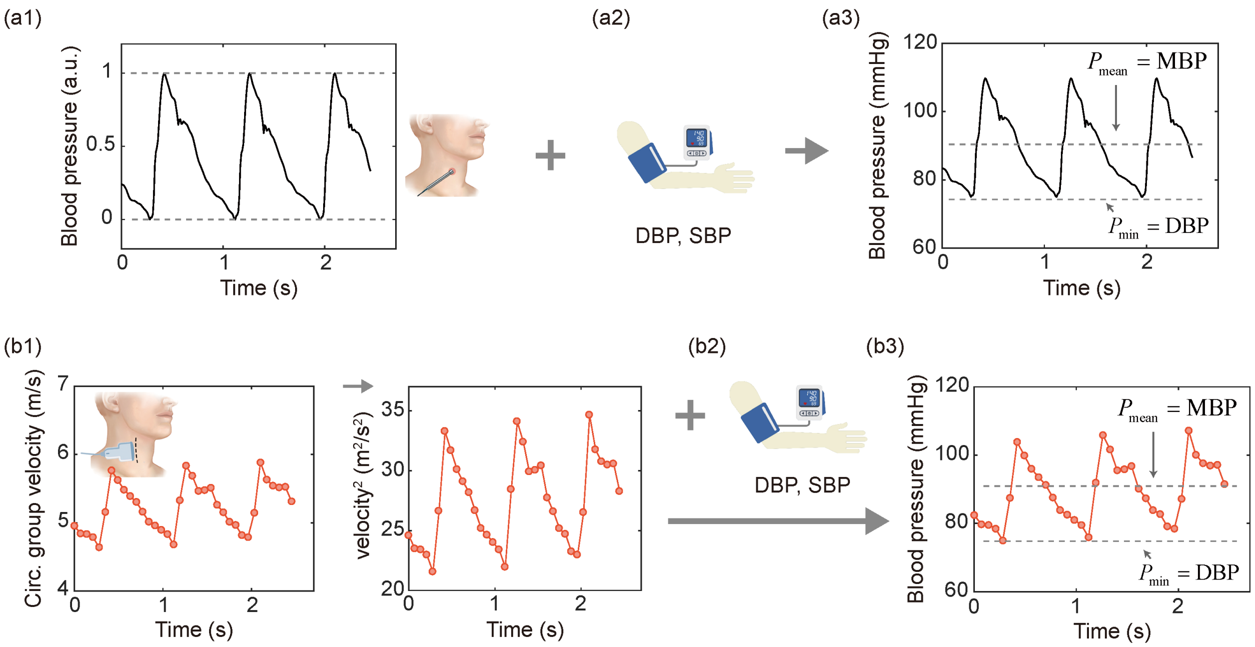

Fig. S10. Calibration process for the blood pressure measurement methods. (a1) – (a3) Applanation tonometry method. (b1) – (b3) Ultrasound elastography method. **(a1)** Normalized blood pressure waveform measured by a tonometer at the common carotid artery. **(a2)** Brachial diastolic (DBP) and systolic blood pressure (SBP) measured by a digital sphygmomanometer. **(a3)** Linear mapping of carotid blood pressure from the normalized tonometry data. The principle of mapping is: the diastolic blood pressure $P_{\min}$ and the mean blood pressure P_{mean} are equal to those measured at the brachial artery, i.e., $P_{\min}$ = DBP, and P_{mean} = MBP. $\text{MBP} = \frac{1}{3}\text{SBP} + \frac{2}{3}\text{DBP}$. $P_{mean} = \frac{1}{T}\int_0^T P(t)\mathrm{d}t$. **(b1)** Circumferential group wave velocities (left), and their squared values (right). **(b2)** Brachial diastolic (DBP) and systolic blood pressure (SBP) measured by a sphygmomanometer. **(b3)** Linear mapping of carotid blood pressure from the squared circumferential group wave velocities. The principle of mapping is the same as that in tonometry.

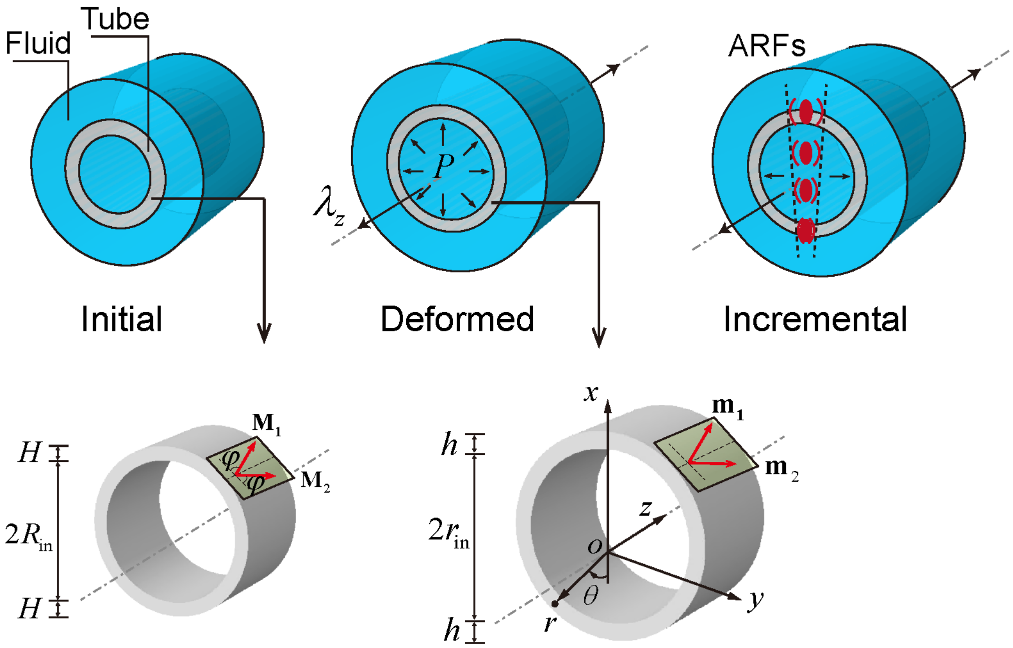

Fig. S11. Finite element model (FEM) to simulate arterial bidirectional guided waves. A single-layer tube immersed in fluid was built to simulate arterial conditions. Three mechanical configurations were included: the initial state (stress-free), the deformed state (artery subjected to blood pressure P and axial stretch λ_z), and the incremental state (elastic waves excited by ARFs). The wall thickness and inner radius of the artery in the initial state are H and R_{in}, respectively. The wall thickness and inner radius in the deformed state are h and r_{in}, respectively. Gasser-Ogden-Holzapfel hyperelastic model and Prony-series viscoelastic model were used in the FEM. $\mathbf{M_1}$ and $\mathbf{M_2}$ represents two symmetric fiber orientations in the initial state, and they change to $\mathbf{m_1}$ and $\mathbf{m_2}$ in the deformed state.

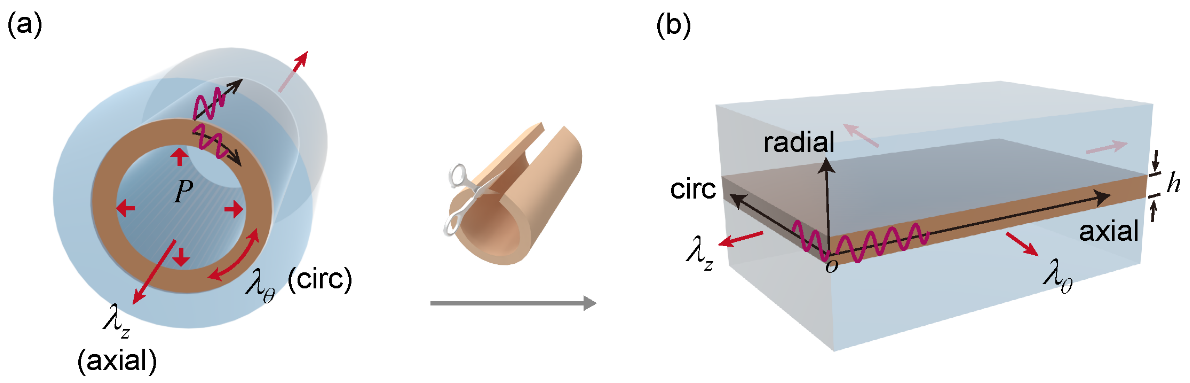

Fig. S12. Mechanical modeling of the arterial bidirectional guided waves. (a) The artery is modeled as a single-layer cylindrical tube immersed in fluid. The artery is subjected to blood pressure (P) and axial stretch, where λ_θ and λ_z denote circumferential and axial stretch ratio, respectively. **(b)** The tube is cut along the axial direction and unfolded into a flat configuration, with fluid on both the upper and lower sides. A Cartesian coordinate system is established on the plate, with the three directions referred to as axial (x_z), circumferential (x_θ), and radial directions (x_r). The plate is subject to biaxial stretching (stretch ratio λ_θ and λ_z), and elastic waves propagate on this pre-stretched plate. The wall thickness is denoted as h.

Supplementary Note S1. In vivo comparative experiment between single and multiple ARF excitations

A comparative experiment was conducted on a young healthy volunteer (27 years old, male) to verify the mechanical meaning of the time phase t_1. As depicted in Fig. S1a, when utilizing multiple ARFs, guided waves were generated on both the anterior and posterior walls. Notably, the velocity maps extracted from both the anterior and posterior walls exhibit the characteristics associated with the time phase t_1. In contrast, as shown in Fig. S1b, when applying a single ARF on the anterior wall, the time phase t_1 could not be detected on the map extracted from the anterior wall, but was identifiable on the posterior wall. This is mainly because no guided wave is excited from the posterior wall at the initial moment, so no wave signal reaches the anterior wall at time t_1. On the other hand, the guided wave excited from the anterior wall at the beginning reaches the posterior wall at time t_1, making it detectable there.

We also performed finite element simulations to verify our assumption. In the simulation, we tested both simultaneous excitation of two walls, and excitation of the anterior wall only, and obtained the corresponding velocity maps in Fig. S1. The FEA results are consistent with the *in vivo* results. Therefore, we conclude that the time phase t_1 observed at the anterior/posterior wall denotes the arrival time of the circumferential guided wave originating from the opposite wall.

Supplementary Note S2. A phantom experiment to study the effect of material viscoelasticity on circumferential guided wave signals

Figure S2a shows the experiment setup of the phantom experiment. The tube phantom was immersed in water with both ends fixed. The ultrasound experiment was performed along the longitudinal direction of the tube. On the right of Fig. S2a, it presents the particle velocity map extracted along the longitudinal path on the anterior wall. Different from in vivo results, no minimum peak can be observed at time t_1 at $z = 0$; instead, a positive peak appears ($t \approx 2$ ms). Fig. S2c presents the results of finite element simulation with pure elastic material. The velocity map extracted along the longitudinal path was similar to that of the phantom experiment. The velocity map extracted along the circumferential path provides additional clarity, showing that the positive peak at $z = 0$ and $t \approx 2$ ms (indicated by an arrow) represents the arrival of high-frequency component of the circumferential guided waves. However, this peak cannot be directly used to calculate the circumferential group wave velocity.

By applying 2D-FFT to the spatiotemporal velocity map, the dispersion curve was obtained in Fig. S2b. Similar to the *in vivo* experiments, the dominant wave mode excited by ARFs was L(2,1) mode. By fitting the experimental dispersion to the L(2, 1) mode of axial guided waves (a linear isotropic elastic tube immersed in fluid (*80*)), the Young's modulus of the phantom artery was fitted to be 70.4 kPa. Uniaxial tension tests were then conducted along both the axial and circumferential directions of the tube samples. As shown in Fig. S2d, the Young's moduli were fitted as 65.5 kPa along the axial direction, and 66.4 kPa along the circumferential direction. The similar values of Young's moduli in two directions indicate the isotropy of the phantom. And the similar values of Young's moduli obtained from the ultrasound experiment and the tensile test indicates that the phantom exhibits good elasticity.

The difference between the particle velocity maps obtained from the in vivo experiment and the phantom experiment mainly stems from the distinct viscoelastic properties of the artery and

the phantom. Figure S3 compares the results of FE simulations of elastic and viscoelastic materials. The results for elastic materials exhibit a broader frequency spectrum (Fig. S3f and h), whereas those for viscoelastic materials have an effective spectrum limited to below ~1.5 kHz (Fig. S3b and d). This divergence originates from frequency-dependent attenuation in viscoelastic materials, which intensifies with increasing frequency. Consequently, spatiotemporal velocity maps of elastic materials display broadband wave components (Fig. S3e, g), whereas viscoelastic materials suppress high-frequency content, leaving low-frequency elastic waves dominant (Fig. S3a, c). Compared to arteries, the circumferential guided waves in the phantom material exhibit stronger dispersion, resulting in a broadband wave components traveling from the posterior to the anterior wall. This makes it difficult to distinguish the arrival time t_1 of the low-frequency wave packet directly in the longitudinal view.

Supplementary Note S3. Estimation of acoustic radiation force at the anterior and posterior walls

The acoustic radiation force f is proportional to the acoustic intensity I, and the acoustic intensity is proportional to the square of the acoustic pressure P (*61*). Therefore, the ratio of the acoustic force between the anterior and posterior walls can be expressed as follows:

$$\frac{f_1}{f_2} = \left(\frac{P_1}{P_2}\right)^2 \tag{S1}$$

where f_1 and f_2 denote the acoustic radiation force at the anterior and posterior wall, respectively. P_1 and P_2 denote the acoustic pressure at the anterior and posterior wall, respectively. The acoustic pressure attenuates exponentially with depth in soft tissues, i.e.

$$P(z) = P_0 10^{-\alpha f_c z/20} \tag{S2}$$

where α denotes the acoustic attenuation. f_c (= 7 MHz) denotes center frequency of the ultrasound transducer. z denotes the distance from the ultrasound probe to the measured point. P_0 denotes the initial acoustic pressure emitted from the ultrasound probe. Inserting Eq. (S2) into Eq. (S1) yields

$$\frac{f_1}{f_2} = 10^{\alpha f_c \Delta z/10} \tag{S3}$$

where Δz denotes the diameter of the artery.

For healthy volunteers, the acoustic attenuation of arteries is approximately 0.3 dB/cm/MHz (*61*). Δz is approximately 0.7 cm. Using Eq. (S3) we get $f_1/f_2 \sim 1.4$. For hypertensive volunteers, the acoustic attenuation of arteries may even exceed 1 dB/cm/MHz (*81*). Δz is approximately 0.9 cm. Using Eq. (S3) yields $f_1/f_2 \sim 4$.

Supplementary Note S4. In vivo comparative experiment between sitting and supine postures

A comparative experiment was performed on a young healthy volunteer (24 years old, male) in two different postures—sitting and supine postures—with the aim of introducing a transient alteration in blood pressure. The volunteer was firstly measured in a sitting posture, followed by measurements in a supine posture. For each posture, ultrasound elastography measurement was carried out on the volunteer's right common carotid artery. Figure S6a and c show the spatiotemporal velocity maps at the end-diastole for the sitting and supine postures. The time t_1 (indicated by arrows in Fig. S6a and c) is greater in the sitting posture than in the supine posture, suggesting changes in the circumferential group wave velocity. Figure S6b and d illustrate the variation in bidirectional group velocities during cardiac cycles for the two postures. In line with the observations from the maps, the circumferential group velocity in the supine posture is approximately 8% higher than that in the sitting posture (supine: 5.2 ± 0.3 m/s; sit: 4.8 ± 0.3 m/s, where mean ± SD represents the group velocities over cardiac cycles). Conversely, the axial group velocity remains relatively stable (supine: 4.5 ± 0.4 m/s, sit: 4.6 ± 0.2 m/s). Due to hydrostatic pressure differences, the carotid artery pressure in the sitting posture is about 10 - 20 mmHg lower than that in the supine posture (*40*). Assuming that the mean blood pressure at the carotid arteries rises from 90 mmHg in the sitting position to 105 mmHg in the supine position, the relative increase in blood pressure (approximately 16%) is twice that of the circumferential group wave velocity (approximately 8%). Given the linear relationship between pressure and the square of the group wave velocity, the change in the circumferential group velocity is consistent with the variation in blood pressure. These results further prove that the circumferential group velocity can be employed to measure blood pressure.

Supplementary Note S5. Validity of modeling arterial tissues as a homogeneous medium for guided wave analysis

The artery is composed of three layers, and the mechanical properties of each layer are different. In this study, we model the artery tissues as a one-layer model. The validity of this approximation is discussed as follows. For healthy people, the artery is mechanically equivalent to a two-layer structure, including intima-media layer and adventitia layer. For the A0 mode of the Lamb waves (which is the primary wave mode generated in ultrasound elastography), there is a critical frequency below which the phase velocity predicted by the two-layer model closely aligns with that predicted by the one-layer model. As shown in Fig. S13, in the case of $\mu_1/\mu_2 = 1/3$ (considering that the adventitia is stiffer than the media in the physiological range) and wall thickness of 1 mm, this critical frequency f_c is ~ 4.5 kHz, below which the relative difference between the two curves is less than 5%. This suggests that using the one-layer assumption to analyze guided wave dispersion within frequency range below 2 kHz (which is generally the frequency bandwidth in the ultrasound elastography) is reasonable. The similarity of the two curves can be explained by the long wavelength of elastic waves at low frequencies. The typical frequency and phase velocity of the elastic waves in ultrasound elastography are 1 kHz and 6 m/s, respectively, resulting in a wavelength of ~ 6 mm, which is significantly larger than the wall thickness of the carotid artery (~1 mm). In this case, the elastic wave is primarily governed by the average stiffness of multiple layers of arteries.

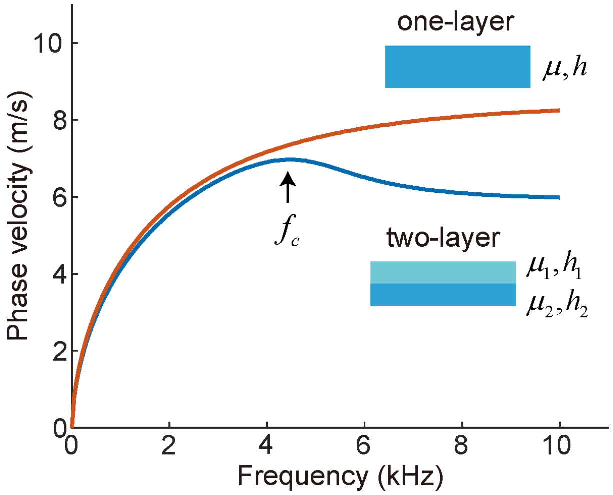

Fig. S13. Comparison of wave dispersion predicted by the one-layer and two-layer Lamb wave models. The layers are immersed in water. In the two-layer model, the top layer denotes the intima-media layer (shear modulus μ_1, wall thickness h_1), and the bottom layer denotes the adventitia (shear modulus μ_2, wall thickness h_2). In the one-layer model, μ and h denotes the shear modulus and wall thickness of the plate, respectively. Parameters in the two-layer model: $\mu_1 = 50$ kPa, $\mu_2 = 150$ kPa, $h_1 = h_2 = 0.5$ mm. Parameters in the one-layer model: $\mu = 100$ kPa, $h = 1$ mm.

Supplementary Note S6. Modal analysis of the axial guided wave generated by the programmed ARFs

The dispersion curve induced by the acoustic radiation force is found to be multimodal (*22*). In order to clarify the dominant modes in the axial guided wave generated by the proposed acoustic radiation force in this study, finite element analysis was conducted. To facilitate a comparison with theoretical models and without loss of generality, we built a tube model in vacuum with isotropic linear elastic material. The tube was firstly stimulated sequentially from the anterior wall to the posterior wall with Gaussian distribution of body force to simulate ARFs (Fig. S14a), and then stimulated at the anterior wall to simulate a single ARF (Fig. S14d). Their corresponding particle velocity maps extracted along the axial direction of the anterior wall are shown in Fig. S14b and e, respectively.

The theoretical axisymmetric L(0,1) and non-axisymmetric (flexural) modes L(N,1) (where $N \geq 1$, N denotes the number of periodic waves in the circumferential direction) of a isotropic tube in vacuum were calculated according to the literature (*82*). With the excitation of multiple ARFs, as shown in Fig. S14c, the dispersion curve is composed of multiple modes at lower frequencies (e.g. < 0.6 kHz), while at high frequencies (e.g. > 1 kHz), L(2,1) mode is the most dominant component and matches best to the dispersion curve. In comparison, with the excitation of a single ARF, as shown in Fig. S14f, the L(0,1) and L(1,1) modes are more dominant since they match best to the FEA data at high frequencies. In summary, different excitation methods result in different dominant modes of guided waves. With the excitation method of multiple ARFs used in this work, the L(2,1) mode predominates at high frequencies (e.g. > 1 kHz). Therefore, it is physically reasonable to use $N = 2$ in Eq. (2) to approximate the dispersion curve of axial guided waves.

It should be noticed that previous study (*78*) showed that an extended ARF beam (e.g. F-number = 3) selects mainly odd modes of axial guided waves (i.e. $N = 1, 3, \ldots$). In our work, the acoustic radiation force is applied sequentially from the anterior to the posterior wall. Although a

narrow Mach cone is formed, the F-number of each individual ARF is around 1.5, which explains why the dominant mode observed in our experiments is $N = 2$.

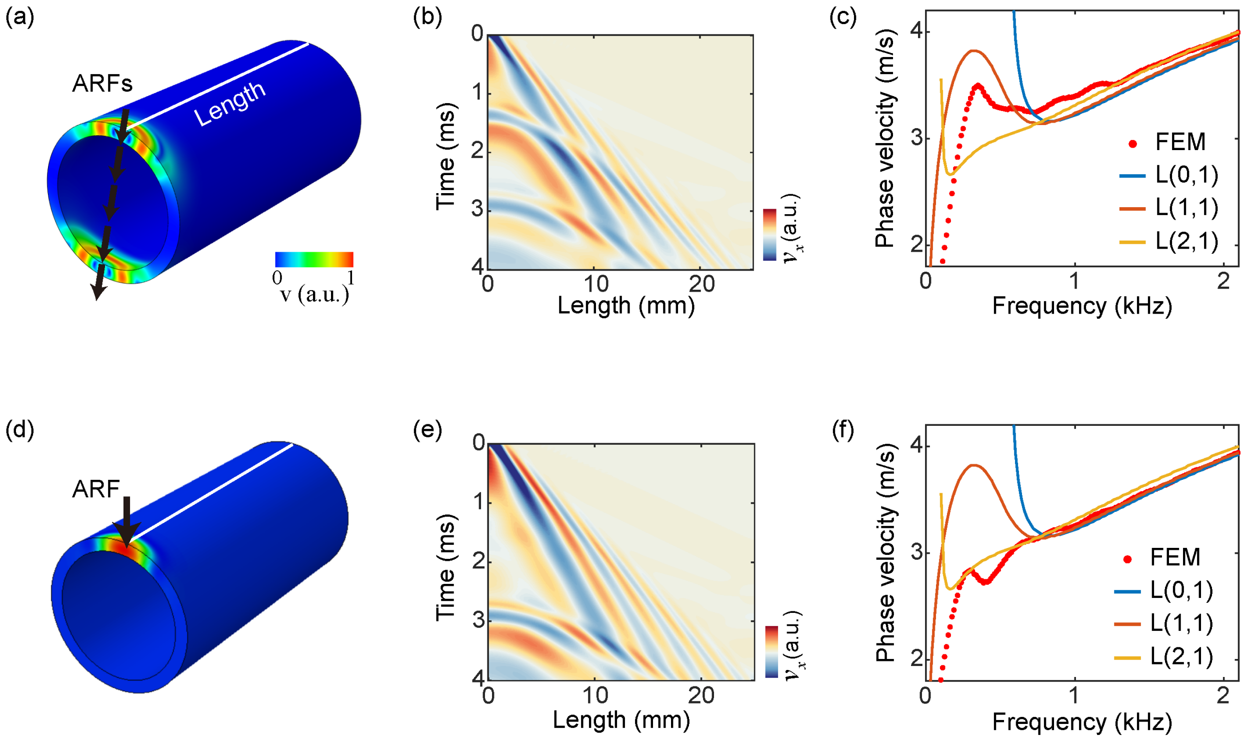

Fig. S14. Modal analysis of axial guided waves with different excitation methods using finite element simulations. (a) Excitation method of multiple ARFs, **(b)** the corresponding particle velocity maps extracted along the anterior wall, and **(c)** comparison of the simulated dispersion and the first three theoretical modes of the axial guided waves, i.e. L(N,1) (N = 0, 1, 2). **(d)** Excitation method of a single ARF, **(e)** the corresponding velocity map, and **(f)** comparison of the simulated dispersion and the theoretical dispersion of axial guided waves. The tube is modeled as linear elastic and isotropic, and is placed in air. Wall thickness 0.5 mm, middle radius 2.75 mm, Young's modulus 100 kPa. FEM: finite element model.

Supplementary Note S7. Verification of the bidirectional guided wave models using finite element analysis

We verify the bidirectional guided wave models proposed in this study using finite element analysis (FEA). As shown in Fig. S15a, the dispersion curve predicted by the axial guided wave model, i.e. Eq. (2) with $N = 2$, which approximates the L(2,1) mode of a tube, agrees well with the FEA result within a frequency range of 0.5 – 1.5 kHz (relative difference < 6%), while the dispersion curve with $N = 0$ (i.e. Lamb wave model) significantly deviates from the FEA results. This confirms that using $N = 2$ in Eq. (2) to approximate the axial guided waves is appropriate. Figure S15b compares the circumferential guided wave dispersion obtained from the FEA and the wave dispersion predicted by the Lamb wave model (Eq. (3)). The two curves match each other well (relative difference < 6% within a frequency range of 0.5 – 1.5 kHz), indicating that it is reasonable to employ the Lamb wave model to analyze the circumferential guided wave dispersion.

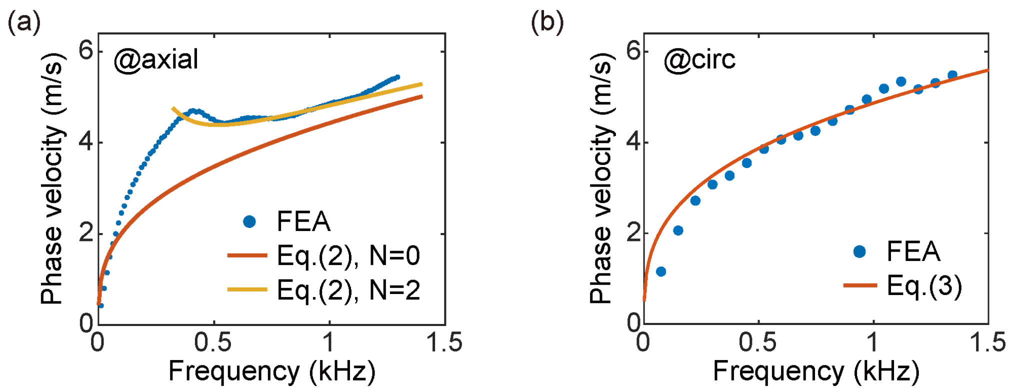

Fig. S15. Verification of the bidirectional guided wave models using finite element analysis (FEA). (a) Dispersion curve of axial guided waves. **(b)** Dispersion curve of circumferential guided waves.

Supplementary Note S8. Mechanical meaning of the acoustoelastic parameter α

Consider the presence of a small shear deformation k in a material subjected to the normal deformation, the deformation gradient tensor is

$$\boldsymbol{F} = \begin{bmatrix} \lambda_1 & k & \\ & \lambda_2 & \\ & & \lambda_3 \end{bmatrix} \tag{S4}$$

The Cauchy stress of the shear component σ_{12} is derived as

$$\sigma_{12} = 2W_1\lambda_2 k \tag{S5}$$

The tangent shear modulus $\mu_{12}^{'}$ can be defined as

$$\mu_{12}^{'} = \frac{\partial \sigma_{12}}{\partial k} \tag{S6}$$

Inserting Eq. (S5) into Eq. (S6), we obtain

$$\mu_{12}^{'} = 2W_1\lambda_2 \tag{S7}$$

Comparing Eq. (S7) and Eq. (S28-e), we find

$$\gamma = \mu_{12}^{'}\lambda_2 \tag{S8}$$

Inserting Eq. (S8) into relation $\sigma_1 = \alpha_1 - \gamma$, we get

$$\alpha_1 = \sigma_1 + \mu_{12}^{'}\lambda_2 \tag{S9}$$

Equation (S9) establishes a quantitative relationship between the acoustoelastic parameter, normal stress, and the tangent shear modulus. The acoustoelastic parameter α reflects the coupled effect of stress and shear modulus, and is therefore referred to as stiffness in this work.

Supplementary Note S9. The effect of windowing on the spatiotemporal velocity map to extract axial dispersion curves

The spatiotemporal velocity field extracted along the longitudinal path contains both axial and circumferential guided wave information. We adopted the FEA results to study the effect of windowing on the map to extract axial guided wave dispersion. Figure S16a shows the raw particle velocity map. Figure S16b shows the frequency-wavenumber field (k-space) by applying 2D-FFT to the raw spatiotemporal map, where the spectral signal of the circumferential guided wave is very distinct. Figure S16c and d demonstrate that applying windowing to the velocity map, followed by a 2D-FFT on the map, results in a k-space map primarily representing the axial guided wave components, with the circumferential wave components effectively suppressed. The dispersion curve is then obtained by searching peaks in the k-space map at each frequency, resulting in a frequency-wavenumber curve (red dotted line in Fig. S16b and d), and the phase velocity is calculated by $c_a^p = f/k$. Figure S16e compares the two dispersion curves, obtained with or without the use of windowing. The one with windowing is smoother, highlighting the necessity of windowing. Figure S16f - h show the extraction of axial guided wave dispersion from the *in vivo* data. As shown, by windowing the spatiotemporal map, the circumferential guided wave signals can be effectively filtered out, and the resulting axial wave dispersion curve is smoother.

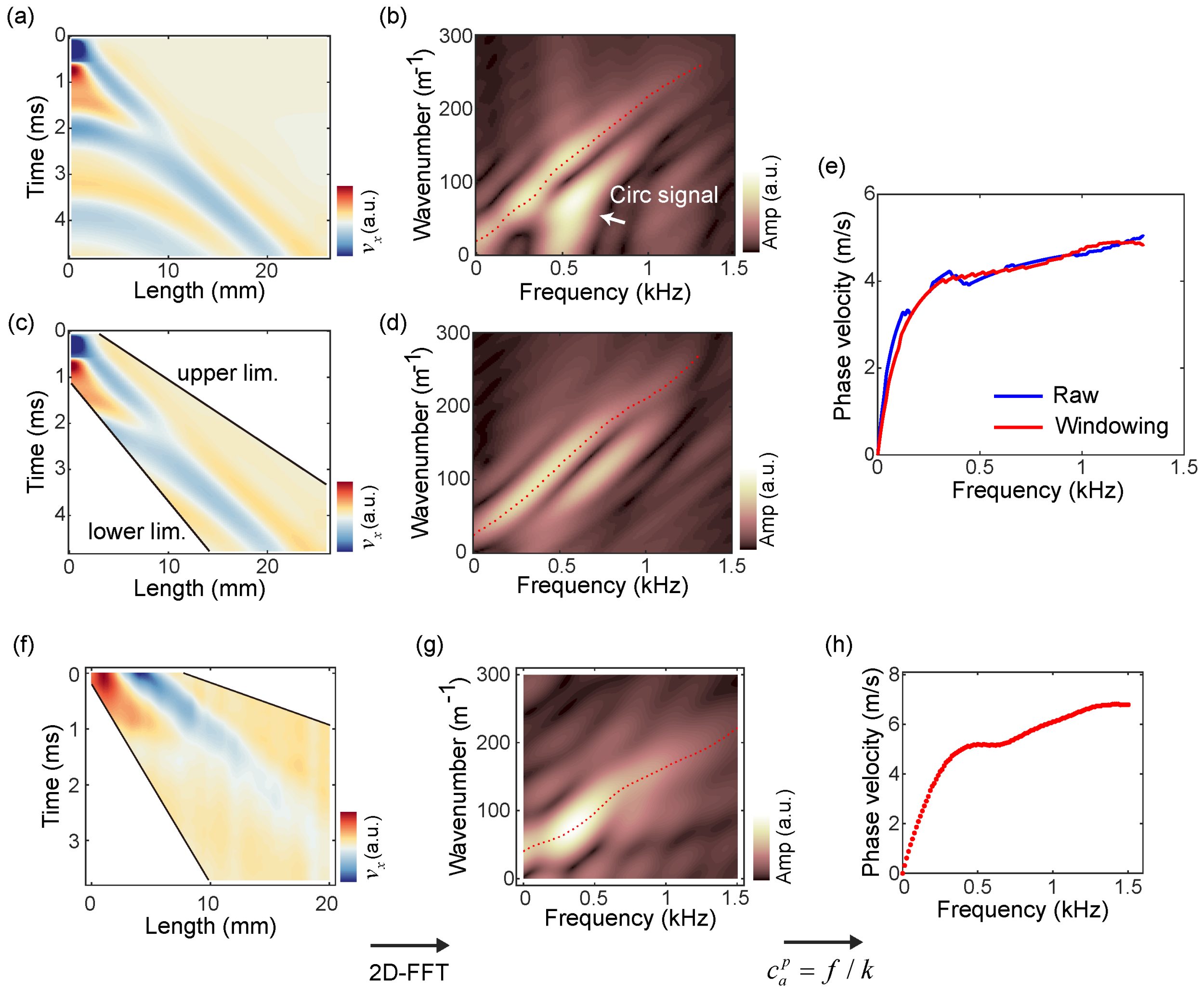

Fig. S16. Extraction of axial guided wave dispersion curves. (a) - (e) Finite element results. (f) - (h) *In vivo* data. **(a)** Raw spatiotemporal velocity map, and **(b)** the corresponding frequency-wavenumber spectrum (*k*-space) by using 2D-FFT. **(c)** Windowing on the spatiotemporal velocity map to filter circumferential signal, and **(d)** the corresponding *k*-space by using 2D-FFT. **(e)** Dispersion curves obtained from the raw velocity map and the windowing map, respectively. **(f)** Windowing on the *in vivo* velocity map, **(g)** the corresponding *k*-space map, and **(h)** the dispersion curve.

Supplementary Note S10. Ex vivo ultrasound elastography experiment of porcine aortas

S10.1 The ultrasound method for axial stress and stiffness characterization

Figure S17a shows the experiment set-up of the guided wave elastography on the porcine aorta. Figure S17b plots the velocity maps extracted from the anterior wall at the axial stretch ratio of 1 (stress-free), 1.23 and 1.31, respectively. Using the axial dispersion data at λ = 1.23 and 1.31, we were able to identify the parameters $\alpha_{a,1}$, $\alpha_{a,2}$, γ_1, γ_2, g and τ, where the subscripts '1' and '2' refer to the state of λ = 1.23 and 1.31, respectively. These values are listed in Table. S2. Figure S17c shows the fitting results of the dispersion data. The axial stresses at the two states were then calculated (using $\sigma_a = \alpha_a - \gamma$) and compared with the results from the tensile tests. As suggested by Fig. S17d, the axial stress can be reliably measured using the ultrasound method, with a relative error of less than 9% compared to the tensile test.

Figure S18a presents stress - stretch curves obtained from uniaxial tensile test along the axial and circumferential directions of the aorta samples. By fitting them to the Gasser-Ogden-Holzapfel model, the constitutive parameters of the aorta can be obtained: $\mu = 38.3$ kPa, $k_1 = 111.2$ kPa, $k_2 = 11.5$, $\kappa = 0.202$, $\varphi = 39.5°$. The fiber dispersion parameter κ, which reflects the microstructural organization of the arterial wall, is commonly measured by statistical analysis on microscopy images (*74*). Reported values of κ typically fall within a narrow range of 0.19 – 0.23 (67, *83*). Our analysis indicates that uncertainties in κ within this range have negligible effects on the inferred acoustoelastic parameters α and γ. Inserting the above constitutive parameters into Eq. (S30), we were able to calculate the acoustoelastic parameters α_a and γ, and compare them to those obtained by the ultrasound method. As listed in Table S2, the relative errors of the parameters α_a, γ measured from the ultrasound method and the tensile test are less than 10%.

The two viscous parameters g and τ were further obtained by fitting the dispersion data at stress-free state to the theoretical model: g = 0.66 and $\tau = 0.077$ ms (Fig. S18b). As listed in Table S2, the relative error of g is ~9%, while the relative error of τ is high, indicating that the

current ultrasound method is not reliable enough to characterize the viscoelastic parameter τ.

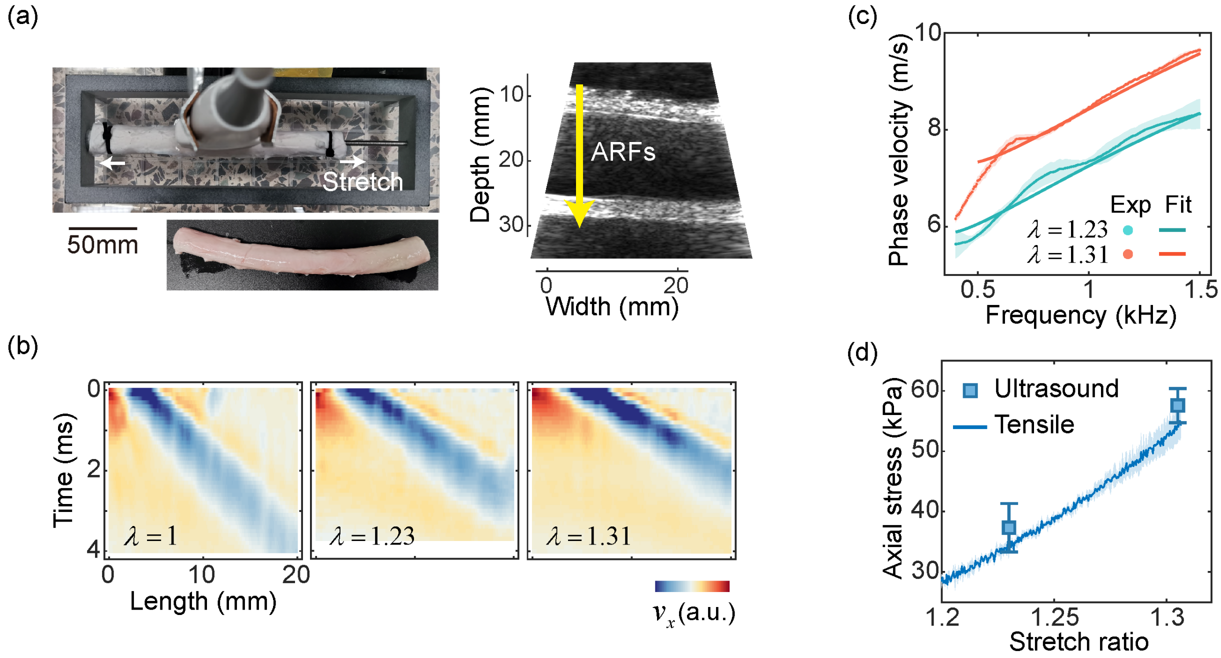

Fig. S17. Ultrasound elastography experiment on the porcine aorta. (a) Experimental setup. Left: Photography. The sample is clamped at both ends, and a displacement-controlled stretching is applied. Right: Ultrasound B-mode image of the artery sample. **(b)** Spatiotemporal particle velocity maps with axial stretch of λ = 1, 1.23 and 1.31. **(c)** Dispersion curves and fitting results with stretch ratio of 1.23 and 1.31. **(d)** Comparison of the axial stresses obtained by the ultrasound method and the tensile test.

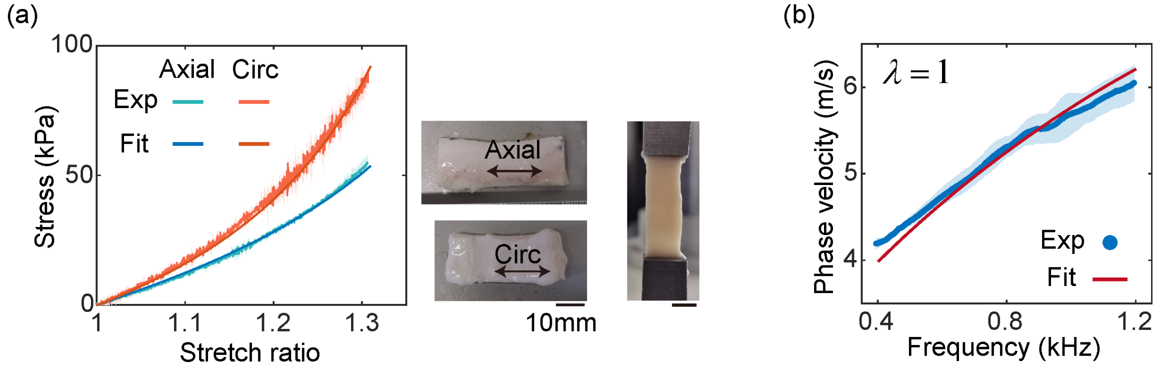

Fig. S18. Mechanical characterization of the porcine aorta. (a) Tensile tests along the axial and

circumferential directions of the aorta sample, respectively. **(b)** Axial guided wave dispersion at the stress-free state.

S10.2 A discussion on the circumferential guided waves in the porcine aorta samples

From the velocity map of the *ex vivo* aorta, the circumferential time marker—time phase t_1 is less obvious to be detected (Fig. S17b). This may be attributed to the fact that the radius of the porcine aorta is much larger than the that of the human carotid artery (approximately 7 mm v.s. 3 mm). As a result, the travel distance of the circumferential guided wave from the posterior wall to the anterior wall in the porcine aorta is longer, and the attenuation of waves could be more pronounced, leading to a less distinct arrival signal. To verify this assumption, we increased the ultrasound imaging duration (10 ms) to measure wave propagation in the aorta sample (Fig. S19a). The time phase t_1 can be detected at ~ 4 ms in the velocity map; however, the velocity peak remains relatively weak.

We also conducted finite element analysis (FEA). The axial and circumferential Young's moduli of the aorta sample are $E_a = 116.1$ kPa and $E_c = 197.7$ kPa, respectively, as measured from the tensile test (Fig. S19c). Applying these material parameters and aortic geometry into FEA model, the velocity map was obtained and presented in Fig. S19b. The simulation results are similar to the experimental observations, that is the amplitude of the minimum peak at t_1 is relatively weak.

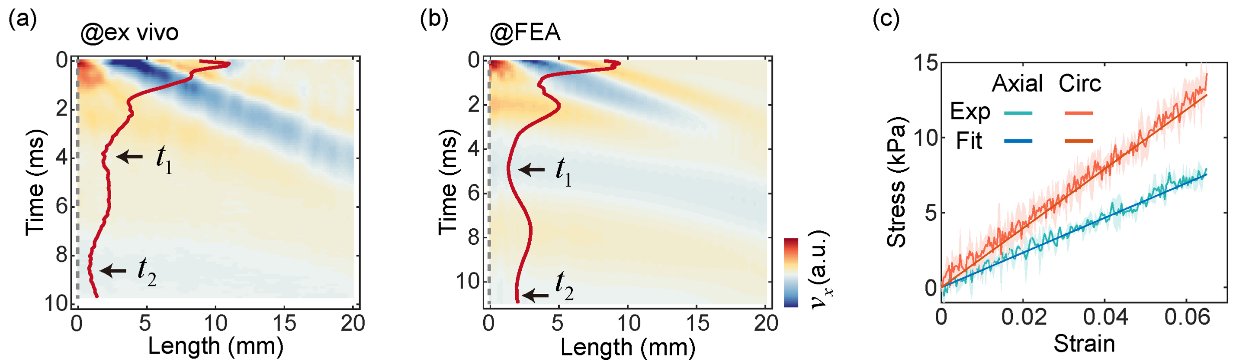

Fig. S19. Measurement of bidirectional guided waves in the *ex vivo* sample. (a) Particle velocity map obtained from the *ex vivo* experiment at the stress-free state. **(b)** Particle velocity map obtained from the FEA. Material parameters used in FEA: $E_a = 116.1$ kPa, $E_c = 197.7$ kPa, $\mu_a = 38.3$ kPa, $\mu_c = 87$ kPa. Wall thickness 3 mm, middle radius 7.5 mm. **(c)** Uniaxial tensile tests of the aorta sample along axial and circumferential directions, respectively.

Supplementary Note S11. A discussion on the difference between bidirectional stiffnesses and bidirectional group wave velocities

The axial group velocity c_a can be implicitly expressed by:

$$c_a = \mathcal{F}_1(f_c, \alpha_a, g, \tau, h, r, \rho, \rho_f, \kappa_f) \quad \text{(S10)}$$

where f_c is central frequency of group velocities (~0.5 kHz). α_a is the axial stiffness. g and τ are two viscous parameters. h denotes wall thickness. r denotes the radius of the artery. ρ and ρ_f denotes the density of arterial wall and blood, respectively. κ_f is the bulk modulus of blood. The circumferential group velocity c_c can be implicitly expressed by:

$$c_c = \mathcal{F}_2(f_c, \alpha_c, g, \tau, h, \rho, \rho_f, \kappa_f) \quad \text{(S11)}$$

where α_c is the circumferential stiffness. For each individual, the ratio of c_a and c_c can be simplified as

$$\frac{c_a}{c_c} \approx \frac{\mathcal{F}_1(\alpha_a)}{\mathcal{F}_2(\alpha_c)} \quad \text{(S12)}$$

Due to the difference in functions $\mathcal{F}_1$ and $\mathcal{F}_2$ (i.e., the difference of wave dispersion for axial and circumferential guided waves), the ratios c_a/c_c and α_a/α_c can be different. Figure S20a shows the *in vivo* results of all the participants. The ratio of bidirectional group velocities is approximately 1, whereas the ratio of bidirectional stiffness α_c/α_a varies from approximately 1 to 2. We further conducted systematic finite element simulations (110 cases in total), and the results show that the ratio α_c/α_a is generally greater than the ratio c_c/c_a (Fig. S20b), consistent with our experimental observations.

In vivo experiments revealed slight differences in the trends of stiffness and group wave velocity across the three groups. This can be explained by Eqs. (S10) and (S11). Taking axial guided waves as an example, group wave velocity c_a is not only related to stiffness α_a, but also influenced by viscosity (g, τ), geometry (h, r), and frequency (f). Figure S21a present the relationship between the axial group velocity and axial stiffness from the *in vivo* results. The Pearson correlation coefficient (r = 0.50) indicates a modest correlation between the two variables.

The circumferential group velocity and circumferential stiffness also exhibit a modest correlation (r = 0.56, Fig. S21b). These results confirm that group velocity and stiffness do not follow a simple linear relationship.

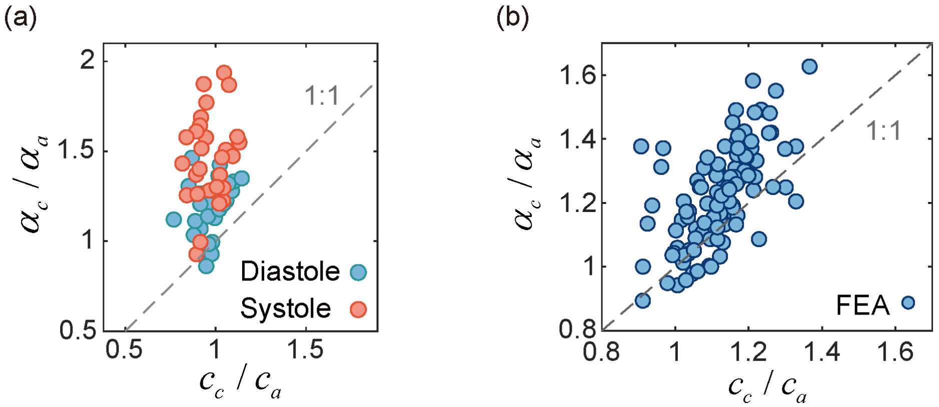

Fig. S20. Relationship between the ratio of bidirectional group velocities (c_c/c_a) and the ratio of bidirectional stiffnesses (α_c/α_a). (a) In vivo experimental data of all participants. **(b)** Results of finite element analysis (FEA). A total of 110 numerical cases are presented. The model parameters fall within the following range, based on the in vivo results: $40 \leq \alpha_a \leq 110$ kPa, $45 \leq \alpha_c \leq 145$ kPa, $0.4 \leq g \leq 0.9$, $0.01 \leq \tau \leq 0.1$ ms, $3 \leq r \leq 4$ mm, $0.8 \leq h \leq 1.2$ mm,$1 < \lambda_z \leq 1.1$, $\rho = \rho_f = 1000$ kg/m^3, $\kappa_f = 2.2$ GPa.

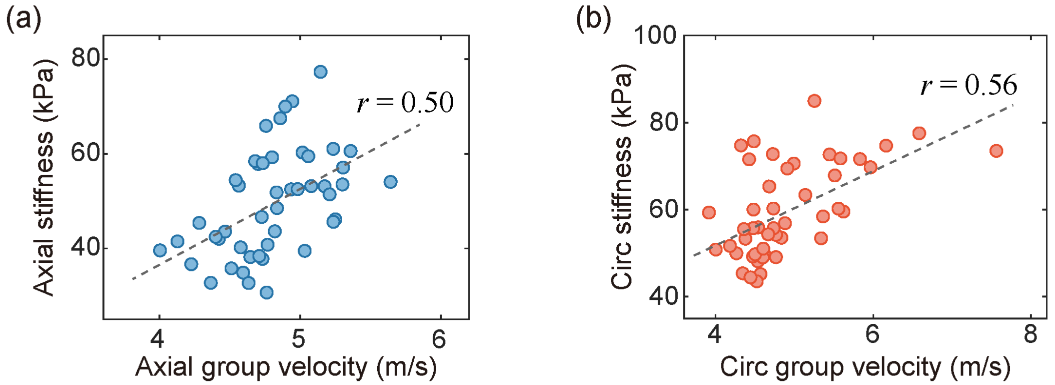

Fig. S21. Relationship between the stiffness and group wave velocity from in vivo data of all the participants. (a) Axial stiffness (α_a) versus axial group velocity (c_a) at diastole. **(b)** Circumferential stiffness (α_c) versus circumferential group velocity (c_c) at diastole.

Supplementary Note S12. Selection for the number of consecutive ARFs and its impact on the arterial guided waves

In each single ultrasound focusing, the spatial distribution of acoustic radiation force near the focal point approximately follows a Gaussian distribution, with a Gaussian radius of about 2.1 mm along the depth direction (see details in Supplementary Note 13). Imagining the excitation process of multiple ARFs focused consecutively from the anterior wall to the posterior wall— even if most of them are focused inside the arterial lumen rather than on the arterial walls, a portion of the acoustic energy can still be transmitted to the arterial walls due to the spatial bandwidth of each single ARF. To confirm our hypothesis, we conducted finite element analysis (FEA). Figure S22a – c shows the simulation results for exciting 2, 4, and 10 ARFs on the arterial walls, respectively. As the number of ARF increases, the amplitude of the particle velocity increases significantly. As suggested by Fig. S22d, the velocity peak with 10 ARFs excitation is ~4 times higher than that with 2 ARFs excitation. These simulation results verify that even when some ARFs are not focused on the arterial walls, they still deliver part of the excitation energy, thereby enhancing the signal of arterial guided waves.

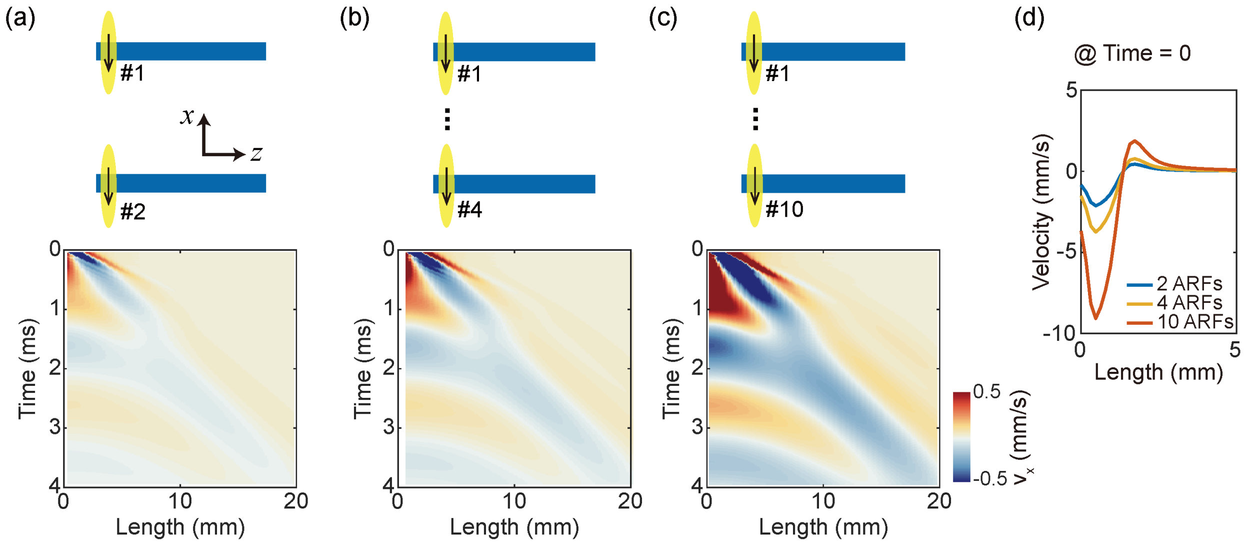

Fig. S22. Finite element simulation results with varying numbers of acoustic radiation forces. **(a)** Top: Schematic of excitation with 2 ARFs sequentially from anterior to posterior walls. Bottom: The corresponding spatiotemporal particle velocity map extracted from the anterior wall along the axial direction. **(b)** Top: Schematic of excitation with 4 ARFs. Bottom: The corresponding velocity map. **(c)** Top: Schematic of excitation with 10 ARFs. Bottom: The corresponding velocity map. **(d)** Comparison of velocity curves obtained from the above three excitation cases at the initial time point. Simulation parameters: $\mu = 20$ kPa, $k_1 = 120$ kPa, $k_2 = 2$, $\kappa = 0.226$, $\varphi = 50°$, $g = 0.6$, $\tau = 0.03$ ms, $r = 3$ mm, $h = 1$ mm, $\lambda_z = 1.05$. Inner pressure = 75 mmHg.

Supplementary Note S13. Selection of the compensation time and its impact on the measurement error of circumferential group wave velocities

According to Eq. (4), an appropriate selection of the compensation time t_c is important for the measurement of circumferential group wave velocities. In our *in vivo* experiments, the ARFs were sequentially induced from the anterior wall to the posterior wall, resulting in a time delay between the guided waves excited on the anterior and posterior walls. Therefore, it is necessary to examine the impact of this time delay, and thereby optimizing the selection of time t_c. In the following, we firstly calculate the spatial distribution of a single ARF. Then we conduct finite element analysis (FEA) with the determined spatial distribution and temporal sequence of ARFs, aiming to quantify the time delay between two walls and study the measurement error of circumferential wave velocities.

Figure S23a presents the spatial distribution of acoustic pressure obtained from FEA. A 2D finite element model with ~2,000,000 acoustic elements was built. Similar to the settings in the ultrasound experiment, 32 elements (spanning ~10 mm) were sinusoidally driven with specific time delays for each element to ensure that all acoustic waves would converge at the depth of ~15 mm. The spatial distribution of acoustic pressure near the focal region can be approximated by a Gaussian function with Gaussian radius r_x and r_z in the x (the axial direction of ultrasound beam) and z axis (the lateral direction of ultrasound beam), respectively. Figure S23b plots the spatial distribution of acoustic pressure along the x and z axis, with Gaussian radius r_x = 0.2 mm, and r_z = 3 mm. Our previously experimentally calibrated acoustic pressure distribution along the z axis aligns well with this simulation result (*20*). Considering that the acoustic radiation force (ARF) is proportional to the square of acoustic pressure, the Gaussian radii for the ARF are approximately r_x = 0.14 mm, and r_z = 2.1 mm.

By configuring the spatial distribution of ARF with specified Gaussian radii, and sequentially exciting 10 ARFs within 0.5 ms, we simulated the propagation of guided waves in arteries by FEA

(Fig. S23c). Figure S23d displays the spatiotemporal velocity maps extracted from the anterior wall, the posterior wall, and the circumferential path within half a circle. The time phases t_1 detected at the anterior wall ($t_{1,a}$) and at the posterior wall ($t_{1,p}$) show a time lag of ~0.2 ms. It is reasonable because the anterior wall is excited in advance compared to the posterior wall by ARFs, so that the circumferential guided wave initiated from the anterior wall arrives at the posterior wall earlier than the opposite-side wave arrives at the anterior wall. In principle, the velocity c_c can be calculated using either of the following two equations,

$$c_c = \pi r_c / \left(t_{1,a} + t_{c,a}\right) \tag{S13}$$

$$c_c = \pi r_c / \left(t_{1,p} + t_{c,p}\right) \tag{S14}$$

where $t_{c,a}$ and $t_{c,p}$ denote the compensation time for use with the anterior wall and posterior wall, respectively. In the *in vivo* experiments, we adopted $t_c = 0.8$ ms for both the anterior wall and posterior walls. As illustrated in Fig. S23e, when both $t_{c,a}$ and $t_{c,p}$ are set to 0.8 ms, compared to the truth value of c_c (it is obtained by measuring the slope in the map along the circumferential path, see dashed line in Fig. S23d), the calculation value of c_c from the anterior wall by using Eq. (S13) will slightly underestimate it, whereas the calculation of c_c from the posterior wall by using Eq. (S14) will slightly overestimate it. However, the relative error for both of them is less than 6%, indicating that the measurement error in circumferential group wave velocities introduced by approximating $t_c = 0.8$ ms is negligible.

In the ultrasound experiments, the temporal resolution is 0.1 ms, therefore it is difficult to accurately measure the time difference between $t_{1,a}$ and $t_{1,p}$. Figure S23f displays *in vivo* results from a young volunteer, where $t_{1,a}$ and $t_{1,p}$ were measured from the corresponding maps. Statistical analysis revealed no significant difference between the two time phases. This is because that the temporal resolution of the experiment is insufficient to distinguish the time difference between $t_{1,a}$ and $t_{1,p}$ (< 0.2 ms). This also validates the experimental rationale of uniformly assigning $t_c = 0.8$ ms to both the anterior and posterior walls.

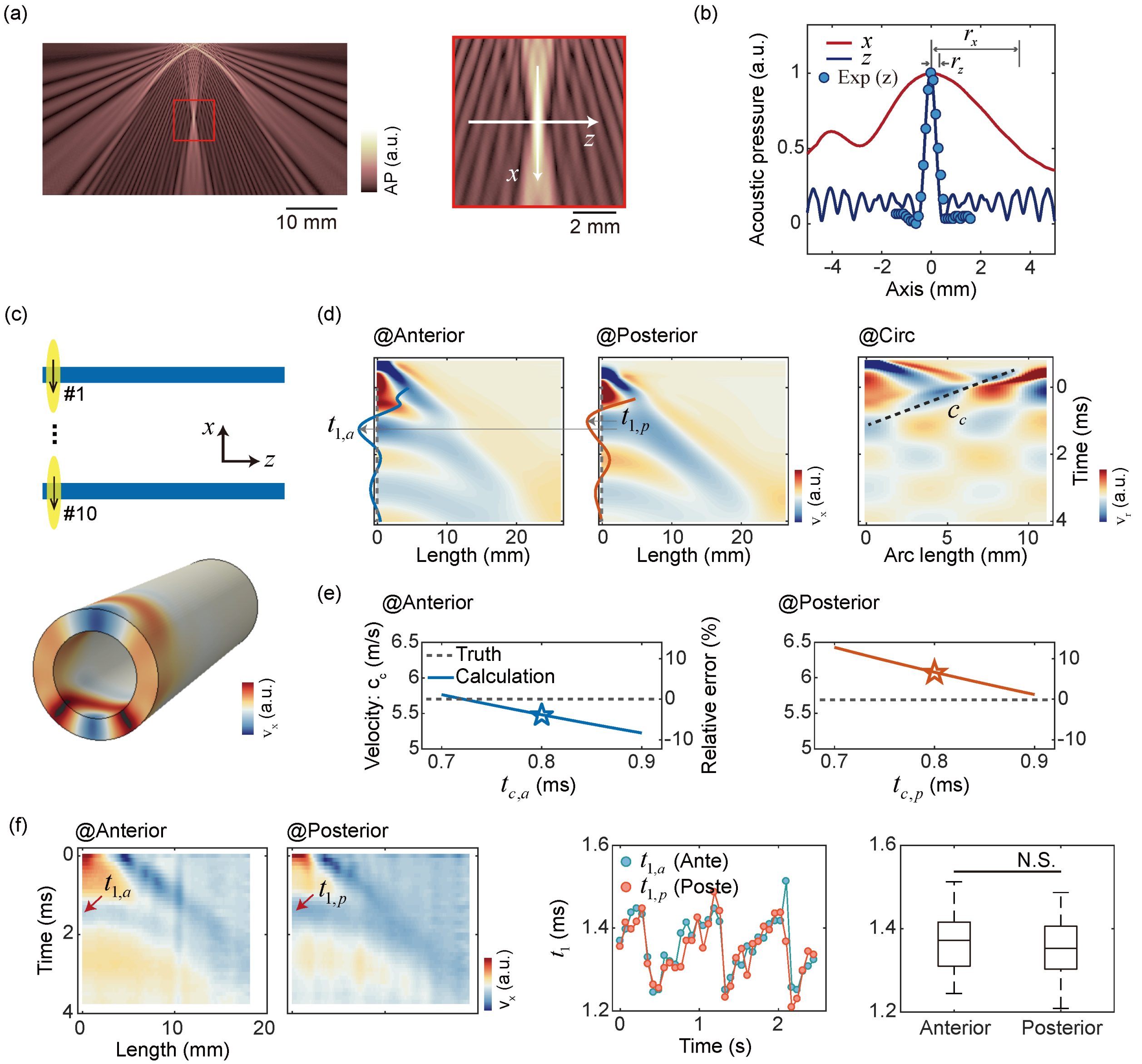

Fig. S23. A discussion on the selection of compensation time t_c. (a) FEA to simulate the spatial distribution of acoustic pressure with a single ARF. AP: Acoustic pressure. **(b)** Distribution of acoustic pressure along the *x*- and *z*-axis. Curves: FEA results. Dots: experimental data from our previous work (*20*). **(c)** FEA model with 10 consecutive ARFs. **(d)** Particle velocity maps extracted from the anterior wall, the posterior wall and the circumferential path. **(e)** Relative error of the measurement of c_c from the anterior wall and posterior wall. **(f)** *In vivo* experimental results from a young volunteer, including particle velocity maps at two walls, variation of t_1 in cardiac cycles, and statistical comparison of $t_{1,a}$ and $t_{1,p}$.

Supplementary Note S14. Comparison of the two viscoelastic models

Biomaterials exhibit a power-law viscoelastic response. Compared with the Prony series model adopted in this study, it is more appropriate to describe the viscoelasticity of arterial walls with the Kelvin-Voigt fractional derivative (KVFD) model (*71*). The constitutive parameters of the KVFD model include fractional order δ $(0 < \delta < 1)$, and viscous parameter η (dimension $[s^{\delta}]$). In order to illustrate the feasibility of using the Prony series model in this study, we compare the dispersion curves predicted by the two models (*79*). As shown in Fig. S24, when the frequency is below 1.5 kHz, the two dispersion curves are basically consistent. This suggests that the first-order Prony series model can effectively capture the viscoelastic behavior of the arteries within the frequency range measured by ultrasound elastography. As the frequency increases, for example, exceeding 10 kHz, the dispersion of the KVFD model continues to rise steadily, while the dispersion of the Prony series model reaches plateaus. This suggests that at higher frequencies (e.g. above 2 kHz, as detected by optical coherence elastography), the current Prony-series model may not be adequate.

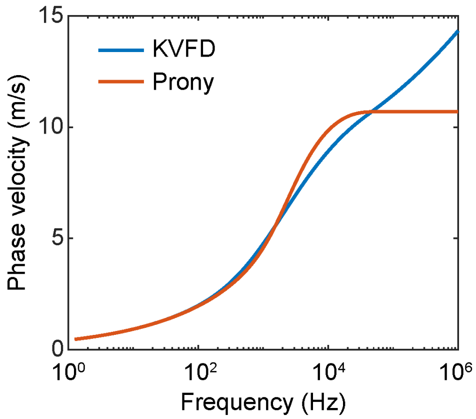

Fig. S24. Comparison of the two viscoelastic models. Plane shear wave velocities are calculated here for simplicity. The parameters of the KVFD model are $\eta = 0.1\ \text{s}^{\delta}$, $\delta = 0.25$. The parameters of the Prony series model are $g = 0.7$, $\tau = 6 \times 10^{-5}$ s.

Supplementary Note S15. Guided wave model for pre-stressed viscoelastic flat plates

Consider a viscoelastic pre-stressed flat plate immersed in inviscid fluid (Fig. S12b). A Cartesian coordinate system is defined on the plate, with the three directions referred to as axial (x_z), circumferential (x_θ), and radial directions (x_r). The wall thickness is h. In the incremental dynamics theory of viscoelastic materials, the incremental stress $\boldsymbol{\Sigma}$ is related to the displacement of wave motion $\boldsymbol{u}$ as follows (*79*)

$$\Sigma_{ji} = -\hat{q}\delta_{ji} + qu_{j,i} - G\hat{Q}\delta_{ji} + GQu_{j,i} + G\mathcal{A}_{0jikl}u_{l,k} - \Omega\sigma^{e}_{Djk}u_{i,k} \quad \text{(S15)}$$

where j and i correspond to the directional indices r, θ and z (Note that r, θ, and z do not represent cylindrical coordinates here, but correspond to the three Cartesian directions x_r, x_θ, and x_z). $\hat{q}$ denotes the increment of the Lagrange multiplier q. $\hat{Q}$ is the increment of the volumetric part of the elastic stress Q $(= \sigma^{e}_{ii}/3)$. $\mathcal{A}_{0jikl} = \left(\partial^2 W / \partial F_{ip}\partial F_{lq}\right)F_{jp}F_{kq}$ (where $j, i, k, l, p, q \in (r, \theta, z)$) is the fourth-order Eulerian elasticity tensor (*84*). G and Ω are two frequency-dependent parameters

$$G = \left(1 - \frac{g}{1+i\omega\tau}\right)/\left(1-g\right), \quad \Omega = \left(\frac{g\omega^2\tau^2}{1+\omega^2\tau^2} + i\frac{g\omega\tau}{1+\omega^2\tau^2}\right)/\left(1-g\right) \quad \text{(S16)}$$

where i denotes the imaginary unit. Viscous parameters g and τ denote the relaxation amplitude and characteristic relaxation time of arteries, respectively. ω $(= 2\pi f)$ denotes the angular frequency.

When the guided wave propagates along the axial direction, assuming that the wave has displacement components only within the x_z-x_r plane (i.e. $u_\theta = 0$), a stream function $\psi(x_z, x_r, t)$ can be introduced to replace displacements with $u_z = \psi_{,r}$ and $u_r = -\psi_{,z}$. Inserting Eq. (S15) and ψ into Eq. (1), the wave motion equation can be obtained as

$$G\left(\alpha_a\psi_{,zzzz} + 2\beta_a\psi_{,zzrr} + \gamma\psi_{,rrrr}\right) - \Omega\left[\sigma^{e}_{Dzz}\psi_{,zzzz} + \sigma^{e}_{Drr}\psi_{,rrrr} + (\sigma^{e}_{Dzz} + \sigma^{e}_{Drr})\psi_{,zzrr}\right] = \rho(\psi_{,zztt} + \psi_{,rrtt}) \quad \text{(S17)}$$

where

$$\sigma_{Dzz}^{e} = \frac{2}{3}\alpha_a - \frac{1}{3}\gamma - \frac{1}{3}\alpha_c,\ \sigma_{Drr}^{e} = \frac{2}{3}\gamma - \frac{1}{3}\alpha_a - \frac{1}{3}\alpha_c \tag{S18}$$

The long-term incremental parameters α_a, α_c, γ, β_a are defined as $\alpha_a = \mathcal{A}_{0zrzr}$, $\alpha_c = \mathcal{A}_{0\theta r\theta r}$, $\gamma = \mathcal{A}_{0rzrz} = \mathcal{A}_{0r\theta r\theta}$, $\beta_a = (\mathcal{A}_{0zzzz} + \mathcal{A}_{0rrrr} - 2\mathcal{A}_{0zzrr} - 2\mathcal{A}_{0zrrz})/2$. Since the elastic wave propagates along the x_z axis, the stream function has a harmonic form of $\psi = \psi_0 \exp(skx_r)\exp[i(kx_z - \omega t)]$, where ψ_0 is an amplitude. s denotes a ratio of wavenumbers in the two directions. k denotes the complex wavenumber. Inserting ψ into Eq. (S17) yields

$$(\gamma + \Omega Q)s^4 + \left[\rho\frac{\omega^2}{k^2} - 2G\beta_a + \Omega(\alpha_a + \gamma - 2Q)\right]s^2 + \alpha_a + \Omega Q - \rho\frac{\omega^2}{k^2} = 0 \tag{S19}$$

where

$$Q = (\alpha_a + \gamma + \alpha_c)/3 \tag{S20}$$

At the interface between the plate and fluid, the conditions of normal displacement continuity, normal stress continuity, and zero shear stress are satisfied, which can be expressed as:

$$u_r = u_r^f, \Sigma_{rz} = 0, \Sigma_{rr} = -p^f, \text{at } x_r = \pm h/2 \tag{S21}$$

where u^f denotes the displacement of fluid. p^f denotes the hydrostatic pressure of fluid. Using the boundary conditions Eq. (S21), we can derive the dispersion equation for the antisymmetric mode of guided waves as follows (*79*):

$$\begin{aligned}&\left(1+{s_{2a}}^2\right)\cdot\left(-\rho\frac{\omega^2}{k^2}s_{1a} + C_{1a}s_{1a} - C_{2a}{s_{1a}}^3\right)\cdot\tanh\left(s_{1a}kh/2\right)\\ &-\left(1+{s_{1a}}^2\right)\cdot\left(-\rho\frac{\omega^2}{k^2}s_{2a} + C_{1a}s_{2a} - C_{2a}{s_{2a}}^3\right)\cdot\tanh\left(s_{2a}kh/2\right) + \left({s_{1a}}^2 - {s_{2a}}^2\right)\frac{\rho^f}{\xi}\frac{\omega^2}{k^2} = 0\end{aligned} \tag{S22}$$

where s_{1a} and s_{2a} are two roots solved by the quartic equation (S19), and

$$C_{1a} = 2G\beta_a + \gamma + \Omega\alpha_c \tag{S23}$$

$$C_{2a} = \gamma + \Omega Q \tag{S24}$$

$$\xi^2 = 1 - \frac{\omega^2}{k^2}\frac{1}{c_f^{\ 2}} \tag{S25}$$

where ρ and ρ^f denote the density of the arterial wall (1000 kg/m^3) and blood (1000 kg/m^3), respectively. The speed of sound in the fluid is $c_f = \sqrt{\kappa_p / \rho^f}$. κ_p denotes the bulk modulus of the fluid (= 2.2 GPa for blood). Parameter sensitivity analysis indicates that the parameters α_c and β_a have a minimal effect on axial guided waves (see details in Supplementary Note 19). Therefore, we insert $\alpha_c = 1.5\alpha_a$ and $\beta_a = 4\alpha_a$ into Eq. (S22), and as a result we obtain Eq. (10) in the main text. The rationale for using the relations $\alpha_c = 1.5\alpha_a$ and $\beta_a = 4\alpha_a$ is supported by experimental observations and numerical calculations (see Supplementary Note 17).

As for the waves propagating along circumferential direction, the dispersion equation for the antisymmetric mode of guided waves can be derived in a similar way, which yields

$$\begin{aligned}&\left(1+s_{2c}^{\ 2}\right)\cdot\left(-\rho\frac{\omega^2}{k^2}s_{1c}+C_{1c}s_{1c}-C_{2c}s_{1c}^{\ 3}\right)\cdot\tanh\left(s_{1c}kh/2\right)\\&-\left(1+s_{1c}^{\ 2}\right)\cdot\left(-\rho\frac{\omega^2}{k^2}s_{2c}+C_{1c}s_{2c}-C_{2c}s_{2c}^{\ 3}\right)\cdot\tanh\left(s_{2c}kh/2\right)+\left(s_{1c}^{\ 2}-s_{2c}^{\ 2}\right)\frac{\rho^f}{\xi}\frac{\omega^2}{k^2}=0\end{aligned} \tag{S26}$$

where s_{1c} and s_{2c} are two roots solved by the quartic quation

$$\left(\gamma+\Omega Q\right)s^4+\left[\rho\frac{\omega^2}{k^2}-2G\beta_c+\Omega\left(\alpha_c+\gamma-2Q\right)\right]s^2+\alpha_c+\Omega Q-\rho\frac{\omega^2}{k^2}=0 \tag{S27}$$

and

$$C_{1c} = 2G\beta_c + \gamma + \Omega\alpha_a \tag{S28}$$

$$C_{2c} = \gamma + \Omega Q \tag{S29}$$

where long-term incremental parameter β_c is defined as $\beta_c = (\mathcal{A}_{0\theta\theta\theta\theta} + \mathcal{A}_{0rrrr} - 2\mathcal{A}_{0\theta\theta rr} - 2\mathcal{A}_{0\theta rr\theta})/2$. Given that the parameters α_a and β_c have a minimal effect on circumferential guided waves, by inserting $\alpha_a = 0.7\alpha_c$ and $\beta_c = 4\alpha_c$ into Eq. (S26), we can obtain Eq. (16) in the main text. The rationale for using the relations $\alpha_a = 0.7\alpha_c$ and $\beta_c = 4\alpha_c$ is supported by

experimental observations and numerical calculations (see Supplementary Note 17).

Supplementary Note S16. Explicit forms of acoustoelastic parameters

The general definition of the fourth-order Eulerian elasticity tensor $\mathcal{A}_{0jikl}$ can be found in previous literature (*84*). In particular, for the strain energy function of Eq. (9), the acoustoelastic parameters have the following explicit forms:

$$\alpha_a = 2W_1\lambda_z{}^2 + 2W_4\lambda_z{}^2\sin^2\varphi + 2W_6\lambda_z{}^2\sin^2\varphi \tag{S30-a}$$

$$\begin{aligned}\beta_a =& W_1\left(\lambda_z{}^2+\lambda_r{}^2\right) + W_4\lambda_z{}^2\sin^2\varphi + W_6\lambda_z{}^2\sin^2\varphi + 2W_{11}\left(\lambda_z{}^2-\lambda_r{}^2\right)^2 \\ &+ 4W_{14}\lambda_z{}^2\sin^2\varphi\left(\lambda_z{}^2-\lambda_r{}^2\right) + 4W_{16}\lambda_z{}^2\sin^2\varphi\left(\lambda_z{}^2-\lambda_r{}^2\right) \\ &+ 2W_{44}\lambda_z{}^4\sin^4\varphi + 2W_{66}\lambda_z{}^4\sin^4\varphi\end{aligned} \tag{S30-b}$$

$$\alpha_c = 2W_1\lambda_\theta{}^2 + 2W_4\lambda_\theta{}^2\cos^2\varphi + 2W_6\lambda_\theta{}^2\cos^2\varphi + k_{\mathrm{act}}\lambda_\theta\left[1-\left(\frac{\lambda_m-\lambda_\theta}{\lambda_m-\lambda_0}\right)^2\right] \tag{S30-c}$$

$$\begin{aligned}\beta_c =& W_1\left(\lambda_\theta{}^2+\lambda_r{}^2\right) + W_4\lambda_\theta{}^2\cos^2\varphi + W_6\lambda_\theta{}^2\cos^2\varphi + 2W_{11}\left(\lambda_\theta{}^2-\lambda_r{}^2\right)^2 \\ &+ 4W_{14}\lambda_\theta{}^2\cos^2\varphi\left(\lambda_\theta{}^2-\lambda_r{}^2\right) + 4W_{16}\lambda_\theta{}^2\cos^2\varphi\left(\lambda_\theta{}^2-\lambda_r{}^2\right) \\ &+ 2W_{44}\lambda_\theta{}^4\cos^4\varphi + 2W_{66}\lambda_\theta{}^4\cos^4\varphi + k_{\mathrm{act}}\lambda_\theta{}^2\frac{\lambda_m-\lambda_\theta}{\left(\lambda_m-\lambda_0\right)^2}\end{aligned} \tag{S30-d}$$

$$\gamma_a = \gamma_c = \gamma = 2W_1\lambda_r{}^2 \tag{S30-e}$$

where λ_θ, λ_z and λ_r denote circumferential, axial, and radial stretch ratio, respectively. $W_i = \partial W / \partial I_i$, $W_{ij} = \partial^2 W / \partial I_i \partial I_j$.

Supplementary Note S17. Parameter space of the acoustoelastic and viscoelastic parameters

The dispersion relation, Eq. (S22), contains six unknown parameters, i.e., nonlinear elastic (acoustoelastic) parameters α_a, α_c, β_a, γ, and viscous parameters g, τ. In order to determine the parameter space of the four acoustoelastic parameters, we firstly give the parameter space of the constitutive parameters based on literature reports, they are $5 < \mu < 50$ kPa , $10 < k_1 < 500$ kPa , $0 < k_2 < 100$, $0 < \varphi < \pi / 2$, $0 < \kappa < 1/3$, $0 < k_{\text{act}} < 100$ kPa , $0.8 < \lambda_0 < 1.0$, $1.6 < \lambda_m < 1.8$ (*35, 49-52, 67*). The ranges of bidirectional stretch ratios are: circumferential stretch $1 \le \lambda_\theta \le 1.6$, axial stretch $1 \le \lambda_z \le 1.2$ (*51-52*). By using Eq. (S30), we can determine the parameter space of the acoustoelastic parameters: $20 \le \alpha_a \le 200$ kPa , $0 < \gamma / \alpha_a < 1$, $\beta_a / \alpha_a > 2$ and $\alpha_c / \alpha_a > 0.8$.

We further investigated the parameter space of the viscoelastic parameters g and τ by using FEA. As shown in Fig. S25b, when τ ranges from 10^{-5} to 10^{-4} s and g ranges from 0.3 to 0.9, the particle velocity map closely aligns with the *in vivo* experimental results. However, outside this parameter range, such as when τ is equal to 10^{-3} s, the velocity map exhibits more wave branches, deviating noticeably from the in vivo experimental results. Finally, we can specify the viscoelastic parameter space: $10^{-5} < \tau < 10^{-4}$ s and $0.3 < g < 0.95$.

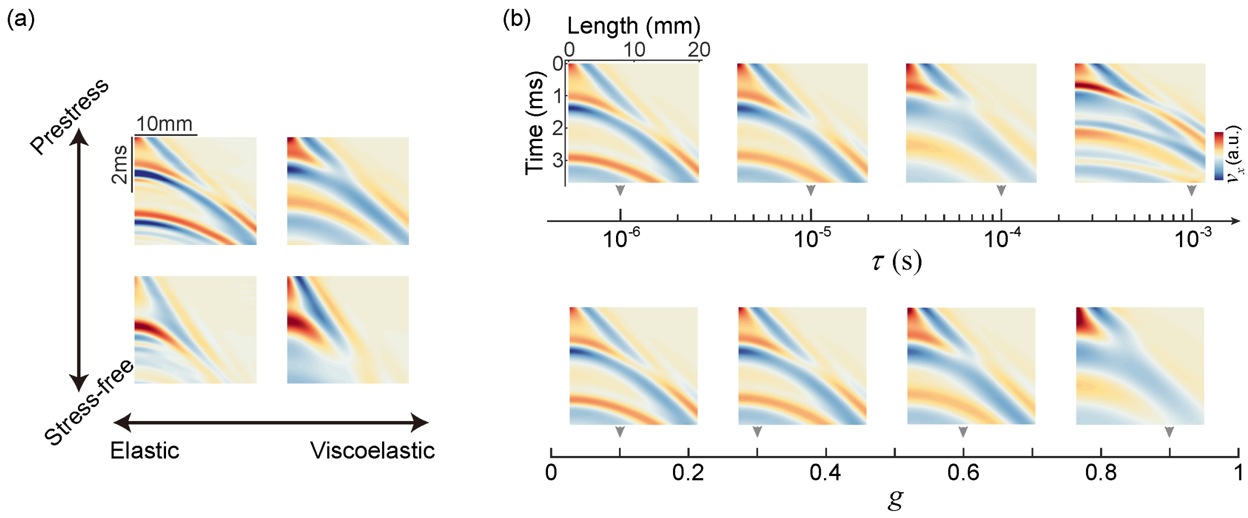

Fig. S25. Finite element results with varying pre-stress and material viscoelasticity. (a) Influence of pre-stress and viscoelasticity on the particle velocity map. **(b)** Evolution of the particle velocity maps with varying viscoelastic parameters g and τ.

Supplementary Note S18. Stability analysis and efficiency of the inversion method

The stability of the inversion method was studied by numerical experiment. Figure S26a shows the flowchart. Firstly, we gave standard dispersion curves at diastole and systole with known input parameters using Eq. (2). Random Gaussian noise with a magnitude of 3% was added to the dispersion curves (Fig. S26b). The noisy data were then utilized as input for the inversion method and the fitting values were obtained. In total, we generated 100 sets of noisy dispersion curves. By calculating the relative error (RE = |fitting-truth|/truth) between the input (truth) values and the fitting values, we evaluated the stability of the inversion method on each parameter. As listed in Table S3, the parameter α_a at both diastole and systole can be reliably inversed with a relative error of less than 5%. Among the viscous parameters, the estimation uncertainty of parameter τ is significantly higher (RE ~63%) compared to that of parameter g (RE ~17%). The relative error of the axial stress σ_a at both diastole and systole remains below 13%, indicating acceptable stability in the inversion of axial stress.

The efficiency of the inversion method was also studied by analyzing the computational cost. For a single set of *in vivo* experimental dispersion data (including two dispersion curves at diastole and systole), the CPU runtime of the inversion process was ~6 min on a standard laptop computer (Intel® Core(TM) i7-7700HQ CPU, 2.80 GHz with 16.0 GB RAM and 64 bit OS). This process can be further accelerated by optimizing the algorithm of inversion.

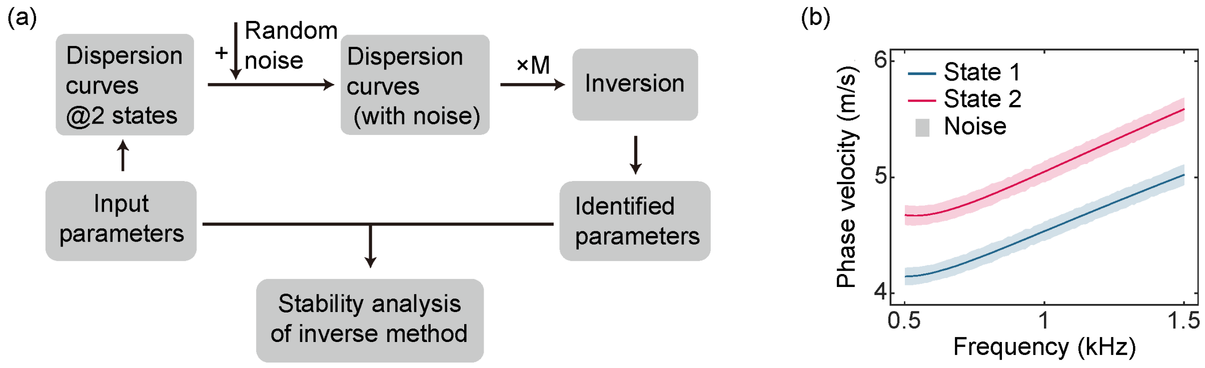

Fig. S26. Stability analysis of the inversion method. (a) Flowchart of the stability analysis. **(b)** Dispersion curves at two stress states with added noise.

Supplementary Note S19. Parameter sensitivity analysis of the dispersion of axial and circumferential guided waves

We performed the parameter sensitivity analysis on the dispersion curve of axial and circumferential guided waves. Figure S27a illustrates the influence of various parameters on axial guided wave dispersion. Viscous parameters g and τ have distinct influence on the dispersion curve at high frequencies (e.g. > 0.5 kHz). Among acoustoelastic parameters, α_a is the most dominant factor. γ has a secondary effect on the wave dispersion. β_a and α_c have minor effects on the dispersion. Based on the above relations, constraints were applied to β_a $(\beta_a = 4\alpha_a)$ and α_c $(\alpha_c = 1.5\alpha_a)$ during the inversion of material parameters using axial guided waves. This approximation improves the stability of inversion, and will not introduce significant errors in the estimation of the other parameters, i.e. α_a, γ, g and τ.

Figure S27b illustrates the influence of various parameters on circumferential guided wave dispersion. Similar to the axial dispersion, viscous parameters g and τ have distinct influence on the dispersion curve at high frequencies. Meanwhile, among the four acoustoelastic parameters, α_c serves as the most influential factor.

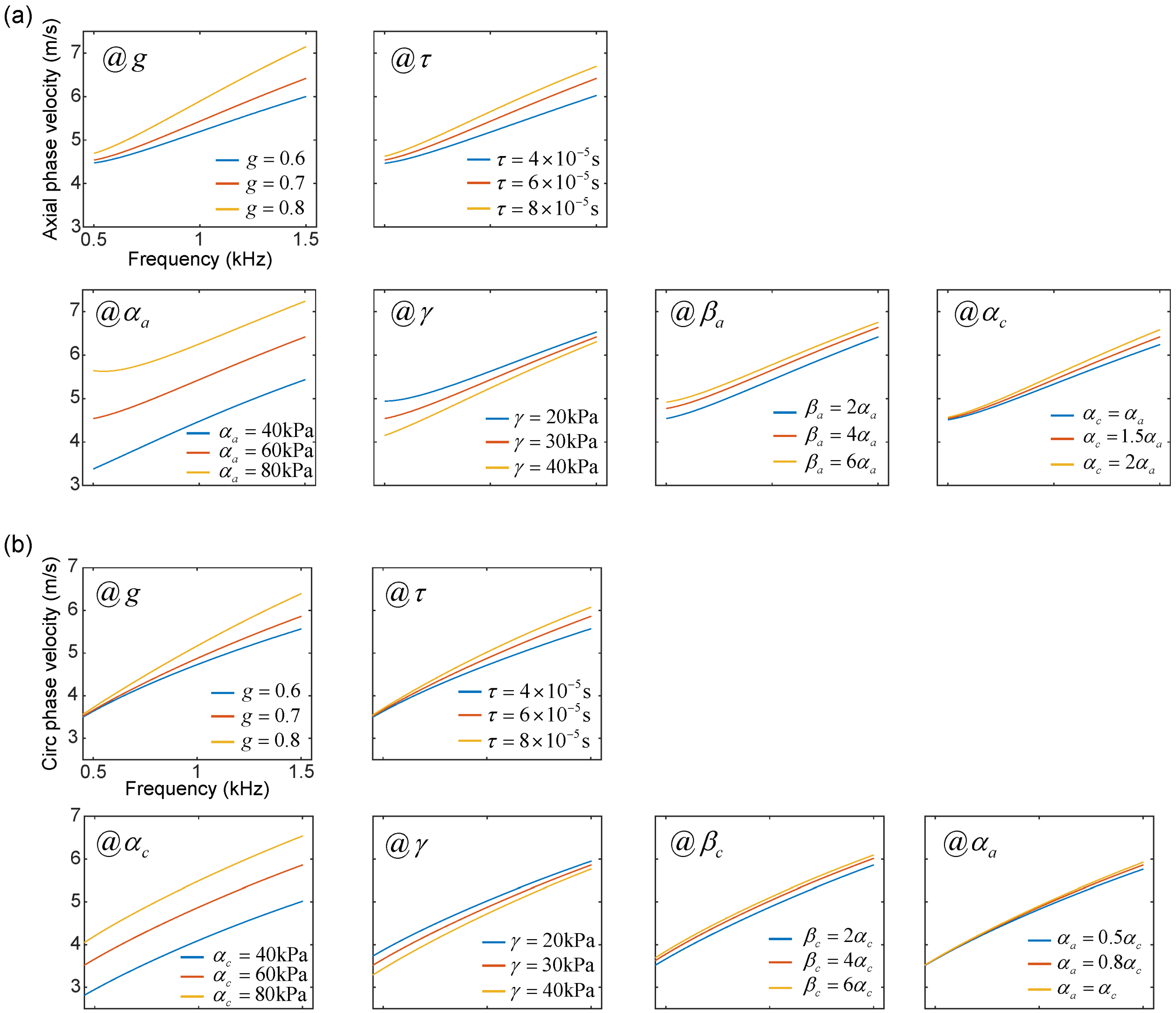

Fig. S27. Parameter sensitivity analysis of bidirectional wave dispersion. (a) Axial guided wave dispersion. **(b)** Circumferential guided wave dispersion.

Supplementary Tables

Table S1. Baseline characteristics of participants

	Young (n=30)	Older (n=14)	HT (n=8)
Age (y.o.)	20±2	51±8	55±5
Gender	30 M	12 M+2 F	5 M+3 F
Diastolic Pressure (mmHg)	66.0±8.7	76.9±8.3	86.1±6.9
Systolic Pressure (mmHg)	115.6±7.3	117.2±11.3	147.2±5.1
BMI (kg/m^2)	21.5±1.7	23.2±2.2	24.8±2.8
Middle radius at diastole (mm)	3.20±0.21	3.76±0.57	4.27±0.42
Middle radius at systole (mm)	3.51±0.23	3.92±0.59	4.44±0.45
Wall thickness at diastole (mm)	0.89±0.09	1.05±0.17	1.16±0.17

Table S2. Comparison of the mechanical parameters of porcine aorta tissues measured by the ultrasound method and the tensile test

	Stretch	α_a (kPa)	γ (kPa)	g	τ (10^{-5} s)	σ_a (kPa)
Ultrasound method	λ =1.23	73.7±3.5	36.4±0.9	0.72±0.02	5.6±0.15	37.3±4.0
	λ =1.31	101.6±5.8	44.0±7.4			57.6±2.8
Tensile test	λ =1.23	71.7	37.6	0.66*	7.7*	34.4
	λ =1.31	93.8	40.0			53.9
Relative error	λ =1.23	2.8%	3.2%	9.0%	27.3%	8.4%
	λ =1.31	8.3%	10.0%			6.9%

* The viscous parameters g and τ were obtained through curve fitting of the wave dispersion data at the stress-free state.

Table S3. Relative error (RE) of the inverse parameters compared to their input values

	$\alpha_{a,\mathrm{d}}$	γ_d	$\alpha_{a,\mathrm{s}}$	γ_s	g	τ	$\sigma_{a,\mathrm{d}}$	$\sigma_{a,\mathrm{s}}$
RE (%)	4.8	4.1	2.9	11.8	16.9	62.6	13.0	10.0

Supplementary Movies

Movie S1. Ultrasound imaging of arterial bidirectional guided waves throughout multiple cardiac cycles. This result was obtained from the right common carotid artery (CCA) of a young healthy volunteer (27 years old, male). The entire video consists of 36 frames uniformly distributed over ~2.5 s, each corresponding to an individual ultrasound measurement with an imaging duration of ~4 ms. **Top left:** B-mode image of the CCA in the longitudinal view. Ten acoustic radiation forces (ARFs) were applied sequentially from the anterior wall to the posterior wall. **Top right:** Spatiotemporal particle velocity map extracted along the anterior wall. The dashed line in the map indicates the axial guided wave propagation; the dot marks the arrival time t_1 of the circumferential guided wave. **Bottom:** Dynamic variations of bidirectional group wave velocities over cardiac cycles, along with synchronized electrocardiogram (ECG) signals.

REFERENCES AND NOTES

1. G. F. Mitchell, Arterial stiffness in aging: Does it have a place in clinical practice?: Recent advances in hypertension. *Hypertension* **77**, 768–780 (2021).

2. P. Boutouyrie, P. Chowienczyk, J. D. Humphrey, G. F. Mitchell, Arterial stiffness and cardiovascular risk in hypertension. *Circ. Res.* **128**, 864–886 (2021).

3. J. A. Chirinos, P. Segers, T. Hughes, R. Townsend, Large-artery stiffness in health and disease: JACC state-of-the-art review. *J. Am. Coll. Cardiol.* **74**, 1237–1263 (2019).

4. J. L. Cavalcante, J. A. Lima, A. Redheuil, M. H. Al-Mallah, Aortic stiffness. *J. Am. Coll. Cardiol.* **57**, 1511–1522 (2011).

5. S. Laurent, J. Cockcroft, L. Van Bortel, P. Boutouyrie, C. Giannattasio, D. Hayoz, B. Pannier, C. Vlachopoulos, I. Wilkinson, H. Struijker-Boudier, Expert consensus document on arterial stiffness: Methodological issues and clinical applications. *Eur. Heart J.* **27**, 2588–2605 (2006).

6. R. R. Townsend, I. B. Wilkinson, E. L. Schiffrin, A. P. Avolio, J. A. Chirinos, J. R. Cockcroft, K. S. Heffernan, E. G. Lakatta, C. M. McEniery, G. F. Mitchell, S. S. Najjar, W. W. Nichols, E. M. Urbina, T. Weber, American Heart Association Council on Hypertension, Recommendations for improving and standardizing vascular research on arterial stiffness: A scientific statement from the American Heart Association. *Hypertension* **66**, 698–722 (2015).

7. M. E. Safar, Arterial stiffness as a risk factor for clinical hypertension. *Nat. Rev. Cardiol.* **15**, 97–105 (2018).

8. A. E. Schutte, A. Kollias, G. S. Stergiou, Blood pressure and its variability: Classic and novel measurement techniques. *Nat. Rev. Cardiol.* **19**, 643–654 (2022).

9. J. D. Humphrey, M. A. Schwartz, Vascular mechanobiology: Homeostasis, adaptation, and disease. *Annu. Rev. Biomed. Eng.* **23**, 1–27 (2021).

10. J. D. Humphrey, E. R. Dufresne, M. A. Schwartz, Mechanotransduction and extracellular matrix homeostasis. *Nat. Rev. Mol. Cell Biol.* **15**, 802–812 (2014).

11. J. Humphrey, J. Eberth, W. Dye, R. Gleason, Fundamental role of axial stress in compensatory adaptations by arteries. *J. Biomech.* **42**, 1–8 (2009).

12. C. A. Nienaber, R. E. Clough, N. Sakalihasan, T. Suzuki, R. Gibbs, F. Mussa, M. P. Jenkins, M. M. Thompson, A. Evangelista, J. S. Yeh, Aortic dissection. *Nat. Rev. Dis. Primers* **2**, 1–18 (2016).

13. B. Staarmann, M. Smith, C. J. Prestigiacomo, Shear stress and aneurysms: A review. *Neurosurg. Focus* **47**, E2 (2019).

14. M. A. Meyers, P.-Y. Chen, A. Y.-M. Lin, Y. Seki, Biological materials: Structure and mechanical properties. *Prog. Mater. Sci.* **53**, 1–206 (2008).

15. A. J. Cocciolone, J. Z. Hawes, M. C. Staiculescu, E. O. Johnson, M. Murshed, J. E. Wagenseil, Elastin, arterial mechanics, and cardiovascular disease. *Am. J. Physiol. Heart Circul. Physiol.* **315**, H189–H205 (2018).

16. J. A. Chirinos, Arterial stiffness: Basic concepts and measurement techniques. *J. Cardiovasc. Transl. Res.* **5**, 243–255 (2012).

17. M. F. O'Rourke, J. A. Staessen, C. Vlachopoulos, D. Duprez, Clinical applications of arterial stiffness; definitions and reference values. *Am. J. Hypertens.* **15**, 426–444 (2002).

18. J. T. Pruijssen, C. L. de Korte, I. Voss, H. H. Hansen, Vascular shear wave elastography in atherosclerotic arteries: A systematic review. *Ultrasound Med. Biol.* **46**, 2145–2163 (2020).

19. M. Couade, M. Pernot, C. Prada, E. Messas, J. Emmerich, P. Bruneval, A. Criton, M. Fink, M. Tanter, Quantitative assessment of arterial wall biomechanical properties using shear wave imaging. *Ultrasound Med. Biol.* **36**, 1662–1676 (2010).

20. G.-Y. Li, Y. Jiang, Y. Zheng, W. Xu, Z. Zhang, Y. Cao, Arterial stiffness probed by dynamic ultrasound elastography characterizes waveform of blood pressure. *IEEE Trans. Med. Imaging* **41**, 1510–1519 (2022).

21. L. Marais, M. Pernot, H. Khettab, M. Tanter, E. Messas, M. Zidi, S. Laurent, P. Boutouyrie, Arterial stiffness assessment by shear wave elastography and ultrafast pulse wave imaging: Comparison with reference techniques in normotensives and hypertensives. *Ultrasound Med. Biol.* **45**, 758–772 (2019).

22. T. Roy, M. Urban, Y. Xu, J. Greenleaf, M. N. Guddati, Multimodal guided wave inversion for arterial stiffness: Methodology and validation in phantoms. *Phys. Med. Biol.* **66**, 115020 (2021).

23. M. Bernal, I. Nenadic, M. W. Urban, J. F. Greenleaf, Material property estimation for tubes and arteries using ultrasound radiation force and analysis of propagating modes. *J. Acoust. Soc. Am.* **129**, 1344–1354 (2011).

24. A. V. Astaneh, M. W. Urban, W. Aquino, J. F. Greenleaf, M. N. Guddati, Arterial waveguide model for shear wave elastography: Implementation and in vitro validation. *Phys. Med. Biol.* **62**, 5473–5494 (2017).

25. P. Dutta, M. W. Urban, O. P. Le Maître, J. F. Greenleaf, W. Aquino, Simultaneous identification of elastic properties, thickness, and diameter of arteries excited with ultrasound radiation force. *Phys. Med. Biol.* **60**, 5279–5296 (2015).

26. E. Maksuti, E. Widman, D. Larsson, M. W. Urban, M. Larsson, A. Bjällmark, Arterial stiffness estimation by shear wave elastography: Validation in phantoms with mechanical testing. *Ultrasound Med. Biol.* **42**, 308–321 (2016).

27. X. Zhang, R. R. Kinnick, M. Fatemi, J. F. Greenleaf, Noninvasive method for estimation of complex elastic modulus of arterial vessels. *IEEE Trans. Ultrason. Ferroelectr. Freq. Control* **52**, 642–652 (2005).

28. D. Marlevi, S. L. Mulvagh, R. Huang, J. K. DeMarco, H. Ota, J. Huston, R. Winter, T. A. Macedo, S. S. Abdelmoneim, M. Larsson, Combined spatiotemporal and frequency-dependent shear wave elastography enables detection of vulnerable carotid plaques as validated by MRI. *Sci. Rep.* **10**, 403 (2020).

29. Q. He, G.-Y. Li, F.-F. Lee, Q. Zhang, Y. Cao, J. Luo, Novel method for vessel cross-sectional shear wave imaging. *Ultrasound Med. Biol.* **43**, 1520–1532 (2017).

30. Y. Wang, H. Li, Y. Guo, W.-N. Lee, Bidirectional ultrasound elastographic imaging framework for non-invasive assessment of the non-linear behavior of a physiologically pressurized artery. *Ultrasound Med. Biol.* **45**, 1184–1196 (2019).

31. D. Shcherbakova, C. Papadacci, A. Swillens, A. Caenen, S. De Bock, V. Saey, K. Chiers, M. Tanter, S. Greenwald, M. Pernot, Supersonic shear wave imaging to assess arterial nonlinear behavior and anisotropy: Proof of principle via ex vivotesting of the horse aorta. *Adv. Mech. Eng.* **6**, 272586 (2014).

32. I. E. Hoefer, B. den Adel, M. J. Daemen, Biomechanical factors as triggers of vascular growth. *Cardiovasc. Res.* **99**, 276–283 (2013).

33. V. Thondapu, C. V. Bourantas, N. Foin, I.-K. Jang, P. W. Serruys, P. Barlis, Biomechanical stress in coronary atherosclerosis: Emerging insights from computational modelling. *Eur. Heart J.* **38**, 81–92 (2017).

34. I. Masson, H. Beaussier, P. Boutouyrie, S. Laurent, J. D. Humphrey, M. Zidi, Carotid artery mechanical properties and stresses quantified using in vivo data from normotensive and hypertensive humans. *Biomech. Model. Mechanobiol.* **10**, 867–882 (2011).

35. I. Masson, P. Boutouyrie, S. Laurent, J. D. Humphrey, M. Zidi, Characterization of arterial wall mechanical behavior and stresses from human clinical data. *J. Biomech.* **41**, 2618–2627 (2008).

36. F. P. Beer, E. R. Johnston, J. T. DeWolf, D. F. Mazurek, S. Sanghi, *Mechanics of Materials* (Mcgraw-Hill New York, 1992), vol. 1.

37. J. Humphrey, S. Na, Elastodynamics and arterial wall stress. *Ann. Biomed. Eng.* **30**, 509–523 (2002).

38. Z. Zhang, G.-Y. Li, Y. Jiang, Y. Zheng, A. L. Gower, M. Destrade, Y. Cao, Noninvasive measurement of local stress inside soft materials with programmed shear waves. *Sci. Adv.* **9**, eadd4082 (2023).

39. S. Laurent, P. Boutouyrie, Arterial stiffness and hypertension in the elderly. *Front. Cardiovasc. Med.* **7**, 544302 (2020).

40. W. F. Boron, E. L. Boulpaep, *Medical Physiology* (Elsevier Health Sciences, 2016).

41. G. F. Mitchell, S.-J. Hwang, R. S. Vasan, M. G. Larson, M. J. Pencina, N. M. Hamburg, J. A. Vita, D. Levy, E. J. Benjamin, Arterial stiffness and cardiovascular events: The Framingham Heart Study. *Circulation* **121**, 505–511 (2010).

42. C. Wang, X. Li, H. Hu, L. Zhang, Z. Huang, M. Lin, Z. Zhang, Z. Yin, B. Huang, H. Gong, S. Bhaskaran, Y. Gu, M. Makihata, Y. Guo, Y. Lei, Y. Chen, C. Wang, Y. Li, T. Zhang, Z. Chen, A. P. Pisano, L. Zhang, Q. Zhou, S. Xu, Monitoring of the central blood pressure waveform via a conformal ultrasonic device. *Nat. Biomed. Eng.* **2**, 687–695 (2018).

43. S. Zhou, G. Park, K. Longardner, M. Lin, B. Qi, X. Yang, X. Gao, H. Huang, X. Chen, Y. Bian, H. Hu, R. S. Wu, W. Yue, M. Li, C. Lu, R. Wang, S. Qin, E. Tasali, T. Karrison, I. Thomas, B. Smarr, E. B. Kistler, B. A. I. Khiami, I. Litvan, S. Xu, Clinical validation of a wearable ultrasound sensor of blood pressure. *Nat. Biomed. Eng.* **9**, 865–881 (2025).

44. R. W. Ogden, "Incremental statics and dynamics of pre-stressed elastic materials," in *Waves in Nonlinear Pre-Stressed Materials* (Springer, 2007), pp. 1–26.

45. Y. Jiang, S. Ma, Y. Cao, Guided wave elastography of jugular veins: Theory, method and in vivo experiment. *J. Biomech.* **160**, 111828 (2023).

46. D. Ran, J. Dong, H. Li, W.-N., Spontaneous extension wave for in vivo assessment of arterial wall anisotropy. *Am. J. Physiol.-Heart Circul. Physiol.* **320**, H2429–H2437 (2021).

47. Y. Wang, W.-N. Lee, Non-invasive estimation of localized dynamic luminal pressure change by ultrasound elastography in arteries with normal and abnormal geometries. *IEEE Trans. Biomed. Eng.* **68**, 1627–1637 (2020).

48. A. M. Zakrzewski, B. W. Anthony, Noninvasive blood pressure estimation using ultrasound and simple finite element models. *IEEE Trans. Biomed. Eng.* **65**, 2011–2022 (2017).

49. G. A. Holzapfel, T. C. Gasser, R. W. Ogden, A new constitutive framework for arterial wall mechanics and a comparative study of material models. *J. Elast.* **61**, 1–48 (2000).

50. A. V. Kamenskiy, Y. A. Dzenis, S. A. J. Kazmi, M. A. Pemberton, I. I. Pipinos, N. Y. Phillips, K. Herber, T. Woodford, R. E. Bowen, C. S. Lomneth, J. N. MacTaggart, Biaxial mechanical properties of the human thoracic and abdominal aorta, common carotid, subclavian, renal and common iliac arteries. *Biomech. Model. Mechanobiol.* **13**, 1341–1359 (2014).

51. L. Horný, M. Netušil, T. Voňavková, Axial prestretch and circumferential distensibility in biomechanics of abdominal aorta. *Biomech. Model. Mechanobiol.* **13**, 783–799 (2014).

52. G. Sommer, G. A. Holzapfel, 3D constitutive modeling of the biaxial mechanical response of intact and layer-dissected human carotid arteries. *J. Mech. Behav. Biomed. Mater.* **5**, 116–128 (2012).

53. C. Wang, X. Chen, L. Wang, M. Makihata, H.-C. Liu, T. Zhou, X. Zhao, Bioadhesive ultrasound for long-term continuous imaging of diverse organs. *Science* **377**, 517–523 (2022).

54. H.-C. Liu, Y. Zeng, C. Gong, X. Chen, P. Kijanka, J. Zhang, Y. Genyk, H. Tchelepi, C. Wang, Q. Zhou, X. Zhao, Wearable bioadhesive ultrasound shear wave elastography. *Sci. Adv.* **10**, eadk8426 (2024).

55. L. E. Niklason, J. H. Lawson, Bioengineered human blood vessels. *Science* **370**, eaaw8682 (2020).

56. C. Laschi, B. Mazzolai, M. Cianchetti, Soft robotics: Technologies and systems pushing the boundaries of robot abilities. *Sci. Robot.* **1**, eaah3690 (2016).

57. M. Cianchetti, C. Laschi, A. Menciassi, P. Dario, Biomedical applications of soft robotics. *Nat. Rev. Mater.* **3**, 143–153 (2018).

58. R. Kelly, D. Fitchett, Noninvasive determination of aortic input impedance and external left ventricular power output: A validation and repeatability study of a new technique. *J. Am. Coll. Cardiol.* **20**, 952–963 (1992).

59. G.-Y. Li, Y. Zheng, Y. Liu, M. Destrade, Y. Cao, Elastic Cherenkov effects in transversely isotropic soft materials-I: Theoretical analysis, simulations and inverse method. *J. Mech. Phys. Solids* **96**, 388–410 (2016).

60. T. Loupas, J. Powers, R. W. Gill, An axial velocity estimator for ultrasound blood flow imaging, based on a full evaluation of the Doppler equation by means of a two-dimensional autocorrelation approach. *IEEE Trans. Ultrason. Ferroelectr. Freq. Control* **42**, 672–688 (1995).

61. J. Bercoff, M. Tanter, M. Fink, Supersonic shear imaging: A new technique for soft tissue elasticity mapping. *IEEE Trans. Ultrason. Ferroelectr. Freq. Control* **51**, 396–409 (2004).

62. M. Cinthio, T. Jansson, A. Eriksson, Å. R. Ahlgren, H. W. Persson, K. Lindström, Evaluation of an algorithm for arterial lumen diameter measurements by means of ultrasound. *Med. Biol. Eng. Comput.* **48**, 1133–1140 (2010).

63. J. Wikstrand, Methodological considerations of ultrasound measurement of carotid artery intima–media thickness and lumen diameter. *Clin. Physiol. Funct. Imaging* **27**, 341–345 (2007).

64. N. C. Rouze, M. H. Wang, M. L. Palmeri, K. R. Nightingale, Parameters affecting the resolution and accuracy of 2-D quantitative shear wave images. *IEEE Trans. Ultrason. Ferroelectr. Freq. Control* **59**, 1729–1740 (2012).

65. N. C. Rouze, M. H. Wang, M. L. Palmeri, K. R. Nightingale, Robust estimation of time-of-flight shear wave speed using a radon sum transformation. *IEEE Trans. Ultrason. Ferroelectr. Freq. Control* **57**, 2662–2670 (2010).

66. F. Giovanniello, M. Asgari, I. D. Breslavsky, G. Franchini, G. A. Holzapfel, M. Tabrizian, M. Amabili, Development and mechanical characterization of decellularized scaffolds for an active aortic graft. *Acta Biomater.* **160**, 59–72 (2023).

67. T. C. Gasser, R. W. Ogden, G. A. Holzapfel, Hyperelastic modelling of arterial layers with distributed collagen fibre orientations. *J. R. Soc. Interface* **3**, 15–35 (2006).

68. Y.-c. Fung, *Biomechanics: Mechanical Properties of Living Tissues* (Springer Science & Business Media, 2013).

69. G. Franchini, I. D. Breslavsky, G. A. Holzapfel, M. Amabili, Viscoelastic characterization of human descending thoracic aortas under cyclic load. *Acta Biomater.* **130**, 291–307 (2021).

70. G. Franchini, I. D. Breslavsky, F. Giovanniello, A. Kassab, G. A. Holzapfel, M. Amabili, Role of smooth muscle activation in the static and dynamic mechanical characterization of human aortas. *Proc. Natl. Acad. Sci.* **119**, e2117232119 (2022).

71. K. Parker, T. Szabo, S. Holm, Towards a consensus on rheological models for elastography in soft tissues. *Phys. Med. Biol.* **64**, 215012 (2019).

72. S. Baek, R. L. Gleason, K. Rajagopal, J. Humphrey, Theory of small on large: Potential utility in computations of fluid–solid interactions in arteries. *Comput. Methods Appl. Mech. Eng.* **196**, 3070–3078 (2007).

73. G. A. Holzapfel, J. A. Niestrawska, R. W. Ogden, A. J. Reinisch, A. J. Schriefl, Modelling non-symmetric collagen fibre dispersion in arterial walls. *J. R. Soc. Interface* **12**, 20150188 (2015).

74. M. Amabili, M. Asgari, I. D. Breslavsky, G. Franchini, F. Giovanniello, G. A. Holzapfel, Microstructural and mechanical characterization of the layers of human descending thoracic aortas. *Acta Biomater.* **134**, 401–421 (2021).

75. J. Li, J. L. Rose, Natural beam focusing of non-axisymmetric guided waves in large-diameter pipes. *Ultrasonics* **44**, 35–45 (2006).

76. N. R. Hugenberg, T. Roy, H. Harrigan, M. Capriotti, H.-K. Lee, M. Guddati, J. F. Greenleaf, M. W. Urban, W. Aquino, Toward improved accuracy in shear wave elastography of arteries through controlling the arterial response to ultrasound perturbation in-silico and in phantoms. *Phys. Med. Biol.* **66**, 235008 (2021).

77. M. Capriotti, T. Roy, N. R. Hugenberg, H. Harrigan, H.-C. Lee, W. Aquino, M. Guddati, J. F. Greenleaf, M. W. Urban, The influence of acoustic radiation force beam shape and location on wave spectral content for arterial dispersion ultrasound vibrometry. *Phys. Med. Biol.* **67**, 135002 (2022).

78. G.-Y. Li, Q. He, G. Xu, L. Jia, J. Luo, Y. Cao, An ultrasound elastography method to determine the local stiffness of arteries with guided circumferential waves. *J. Biomech.* **51**, 97–104 (2017).

79. Y. Jiang, G.-Y. Li, Z. Zhang, S. Ma, Y. Cao, S.-H. Yun, Incremental dynamics of prestressed viscoelastic solids and its applications in shear wave elastography. *Int. J. Eng. Sci.* **215**, 104310 (2025).

80. M. J. Berliner, R. Solecki, Wave propagation in fluid-loaded, transversely isotropic cylinders. Part I. Analytical formulation. *J. Acoust. Soc. Am.* **99**, 1841–1847 (1996).

81. P. R. Hoskins, Physical properties of tissues relevant to arterial ultrasound imaging and blood velocity measurement. *Ultrasound Med. Biol.* **33**, 1527–1539 (2007).

82. D. C. Gazis, Three-dimensional investigation of the propagation of waves in hollow circular cylinders. I. Analytical foundation. *J. Acoust. Soc. Am.* **31**, 568–573 (1959).

83. F. Schroeder, S. Polzer, M. Slažanský, V. Man, P. Skácel, Predictive capabilities of various constitutive models for arterial tissue. *J. Mech. Behav. Biomed. Mater.* **78**, 369–380 (2018).

84. M. Destrade, “Incremental equations for soft fibrous materials,” in *Nonlinear Mechanics of Soft Fibrous Materials* (Springer, 2015), pp. 233–267.
